# Anti-tumor activity of valproic acid in EBV-positive Burkitt lymphoma via PFN2-associated PI3K/AKT pathway repression

**DOI:** 10.3389/fphar.2026.1925552

**Published:** 2026-09-14

**Authors:** Yongliang Zheng, Yuxiu Yang, Shouhua Zhang, Rui Liu, Zujie Lin, Yuanqiao He, Yaoling Luo, Aili He

**Affiliations:** 1 Department of Hematology, The Second Affiliated Hospital of Xi’an Jiaotong University, Xi’an, Shaanxi, China; 2 Department of Hematology, The Affiliated Hospital of Jinggangshan University, Ji’an, Jiangxi, China; 3 School of Pharmacy, Jiangxi Science and Technology Normal University, Nanchang, Jiangxi, China; 4 Department of General Surgery, Jiangxi Provincial Children’s Hospital, The Affiliated Children’s Hospital of Nanchang Medical College, Nanchang, Jiangxi, China; 5 Center of Laboratory Animal Science, Nanchang University, Nanchang, Jiangxi, China; 6 Key Laboratory of New Drug Evaluation and Transformation of Jiangxi Province, Nanchang, Jiangxi, China; 7 Clinical Medicine Research Center, First Affiliated Hospital of Gannan Medical University, Ganzhou, Jiangxi, China; 8 National-Local Joint Engineering Research Center of Biodiagnostics & Biotherapy, The Second Affiliated Hospital of Xi’an Jiaotong University, Xi’an, Shaanxi, China; 9 Department of Tumor and Immunology, Precision Medical Institute, Xi’an Jiaotong University, Xi’an, Shaanxi, China; 10 Xi’an Key Laboratory of Hematological Diseases, Xi’an, Shaanxi, China

**Keywords:** Burkitt lymphoma, valproic acid, PFN2, PI3K, AKT

## Abstract

**Background:**

Burkitt lymphoma (BL) has limited therapies and poor prognosis in elderly or relapsed patients. Valproic acid (VPA) exhibits broad anti-tumor activity, but its quantitative efficacy and molecular mechanism in EBV-positive BL remain undefined.

**Methods:**

VPA activity in Daudi, Raji, Namalwa BL cell lines (n = 3 biological replicates per assay) was assessed using CCK-8 viability (24 h/48 h IC_50_ values), EdU proliferation, PI cell-cycle, and Annexin V/PI dual-staining apoptosis assays. *In vivo* efficacy was tested in Namalwa CDX (control n = 5, VPA n = 6) and three independent EBV-positive PDX models (6 mice/group for each PDX line), with daily 400 mg/kg intraperitoneal VPA administration for 1–3 weeks; *ex vivo* drug response of PDX fragments was quantified via hydrogel embedded histoculture drug sensitivity test (HDST). RNA-seq was conducted on PDX No.1 tumor tissues (VPA vs. saline control, n = 3 per group). PFN2 and PI3K/AKT pathway alterations were validated by RT-qPCR, Western blot, PFN2 knockdown/overexpression, and PI3K/AKT inhibitor combination assays.

**Results:**

24 h IC_50_ values of VPA were 20.15, 4.672, 7.769 mM for Daudi, Raji, Namalwa, while 48 h IC_50_ decreased to 4.891, 2.283, 0.4836 mM. At 2 mM VPA treatment for 24 h, total apoptosis rates increased by 14.22% (Daudi), 10.20% (Raji), and 18.68% (Namalwa). VPA induced G0/G1 cell-cycle arrest across all 3 cell lines. In CDX and three PDX models, VPA treatment reduced tumor weight (CDX: control 1.132 g vs. VPA 0.365 g, *p* = 0.0207, No.1: control 1.450 g vs. VPA 1.013 g, *p* = 0.049, No.2: control 1.283 g vs. VPA 0.913 g, *p* = 0.0295, No.3: control 1.371 g vs. VPA 0.9239, *p* = 0.0062) without body weight loss. RNA-seq analysis identified PFN2 as an exploratory candidate transcript with nominal transcriptional downregulation upon VPA administration (log_2_FC = −0.3213, raw p = 0.0355, FDR = 0.6149), and KEGG enrichment revealed perturbation of the PI3K/AKT signaling pathway. VPA reduced PFN2 mRNA and protein abundance *in vitro* and *in vivo*. PFN2 knockdown recapitulated proliferation suppression and reduced p-PI3K/p-AKT levels; PFN2 overexpression partially restored cell proliferation and PI3K/AKT phosphorylation, which could be attenuated by VPA treatment. Co-administration of VPA with idelalisib or capivasertib produced stronger anti-proliferative effects of BL.

**Conclusion:**

VPA exerts potent anti-tumor activity against EBV-positive BL *in vitro* and in multiple xenograft models, associated with PFN2 downregulation and suppressed PI3K/AKT signaling.

## 1 Introduction

Burkitt lymphoma (BL) is a highly aggressive B-cell non-Hodgkin lymphoma (Gastwirt and Roschewski, 2018). BL is divided into three main subtypes: endemic, sporadic, and immunodeficiency-associated types (Ferry, 2006). Endemic Burkitt lymphoma primarily affects African children, typically between the ages of 4 and 7, and is strongly associated with Epstein-Barr Virus (EBV) infection. Sporadic Burkitt lymphoma occurs globally without a specific geographical or climatic pattern. Immunodeficiency-associated Burkitt lymphoma is linked to HIV infection (Casulo and Friedberg, 2015). A hallmark of Burkitt lymphoma is the presence of chromosomal translocations involving the MYC gene (8q24) and one of the immunoglobulin loci—most commonly IGH (14q32), but also IGK (2p12) or IGL (22q11) (Sharma et al., 2022). Immunophenotypically, BL is characterized by the expression of CD20, CD10, BCL6, and a high proliferation index indicated by Ki67 positivity (McClure et al., 2005). Histologically, BL shows diffuse sheets of medium-sized monomorphic B cells with multiple nucleoli and a “starry sky” pattern. Contemporary clinical management of BL relies on risk-adapted intensive multi-agent combination chemotherapy, including CODOX-M/IVAC, Hyper-CVAD, and dose-adjusted EPOCH-R, often combined with rituximab and CNS-directed prophylaxis/therapy, combined with mandatory central nervous system (CNS)-directed prophylaxis to eliminate occult central nervous system seeding risk, and this intensive regimen achieves durable cure for most pediatric and young adult patients. However, Older patients (≥60 years) and those with relapse tend to have a poorer prognosis (Casulo and Friedberg, 2015) (Yustein and Dang, 2007). In one study, the 5-year relative survival rate for older patients was 28.9%, significantly lower than for children and adults (Mukhtar et al., 2017). In another study, patients who relapsed had a median OS of just 3.2 months and a 2-year overall survival rate of 17.2% (Manji et al., 2021). A subset of BL patients presents with baseline bone marrow or central nervous system involvement at diagnosis, and high-risk patients carry an elevated risk of systemic dissemination to these sanctuary sites during disease progression, which necessitates mandatory CNS-directed prophylaxis as part of standard treatment. Corresponding clinical references are supplemented.

VPA is a recognized Class I histone deacetylase inhibitor with FDA-approved indications for epilepsy, bipolar mania and migraine prevention (Shah et al., 2013). Current studies have indicated that VPA can be used alone or in combination with chemotherapeutic agents or radiotherapy for cancer treatment (Shah et al., 2013; Michaelis et al., 2007; Heers et al., 2018). VPA has been shown to inhibit the proliferation, survival, cell migration and hormone receptor expression of breast cancer cells (). Additionally, VPA could enhance fludarabine-induced apoptosis mediated by reactive oxygen species (ROS) and reduce AKT and ATM activation in B-cell-lymphoid neoplastic cells (Yoon et al., 2014). However, its efficacy and anti-cancer mechanisms in BL remain unclear.

PFN2 -encoded Profilin is a 12–15 kDa actin-binding protein found in eukaryotes (Ding et al., 2012). PFN2 is primarily localized in the cytoplasm and binds to actin, playing a crucial role in the structure and dynamics of the cytoskeleton (Ding et al., 2012)^.^ Recent studies have demonstrated a significant correlation between PFN2 expression and cancer progression (Liu et al., 2018; Ling et al., 2021). PFN2 is notably upregulated in triple-negative breast cancer (TNBC), and its overexpression is associated with a poorer prognosis. In TNBC, PFN2 could facilitate tumor cell proliferation, migration, and invasion by modulating epithelial-mesenchymal transition (EMT) processes (Ling et al., 2021). Furthermore, elevated levels of PFN2 have been observed in both low-grade and high-grade esophageal cancer, where it contributes to cancer progression and metastasis, serving as a potential biomarker for poor clinical outcomes (Cui et al., 2016). The PI3K signaling pathway plays a critical role in cell survival, proliferation, migration, and metabolism (Courtney et al., 2010). PI3K catalyzes the production of phosphatidylinositol 3,4,5-trisphosphate (PIP3), which activates downstream signaling molecules such as AKT, and influences various biological processes in cells, which is aberrantly activated in cancers, contributing to the occurrence and progression of tumors (He et al., 2021; Alzahrani, 2019). The PI3K/AKT signaling pathway is aberrantly activated in many B-cell malignancies, including BL, where it promotes cell survival and proliferation (Karaduman et al., 2025). PFN2 regulates the palmitic acid metabolism of lung cancer through the PI3K/FABP5/AKT axis, promoting EMT, which reveals PFN2 as a potential therapeutic target in lung cancer (Chi, 2023). Although PFN2 has been extensively studied in solid tumors, its role in BL and other lymphoid malignancies remains largely unexplored. The Human Protein Atlas shows variable PFN2 expression in lymphoma cells, but functional studies in BL are lacking.

In this study, we investigated the efficacy of VPA in BL using *in vitro* cell-line studies, a CDX model, and three PDX models. Then used an unbiased RNA-seq discovery approach to identify molecular targets of VPA in BL. Among the differentially expressed genes, PFN2 emerged as a candidate mediator. And functional validation through qPCR, Western blotting, and PFN2 knockdown/overexpression experiments were followed. Our central hypothesis was that VPA inhibits BL growth by modulating signaling pathways through transcriptional regulation.

## 2 Methods and materials

### 2.1 Cell lines

The Daudi and Raji cell lines were obtained from National Collection of Authenticated Cell Cultures (Shanghai, China) and identified by STR. Namalwa cell line was obtained from Baidi Biotech company (Zhejiang, China) and identified by STR. Furthermore, all cell lines were routinely tested and confirmed to be free of *mycoplasma* contamination using a PCR-based detection method. Cells were cultured in RPMI1640 medium containing 1% penicillin and 1% streptomycin and 15% fetal bovine serum at 37 °C.

### 2.2 Animal experiments

The NOD-SCID mice (6–8 weeks old) were purchased from Hangzhou Ziyuan experimental animal Technology Co., LTD. (Hangzhou, China) and housed at 22 °C–24 °C, 50%–60% humidity, and a 12-h light/dark cycle in a specific pathogen-free (SPF) animal facility. At experiment endpoints, all animals were euthanized using CO_2_ (20% of the chamber volume per minute) anesthesia. For the CDX and PDX *in vivo* experiments, sample sizes (5–6 mice per group) were determined by practical considerations of tumor-bearing mouse availability and ethical minimization of animal use, consistent with common practice in preclinical xenograft studies (Navarro-Yepes et al., 2023; Eckel-Passow et al., 2021). The experimental protocol was approved by the Institutional Animal Care and Use Committee (IACUC) of Jinggangshan University Affiliated Hospital (approval No. 2020 lun pi No.19).

### 2.3 Patient sample

This study was carried out in accordance with the principles of the Declaration of Helsinki. The Patient’s tissue samples were collected with the approval of the ethical committee of Jinggangshan University Affiliated Hospital (approval No. 2020 lun pi No. 20). The recruited patient was required to sign an informed consent before sample collection.

### 2.4 Cell proliferation assay by CCK-8

Daudi, Raji and Namalwa cells were seeded into 96-well plates at a density of 5,000–7,000 cells/well. Cells were cultured in logarithmic growth phase for 1 day, then VPA (0,2,4,6,8,16 or 32 mM) and Saline was added to each well, and the cells were incubated in the incubator for 24 h or 48 h. Then, 10% of CCK8 solution was added to each well, and cells were incubated for 2–4 h. The optical density (OD) of each well at 450 nm was then measured using a microplate reader. Cell viability and IC_50_ values were calculated based on the OD value. Three independent experiments were performed.

### 2.5 Flow cytometry analysis

All flow cytometry assays (EdU proliferation, apoptosis, DNA content cell-cycle profiling) were performed with 3 independent biological replicates, ≥10,000 single-cell events acquired per sample, 2 technical replicates for each biological replicate.

#### 2.5.1 EdU proliferation assay

Cell proliferation was assessed using a Click-iT EdU kit (KeyGEN BioTECH, KGA9601-100), wherein cells pulsed with 10 µM EdU for 2 h were fixed, permeabilized, and subjected to a fluorescent click reaction before flow cytometric analysis, with the proliferation rate quantified as the percentage of EdU-positive cells.

#### 2.5.2 Apoptosis assay

For apoptosis analysis, cells were stained with Annexin V-FITC and propidium iodide (PI) (KeyGEN BioTECH, KGA1102-100) according to the manufacturer’s instructions; untreated cells and cells treated with 1 µM staurosporine for 6 h served as negative and positive controls, respectively. For both assays, data from at least 10,000 events were acquired on a flow cytometer, and analysis was performed using FlowJo software: single cells were gated based on FSC-H/FSC-A, and apoptotic cells were identified as Annexin V^+^/PI^−^ (early apoptotic) and Annexin V^+^/PI^+^ (late apoptotic), with the total apoptosis rate presented as the sum of these two populations.

#### 2.5.3 DNA content cell-cycle analysis

Daudi, Raji and Namalwa suspension cells were seeded at densities specified in Section 2.4 and treated with serially diluted VPA (0, 0.5, 1, 2 mM) or PBS vehicle control for 24 h at 37 °C with 5% CO_2_; cells were harvested by centrifugation at 300 *g* for 5 min at 4 °C, washed twice with pre-cooled PBS, fixed in ice-cold 70% ethanol and stored at −20 °C overnight for ≥12 h. After ethanol removal via centrifugation and two additional cold PBS washes, cell pellets were resuspended in 200 μL PI staining buffer containing 50 μg/mL propidium iodide (PI) and 100 μg/mL RNase A (Solarbio, P8080) to eliminate RNA-associated non-specific PI binding, followed by 30 min room-temperature incubation in the dark. Stained samples were analyzed on a BD FACSCanto II flow cytometer with a minimum of 10,000 single-cell events recorded per sample; doublets and debris were excluded using FSC-A versus FSC-H gating, and DNA content histograms were modeled with FlowJo v10 to calculate the percentage of cells in G0/G1, S, and G2/M phases. All cell-cycle experiments included vehicle-treated baseline controls and were performed with 3 independent biological replicates, each with two technical staining replicates.

### 2.6 BL CDX models

Suspensions of Namalwa cells (2.5 × 10^^^6/200 μL in 50% matrigel) were subcutaneously injected into female NOD-SCID mice to establish xenograft tumors. When the Namalwa xenograft tumors reached a size of 50–100 mm^3^ size, the mice were randomly divided into 2 groups (control n = 5, VPA n = 6). Mice with inappropriate tumors’ size were excluded from the study. One group was treated with VPA (dissolved in physiological saline, 400 mg/kg/200 μL, intraperitoneally, once daily as previously reported (Pragnya et al., 2014) and the other group received Saline (200 μL,i.p., q. d., 1–3 weeks). Mice with baseline tumor volume out of 30–150 mm^3^ were excluded before grouping. Group assignment was sealed before animal grouping. Daily body weight, activity, fur state inspection; all abnormal animals were recorded. Body weight and tumor size were measured every 2–3 days, and tumor volume was calculated by (length × width^2^)/2. Tumor weight was recorded one the last treatment day. The researchers measuring tumor volume, weighing mice and analyzing data were blinded to treatment grouping. Calculated based on prior published xenograft tumor growth variation to achieve 80% statistical power at α = 0.05. Simple stratified randomization by baseline tumor volume via GraphPad random number generator. Any mouse with tumor volume ≥1,500 mm^3^ would be euthanized immediately and recorded separately in dataset; no animals reached this endpoint in our study. Intraperitoneal injection of pentobarbital sodium, administered at a dose of 50 mg/kg for all invasive operations (tumor sampling, tissue dissection).

### 2.7 BL PDX model construction and drug sensitivity test in PDX model

The first patient (No.1) was a 10-year-old boy, EBER(+), C-MYC(+), which was diagnosed with primary BL carcinoma with a clinical TNM stage of IV. The second patient (No.2) was a 12-year-old girl, EBER(+), C-MYC(+), which was diagnosed with metastatic BL. The third patient (No.3) was a 14-year-old girl, EBER(+), C-MYC(+), which was diagnosed with primary BL. The BL PDX model construction and drug sensitivity test in PDX model methods were same as the previously reported (Zeng et al., 2024). Briefly, fresh surgically resected BL tissue was cut into small fragments of 2 mm^3^ and aseptically inoculated subcutaneously to the scapula of NOD-SCID mice. The gender selection of mice is determined according to the gender of the patient. When the tumor has grown to a certain size, part of the tumor tissue is removed, and when the tumor grew to a certain size, part of the tumor tissue was removed and re-implanted into a new immunodeficient mouse for multigenerational passage. The third generation (P_3_) tumor volume reached 30–150 mm^3^, the PDX mice were randomly divided into two groups to treated with VPA (n = 6, dissolved in physiological saline, 400 mg/kg/200 μL, intraperitoneally, once daily as reported (Pragnya et al., 2014)) and saline (n = 6, 200 μL, i. p., q. d. 1–3weeks). Mice with baseline tumor volume out of 30–150 mm^3^ were excluded before grouping, we supplemented the exact number of excluded individuals in text. Group assignment was sealed before animal grouping. Daily body weight, activity, fur state inspection; all abnormal animals were recorded. Body weight and tumor size were measured every 2–3 days, and tumor volume was calculated by (length × width^2^)/2. Tumor weight was recorded one the last treatment day. The researchers measuring tumor volume, weighing mice and analyzing data were blinded to treatment grouping. Calculated based on prior published xenograft tumor growth variation to achieve 80% statistical power at α = 0.05. Simple stratified randomization by baseline tumor volume via GraphPad random number generator. Any mouse with tumor volume ≥1,500 mm^3^ would be euthanized immediately and recorded separately in dataset; no animals reached this endpoint in our study. Intraperitoneal injection of pentobarbital sodium, administered at a dose of 50 mg/kg for all invasive operations (tumor sampling, tissue dissection).

### 2.8 Hydrogel embedded histoculture drug sensitivity test (HDST)

The PDX tissues were dissected into small fragments measuring 3 mm^3^. And then, the small fragments tumor tissues were cultured in 96-well plates wrapped with water gel including different concentrations of VPA (0.25, 0.5, 1, 2, 4, 8, and 16 mM). After incubating the PDX fragments for 5 or 7 days, fresh medium containing an equal amount of the drug was replaced. And then, the viability of the cells was assessed using the CCK8 assay. Independent tumor fragments derived from different PDX donor mice are defined as biological replicates, and duplicate wells seeded with the same tumor fragment are technical replicates. A total of 5–6 independent HDST biological experiments were completed for each of the three PDX models in different concentrations of VPA. The absorbance value of each individual tumor fragment was defined as the independent statistical unit for IC_50_ curve fitting and calculation.

### 2.9 H&E staining and immunohistochemistry (IHC)

The H&E staining and IHC was done as previously reported (Yuanqiao et al., 2023). Briefly, Patient-derived tumor tissues and those from PDX models were formalin-fixed and subsequently embedded in paraffin. Sections of these specimens, 4 μm in thickness, were prepared on dry slides, stained with hematoxylin–eosin (H&E) by automatic slide stainer (DAKEWE Slide stainer DP360 Series) and IHC by automatic scientific research dyeing machine (Leika). The antibody was including MYC (Gene Tech), BCL-6(Gene Tech), Ki67(Gene Tech).

### 2.10 PCR

The PCR was done as previously reported (Yuanqiao et al., 2023). For PCR confirmation of PDX human origin, the 327-bp amplicon targeted the human GAPDH gene (primers: GGC​TCT​TAA​AAA​GTG​CAG​GGT​C and ATG​GTA​CAT​GAC​AAG​GTG​CGG), and the 772-bp amplicon targeted the human β-actin gene (primers: TAA​CTG​TCT​GCT​TCT​CTG​CTG​TAG​GCC and GCT​TCA​CCA​CCT​TCT​TGA​TGT​CAT​CA). For RT-qPCR, total RNA of PFN2 expression was extracted from cells or PDX tissues using TRIzol Reagent (Invitrogen). Reverse transcription was performed using the High-Capacity cDNA Reverse Transcription Kit (Applied Biosystems). qPCR was performed using SYBR Green Master Mix (Bio-Rad) on a CFX96 Real-Time PCR System (Bio-Rad). GAPDH was used as the endogenous control. Each sample was run in triplicate, and each experiment was repeated three times. Primer specificity was validated by melting curve analysis and agarose gel electrophoresis.

### 2.11 RNA-seq experiment and data analysis

Total RNA was isolated from PDX No.1 tumors (VPA-treated vs. saline control, n = 3 mice per group) using TRIzol. After DNase I treatment, RNA quality was assessed by A_260_/A_280_ ratio and agarose gel electrophoresis. Library construction was performed using the KC-Digital™ Stranded mRNA Library Kit (Seqhealth) with 2 μg RNA, incorporating unique molecular identifiers (UMIs). Libraries (200–500 bp) were sequenced on a DNBSEQ-T7 platform (PE150).

Raw sequencing data were quality filtered with Trimmomatic (v0.36). Reads were aligned to a combined human GRCh38 (Ensembl v109) and mouse GRCm39 (Ensembl v109) reference genome using STAR v2.5.3a. For ambiguous cross-species mapped reads: only reads uniquely mapped to human chromosomes with MAPQ ≥10 were retained for human gene expression quantification; reads aligned to both human and mouse genomes or exclusively to mouse sequences were discarded to eliminate stromal mouse cell contamination. UMI-based deduplication was performed to remove PCR duplicates. Raw gene counts were obtained using featureCounts (Subread-1.5.1). Differential expression analysis was performed using edgeR v3.12.1 on raw count data (not RPKM). Normalization was performed using the TMM method. The design matrix included treatment group (VPA vs. control). Dispersion was estimated using the Cox-Reid profile-adjusted likelihood method with empirical Bayes shrinkage. For preliminary exploratory screening of candidate transcripts, we applied a loose pre-filtering cutoff of |log_2_FC| > 0.32 and unadjusted raw p < 0.05. We implemented the Benjamini–Hochberg (BH) multiple testing correction across all detected transcripts to generate adjusted FDR values as a *post hoc* statistical reference, following standard edgeR workflow recommendations. Transcripts passing the raw p/log_2_FC pre-filter but with BH-adjusted FDR ≥0.05 were defined as nominal exploratory candidate transcripts, which do not meet the formal statistical threshold for *bona fide* differentially expressed genes (DEGs). Only transcripts with both raw p < 0.05 and FDR <0.05 would be recognized as statistically validated DEGs. Functional enrichment of GO terms and KEGG pathways was performed using KOBAS v2.1.1 (Fisher’s exact test, *p* < 0.05). Given the exploratory nature of this analysis (one PDX donor, n = 3 per group), results are presented as hypothesis-generating.

### 2.12 Western blot

The Western blot method was done as previously reported (Chen et al., 2023). Briefly, protein samples (30 μg/10 μL) were first resolved by sodium dodecyl sulfate-polyacrylamide gel electrophoresis (SDS-PAGE) and then transferred onto a nitrocellulose membrane. The membrane was blocked with 5% non-fat milk in Tris-buffered saline containing 0.1% Tween 20 (TBST) to reduce non-specific binding. Subsequently, it was incubated with the primary antibody overnight at 4 °C, followed by horseradish peroxidase-conjugated secondary antibody for 1 h at room temperature. Protein bands were visualized using a chemiluminescent substrate and captured with an imaging system. Densitometric analysis of the bands was performed to quantify the protein expression levels. GAPDH (YM3027, Immunoway, 1:1,000) and β-Actin (YM3028, Immunoway, 1:1,000) used as the loading control. The primary antibodies were including PFN (YN2844, Immunoway, 1:1,000), PI3K (total p85α PI3K protein, YM8045, Immunoway, 1:1,000), p-PI3K (p85α Phospho Tyr607, YP0765, Immunoway, 1:1,000), AKT (YM3618, Immunoway, 1:1,000), p-AKT (Phospho Thr308, YP0590, Immunoway, 1:1,000). And the secondary antibodies were including anti-mouse (RS0092, Immunoway, 1:5,000) and anti-rabbit (RS0002, Immunoway, 1:5,000). Proteins were normalized to proteins GAPDH or β-actin for quantitative analysis. Each experiment was repeated three times independently.

### 2.13 Plasmid transfection

Three plasmid constructs were used in transfection assays: non-targeting negative control shRNA (NC-shRNA, shRNA sequences: 5′- TTC​TCC​GAA​CGT​GTC​ACG​T-3′, Genepharma A7597), PFN2-targeted shRNA (PFN2-KD, shRNA sequences: 5′- GCC​AAA​TAC​GTC​TGG​GCA​GCC-3′ Genepharma A7597), PFN2 overexpression plasmid (PFN2-OE, Genepharma custom vector). Namalwa suspension cells were seeded in 6-well plates at a fixed density of 2 × 10^5^ viable cells per well 24 h before transfection. Since Namalwa are suspension cells without adherent confluence metrics, pre-transfection viable cell density was strictly maintained at 2 × 10^5^ cells/well. Transfection was performed using Lipofectamine 3,000 (Invitrogen L3000015) following manufacturer’s protocol, plasmid: transfection reagent mass ratio = 1:2, 2 μg total plasmid per well. Cells were harvested 48 h post-transfection for downstream functional assays. Transfection efficiency was quantified via fluorescent reporter co-transfection, with an average transfection efficiency of 80% across three independent replicates. Cell viability was detected via CCK-8 at 48 h post-transfection, with no significant cytotoxicity induced by transfection reagents alone. Western blot quantification confirmed >70% PFN2 protein knockdown efficiency for shRNA compared with NC-shRNA groups. All transfection experiments were repeated for 3 independent biological replicates.

### 2.14 Data statistics

Data for each group are presented as the mean ± SEM. GraphPad Prism version 8.0 software (GraphPad Software, La Jolla, CA, United States) was utilized to analyze data and create graphs. Two-tailed unpaired Student’s t-test for two-group comparisons; one-way ANOVA with Tukey’s *post hoc* test for multiple groups; two-way ANOVA for longitudinal tumor volume data. Normality and homogeneity of variance assumptions were verified before analysis; Benjamini–Hochberg correction was applied for multiple comparisons. A p-value of less than 0.05 was considered to indicate a statistically significant difference between the groups.

## 3 Results

### 3.1 VPA inhibited the proliferation and induced apoptosis in BL cell lines

CCK-8 assay, which measures metabolic activity as an indicator of cell viability, showed that VPA inhibited BL cell viability in a time- and dose-dependent manner (Figure 1A). IC_50_ values at 24 h: Daudi 20.15 mM (95% CI: 14.61–31.89 mM), Raji 4.672 mM (95% CI: 2.798–6.826 mM), Namalwa 7.769 mM (95% CI: 5.821–11.25 mM). IC_50_ values at 48 h: Daudi 4.891 mM (95% CI: 3.641–6.316 mM), Raji 2.283 mM (95% CI: 1.674–2.856 mM), Namalwa 0.4836 mM (95% CI: 0.3600–0.5662 mM). Additionally, flow cytometry analysis of EdU-labeled proliferating cells revealed that the VPA (at concentrations of 2 or 4 mM) inhibited the proliferation of all the 3 cell lines after 24 h’ treatment (Daudi control 48.07 ± 0.318 vs. VPA 30.47 ± 0.433 *p* < 0.0001, Raji control 55.23 ± 3.023 vs. VPA 32.733 ± 5.478 *p* = 0.0228, Namalwa control 32.900 ± 0.700 vs. 1.383 ± 0.347 *p* < 0.0001) (Figure 1B).

**FIGURE 1:**
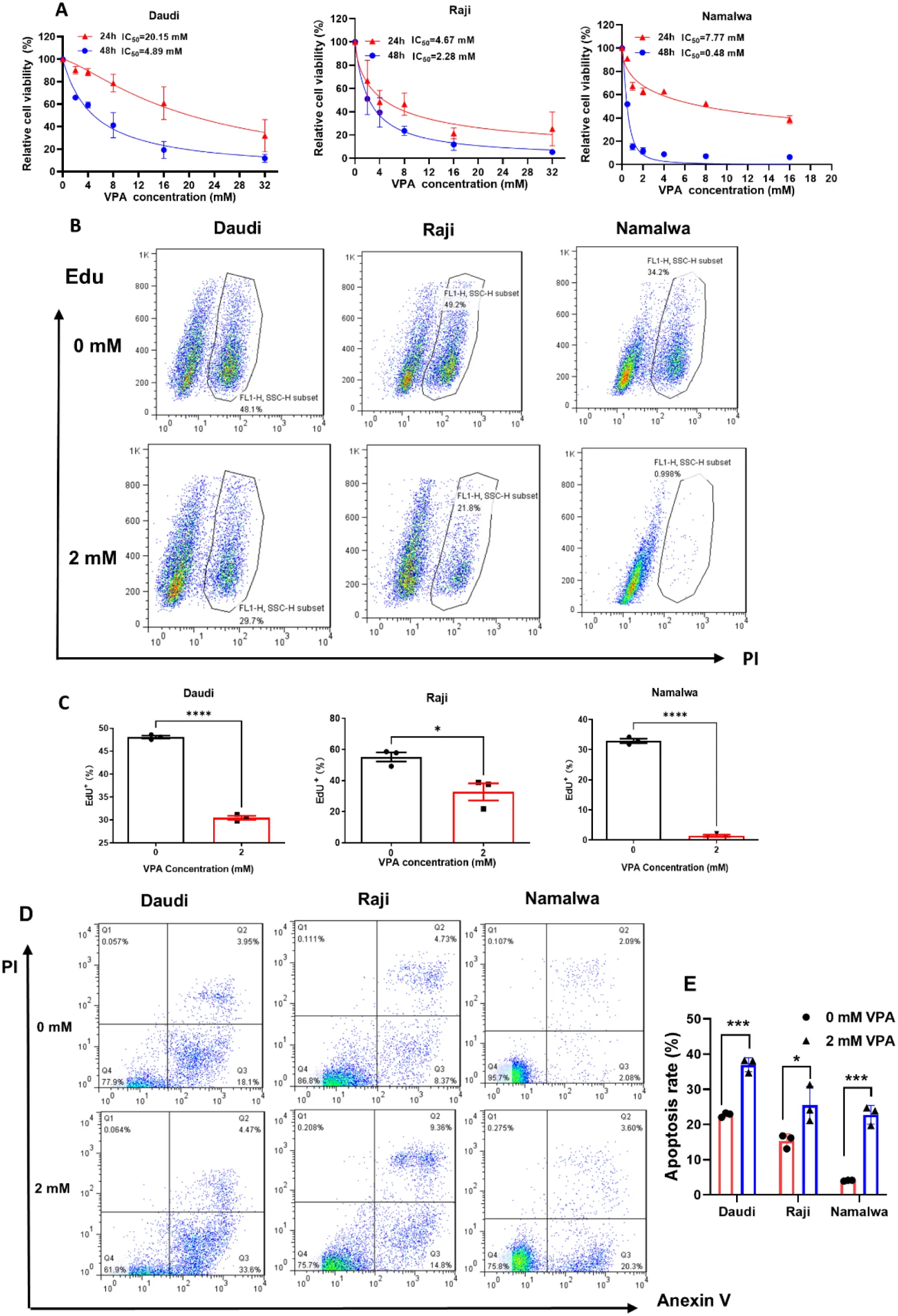
Proliferation of BL cell lines were inhibited by VPA. **(A)** The Raji, Daudi, Namalwa cell lines were treated with VPA with different concentrations and tested the proliferation by CCK-8 test. **(B,C)** After VPA treated, EdU-labeled proliferating cells were detected by flow cytometry. N = 3 independent experiments per group, FL1-H stand for PI, SSC-H stand for Edu. **(D,E)** VPA promotes apoptosis of Daudi, Raji and Namalwa cell line, the left panel is the flow cytometry of apoptosis rate, and the right panel is the statistical analysis. n = 3 independent experiment per group. *p < 0.05, ***p < 0.001, ****p < 0.0001.

Cell apoptosis was assessed using Annexin-V FITC/PI staining and detected by flow cytometry. After 24 h treatment with VPA (2 mM), total apoptosis rates (early + late) were: Daudi: control 22.78 ± 0.369 vs. VPA 37.00 ± 1.139 (*p* = 0.0003); Raji: control 15.35 ± 1.139 vs. VPA 25.55 ± 3.014 (*p* = 0.0341); Namalwa: control 4.10 ± 0.078 vs. VPA 22.78 ± 1.546 (*p* = 0.0003). VPA treatment for 24 h increased cell apoptosis in all 3 cell lines (Figures 1D,E).

### 3.2 VPA inhibited cell proliferation by arresting cell cycle in G1 phase

The cell-cycle distribution of Daudi, Raji and Namalwa BL cell lines was quantified via PI staining flow cytometry (full procedure detailed in Section 2.5). After 24 h treatment with gradient VPA (0.5, 1, 2 mM), quantitative analysis of DNA content histograms revealed a dose-dependent elevation in the proportion of cells residing in the G0/G1 phase, accompanied by a marked reduction in the percentage of S-phase cells; no consistent shift in the proportion of G2/M-phase cells was observed across all three BL cell lines (Figures 2A,B).

**FIGURE 2:**
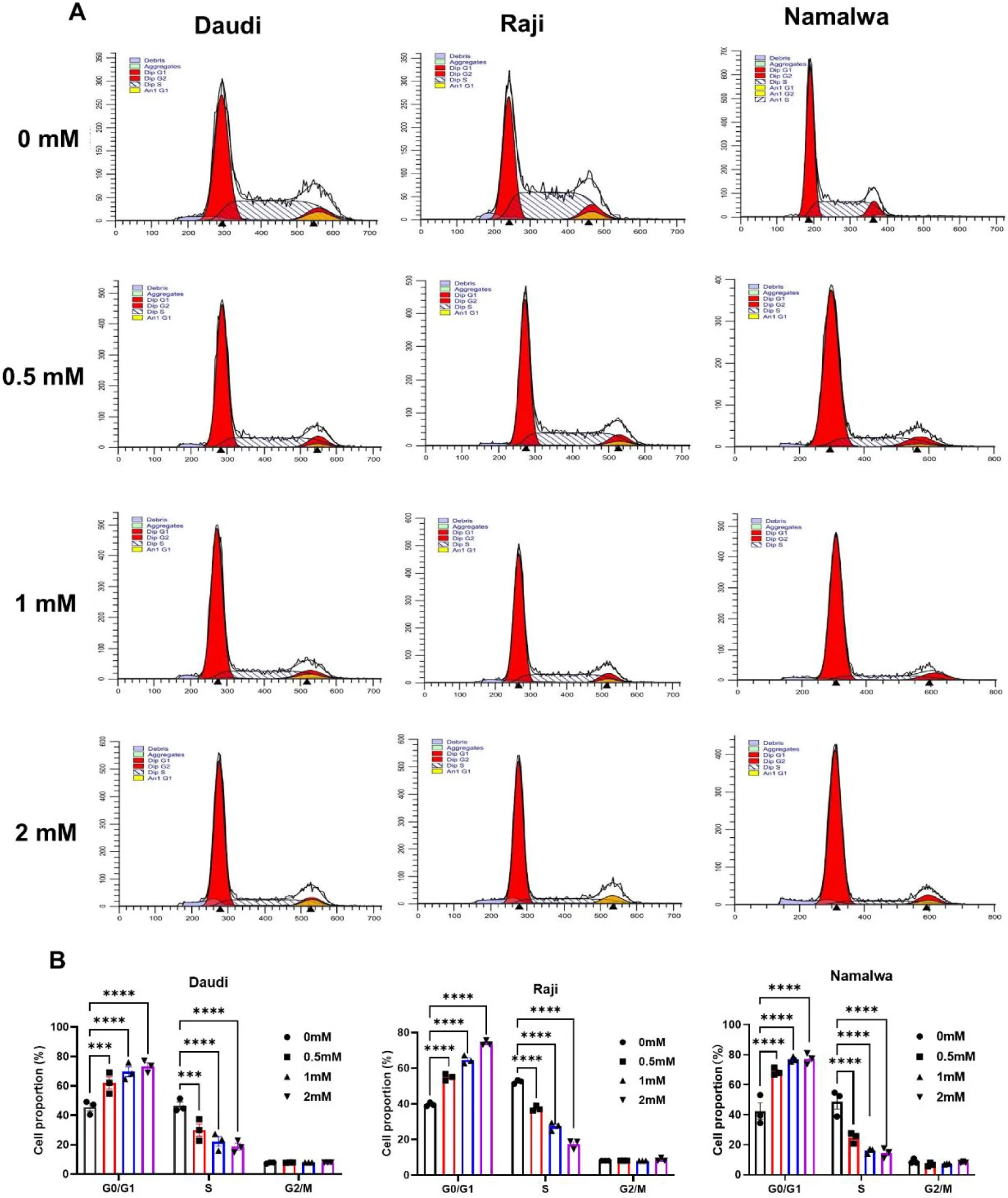
VPA induces G0/G1-phase cell-cycle accumulation in BL cell lines after 24 h VPA exposure. **(A)** Representative DNA content histograms showing cell-cycle distribution of Daudi, Raji and Namalwa cells treated with gradient VPA (0, 0.5, 1, 2 mM) for 24 h. **(B)** Quantified bar charts presenting the mean percentage of cells distributed in G0/G1, S, and G2/M phases for each experimental group. N = 3 independent biological replicates per group, ***p < 0.001, ****p < 0.0001.

### 3.3 VPA inhibited the growth of namalwa BL CDX model

To further validate the inhibitory effect of VPA on BL, we used the BL Namalwa CDX model to investigate the inhibitory effect of VPA *in vivo*. Results showed that, compared to the control group (n = 5), tumor volume and final tumor weight (control 1.132 ± 0.13 vs. VPA 0.365 ± 0.121, *p* = 0.0207,95% Cl −0.8982 to −0.09539) were reduced in the VPA treatment group (n = 6, 400 mg/kg, i. p., once daily for 1 week), while the mouse’s weight of VPA treated mice did not show a decrease (Figures 3A–C). 1 mice was excluded from Namalwa CDX model.

**FIGURE 3:**
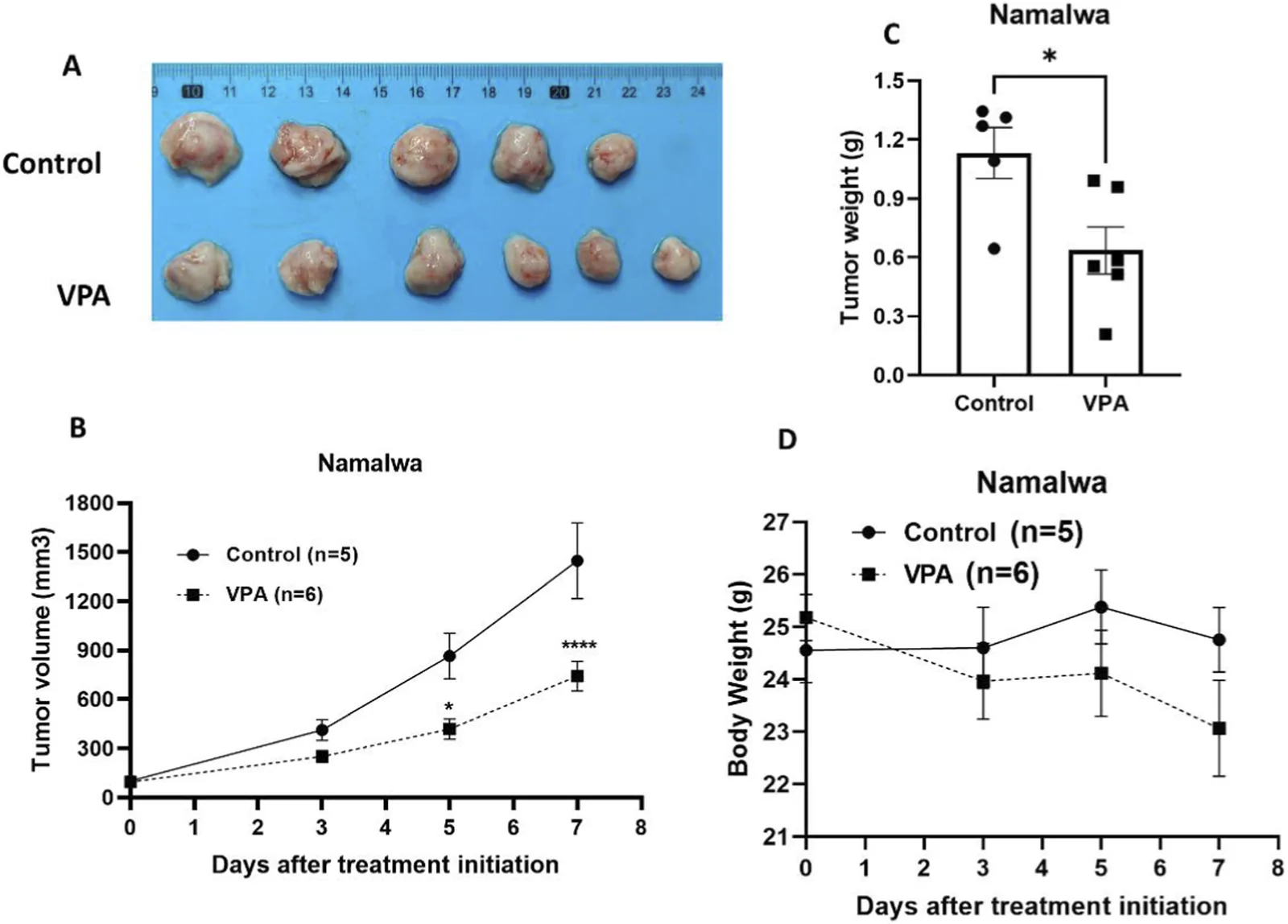
VPA inhibited Namalwa BL CDX growth. **(A)** General and tumor photograph of CDX models. **(B,C)** Statistical data showed that VPA inhibited the tumor volume, tumor weight and mouse weight *in vivo*, n = 5 in control group, n = 6 in VPA group, *p < 0.05, ****p < 0.0001. **(D)** The body weight of mice treated with VPA were not affected.

### 3.4 Construction of patient-derived BL xenografts

The patient-derived tumor xenograft (PDX) model is increasingly used in translational cancer research. PDX models partially recapitulate intrinsic molecular and histopathological features of matched patient BL lesions, but standard subcutaneous PDX established in NOD-SCID immunodeficient mice fail to preserve intact human immune compartments and native patient-derived tumor stroma (Abdolahi et al., 2022). These models are useful for drug screening, biomarker development, and the preclinical evaluation of personalized medicine strategies (Hidalgo et al., 2014). In order to further confirm the therapeutic effect of VPA in clinical patients, we successfully established three cases of BL PDX models, and verified histological and protein expression consistency between the PDX models and the patients’ tumors (Figures 4A,B). H&E staining of PDX tumor tissue displayed identical histological features to matched patient primary tumors, including monomorphic B-cell proliferation and starry sky morphology (Figure 4A). IHC staining showed consistent positive expression of MYC, BCL6 and Ki67 between patient specimens and corresponding PDX passages (Figure 4B). Human-specific GAPDH/β-actin PCR bands were detectable in all three PDX tumor samples, with no mouse-only amplification products observed (Figure 4C).

**FIGURE 4:**
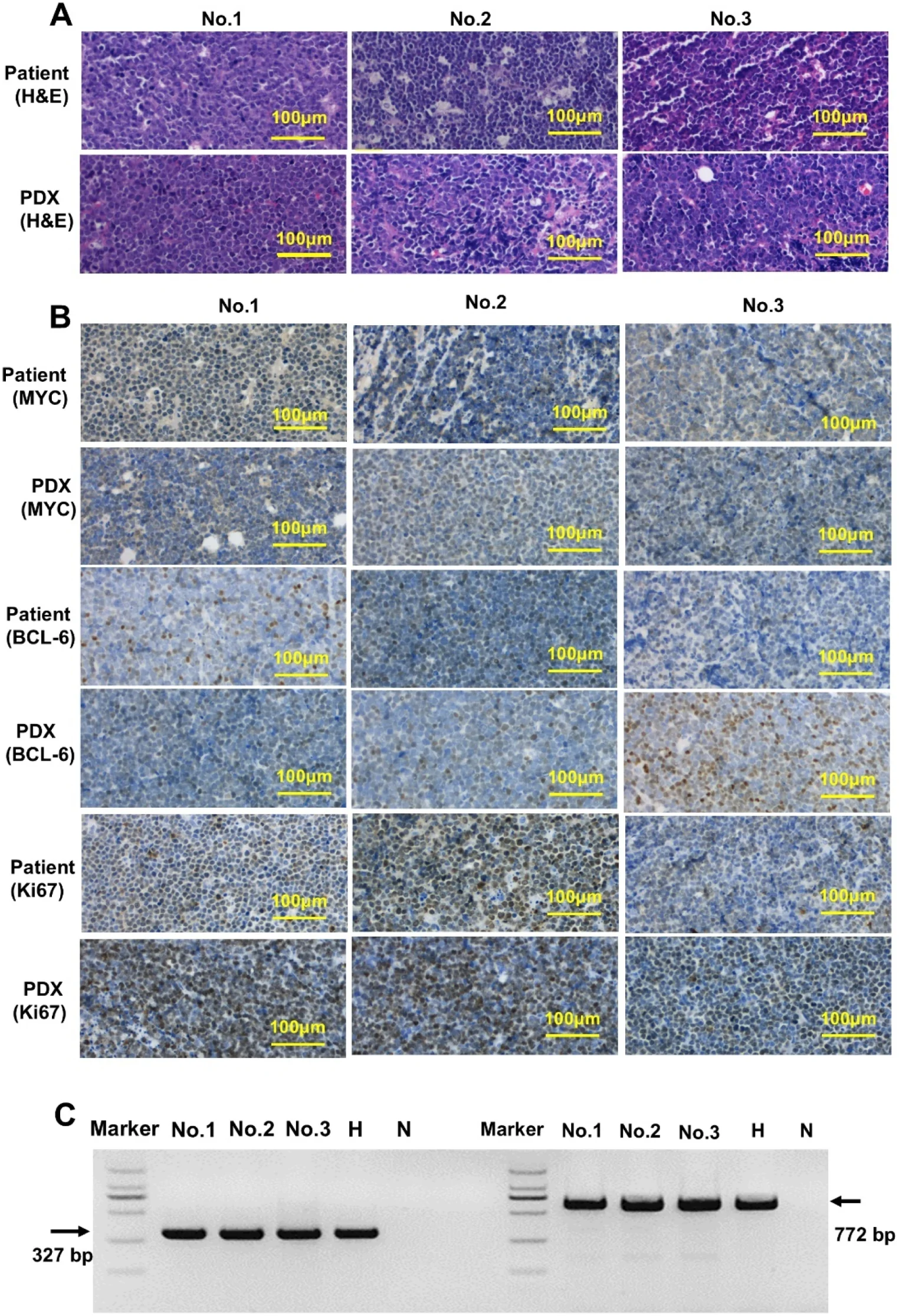
BL PDX models were consistent with tumor tissue of patients. **(A)** PDX models retained the principal histologic by H&E staining, n = 6 independent experiments per group. **(B)** IHC results showed the protein expression of MYC, BCL-6 and Ki67 in PDX and tumor tissue of patients was positive, n = 6 per group. **(C)** PDX models were verified with human gene by PCR, H means positive control, N means negative control.

### 3.5 VPA inhibited the activity of patient-derived tumor tissues *in vitro* and *in vivo*

Hydrogel embedded histoculture drug sensitivity test (HDST) is a new drug sensitivity detection method, which uses patient-derived tumor tissue or PDX tumor tissue block to detect drug sensitivity *in vitro*. This approach has a low cost, short time required (5–7 days), and facilitates high-throughput drug screening (Yuanqiao et al., 2023). We used tumor tissue of BL PDX model and tested the inhibitory effect of VPA by HDST method. Results showed that VPA could inhibit patient-derived BL in a time - and dose-dependent manner (IC_50_ for 5 days: No.1 = 2.15 mM, 95% CI = 1.947–2.368, No.2 = 2.18 mM, 95% CI = 1.708–2.802, No.3 = 0.57 mM, 95% CI = 0.4146–0.7794; IC_50_ for 7 days: No.1 = 2.12 mM, 95% CI = 1.880–2.377, No.2 = 0.41 mM, 95% CI = 0.2299–0.5920, No.3 = 0.12 mM, 95% CI = 0.0746–0.1821) (Figures 5A–C).

**FIGURE 5:**
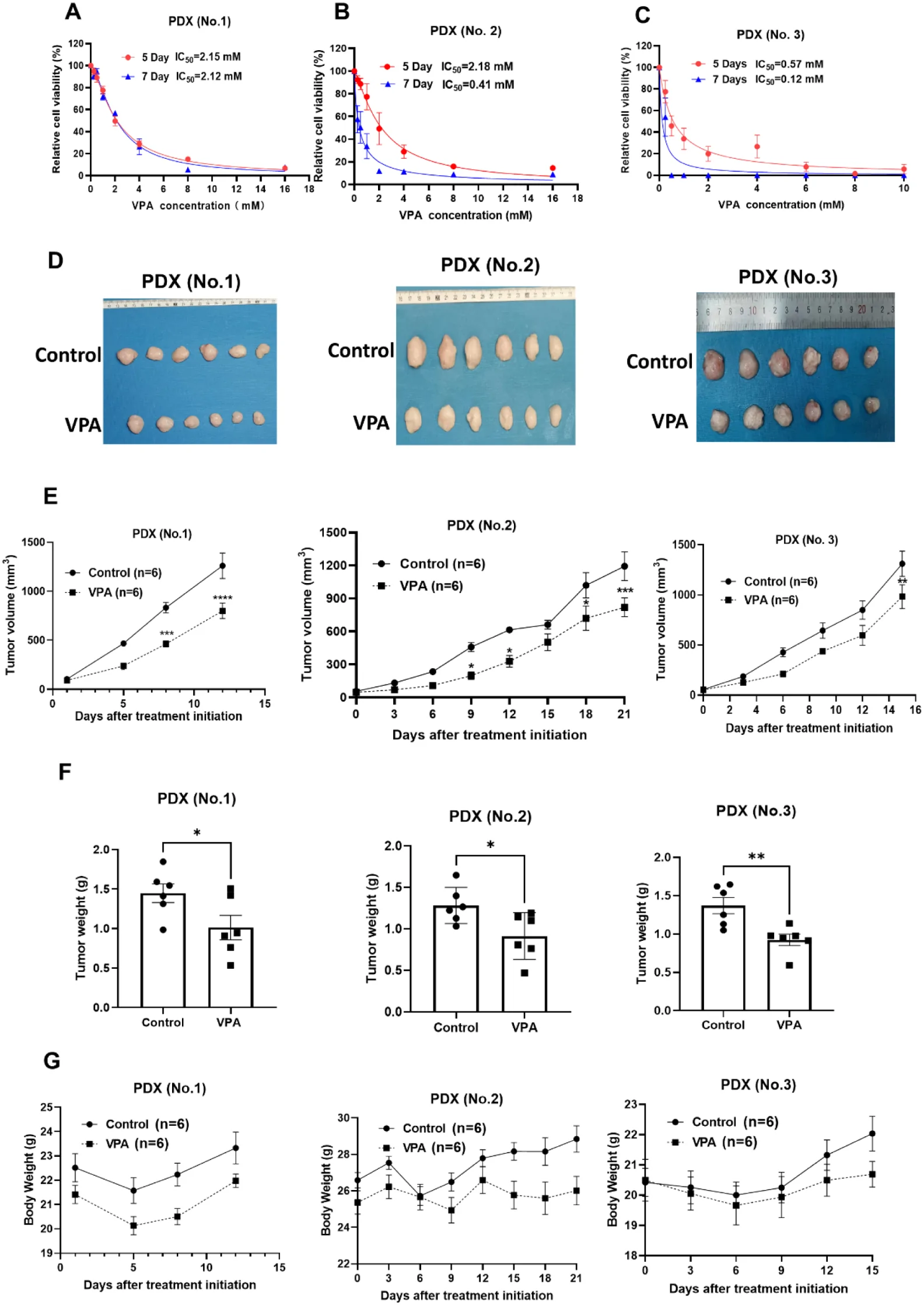
VPA inhibited BL PDX tumor tissues *in vitro and vivo*. **(A–C)** The inhibition rate of VPA treated PDX was detected by HDST. **(D)** Tumor photograph of PDX was treated by VPA. **(E–G)** Statistical data showed that VPA inhibited the tumor volume, tumor weight and the mouse weight of BL PDX models, *p < 0.05, **p < 0.01, ***p < 0.001, ****p < 0.0001.

Furthermore, *In vivo* efficacy testing is conducted separately for each PDX donor model, with six mice assigned to the control group and six mice assigned to the VPA treatment group for PDX No.1, No.2, and No.3. Baseline tumor volumes ranging from 30 to 150 mm^3^ are evenly stratified prior to random group allocation (Baseline tumor volume: PDX No.1 control 102.8 ± 6.197, VPA 90.935 ± 4.290; PDX No.2 control 55.508 ± 4.033, VPA 46.795 ± 4.618; PDX No.3 control 56.493 ± 4.112, VPA 52.797 ± 3.549). Longitudinal tumor volume measurements are acquired every 2–3 days throughout the 1–3 weeks treatment course for each individual PDX line. Relative to the saline control cohort, VPA administration yields suppression of tumor progression and decreased terminal tumor weight in PDX No.1, PDX No.2, and PDX No.3 (No.1 control 1.450 ± 0.119 g vs. VPA 1.013 ± 0.155 g, *p* = 0.0490, 95% Cl −0.8712 to −0.0023; No.2 control 1.283 ± 0.089 g vs. VPA 0.913 ± 0.115 g, *p* = 0.0295, 95% Cl −0.6940 to −0.0451; No.3 control 1.371 ± 0.107 g vs. VPA 0.924 ± 0.074 g, *p* = 0.0062, 95% Cl −0.7364 to −0.1588) (Figures 5D–F). Synchronous body weight monitoring is implemented to assess treatment safety. The mouse’s weight of VPA treated mice did not show a decrease (two-way ANOVA, PDX No.1: F (3,40) = 0.155, *p* = 0.9256; PDX No.2: F (7,80) = 0.9876, *p* = 0.4463; PDX No.3: F (5,60) = 0.426, *p* = 0.8282) (Figure 5G). 2 mice were excluded from PDX No.1, 0 from PDX No.2, and 0 from PDX No.3 before randomization, all due to out-of-range baseline tumor volume. No obvious lethargy, fur loss, drastic weight loss or unexpected death was observed in any PDX group during the whole treatment course.

### 3.6 VPA treatment induces transcriptional downregulation of PFN2 in BL

In order to explore the possible mechanism by which VPA inhibited BL growth, PDX model (No.1) tumor tissue after VPA treatment *in vivo* was used for the transcriptomic analyses (RNA-seq analysis). RNA-seq exploratory transcriptomic analysis was performed on PDX No.1 tumor tissues (VPA vs. saline control, n = 3 biological replicates per group). After pre-filtering by |log_2_FC| > 0.32 and raw p < 0.05, a total of 340 nominal exploratory candidate transcripts were retained, including 189 transcripts with nominally increased expression and 151 transcripts with nominally decreased expression (Figure 6A). Among these nominal candidates, PFN2 exhibited moderate nominal transcriptional reduction, with log_2_FC = −0.3213, raw p = 0.0355, and BH-adjusted FDR = 0.6149 (Figure 6B). Notably, FDR >0.05 indicated that PFN2 did not reach the statistical threshold for validated differentially expressed genes, and we therefore designated PFN2 as an exploratory candidate gene with nominal downregulation rather than a statistically confirmed DEG. According to GO analysis, VPA affected intracellular, intracellular part, cell, cellular process, etc (Figure 6C). KEGG analysis found that PI3K-AKT signaling pathway was enriched after VPA treatment (Figure 6D).

**FIGURE 6:**
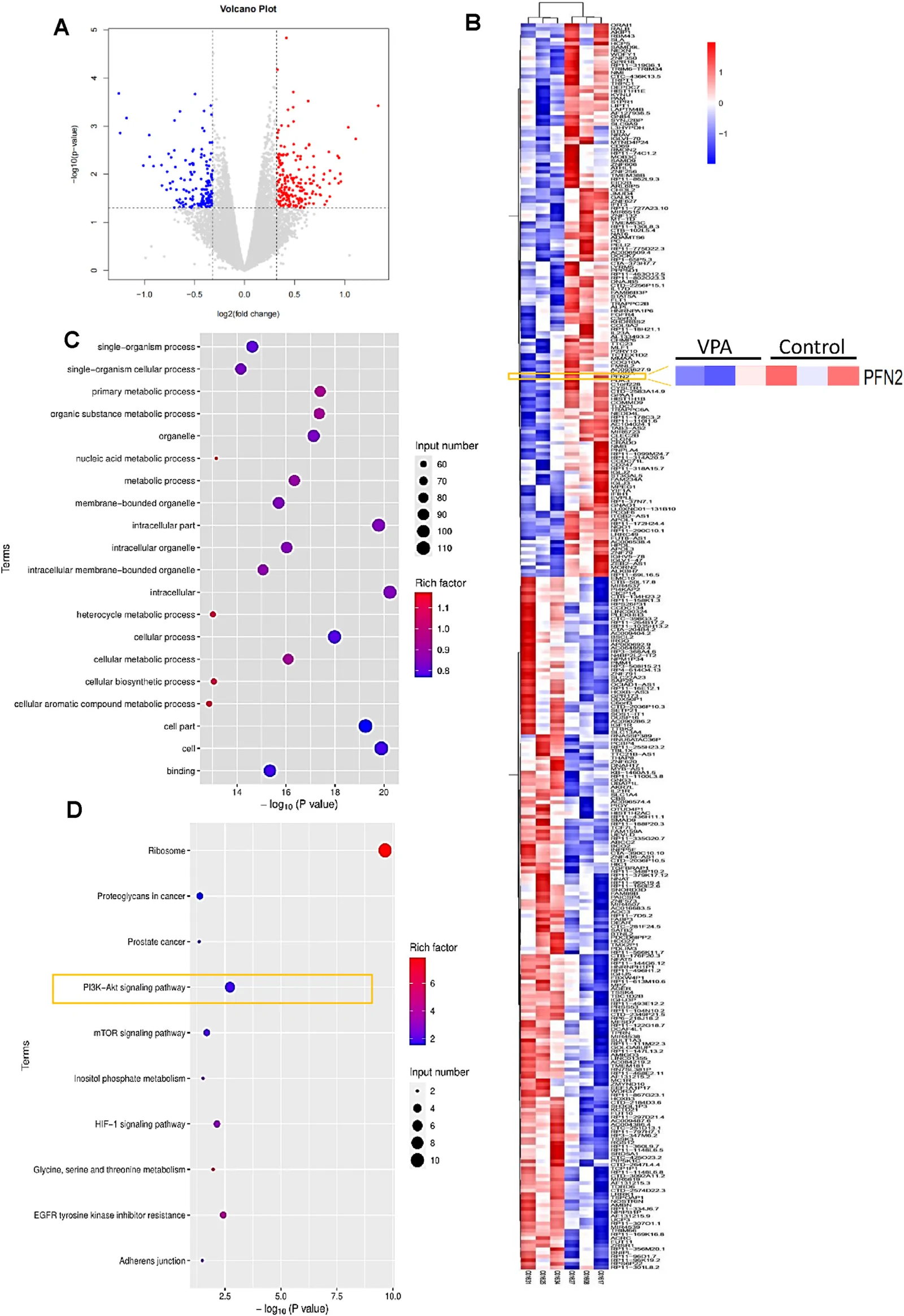
RNA-Seq Analysis Revealed Genes and Pathways involved in VPA treatment. **(A)** Volcano plot of differential expressed genes in VPA treatment group. Upregulated exploratory candidate gene are labeled as red dots, whereas downregulated exploratory candidate gene are labeled in blue. **(B)** Heatmap of genes after VPA treatment. **(C)** Go enrichment. **(D)** KEGG pathway.

To further verify the effect of VPA on the expression of PFN2 as an exploratory candidate gene, we verified the expression of PFN2 gene in BL PDX (No.1) and cell lines (Daudi, Raji and Namalwa) by q-PCR and WB. Results showed that the mRNA expression of PFN2 gene was reduced in VPA treated PDX (No.1) tumor tissue (t-test, *p* = 0.0041). And in BL cell lines (Daudi, Raji, and Namalwa), the mRNA expression of PFN2 gene was reduced in a dose-dependent manner (Figures 7B–D). In VPA treated BL PDX (No.1) and Namalwa cell line, the protein expression of PFN2 was also reduced (PDX No.1: control vs. VPA *p* = 0.0274, t-test; Namalwa: 0 mM vs. 0.5 mM *p* = 0.0334, 0 mM vs. 1 mM *p* = 0.0219, 0 mM vs. 2 mM *p* = 0.0057, one-way ANOVA) (Figures 7E,F).

**FIGURE 7:**
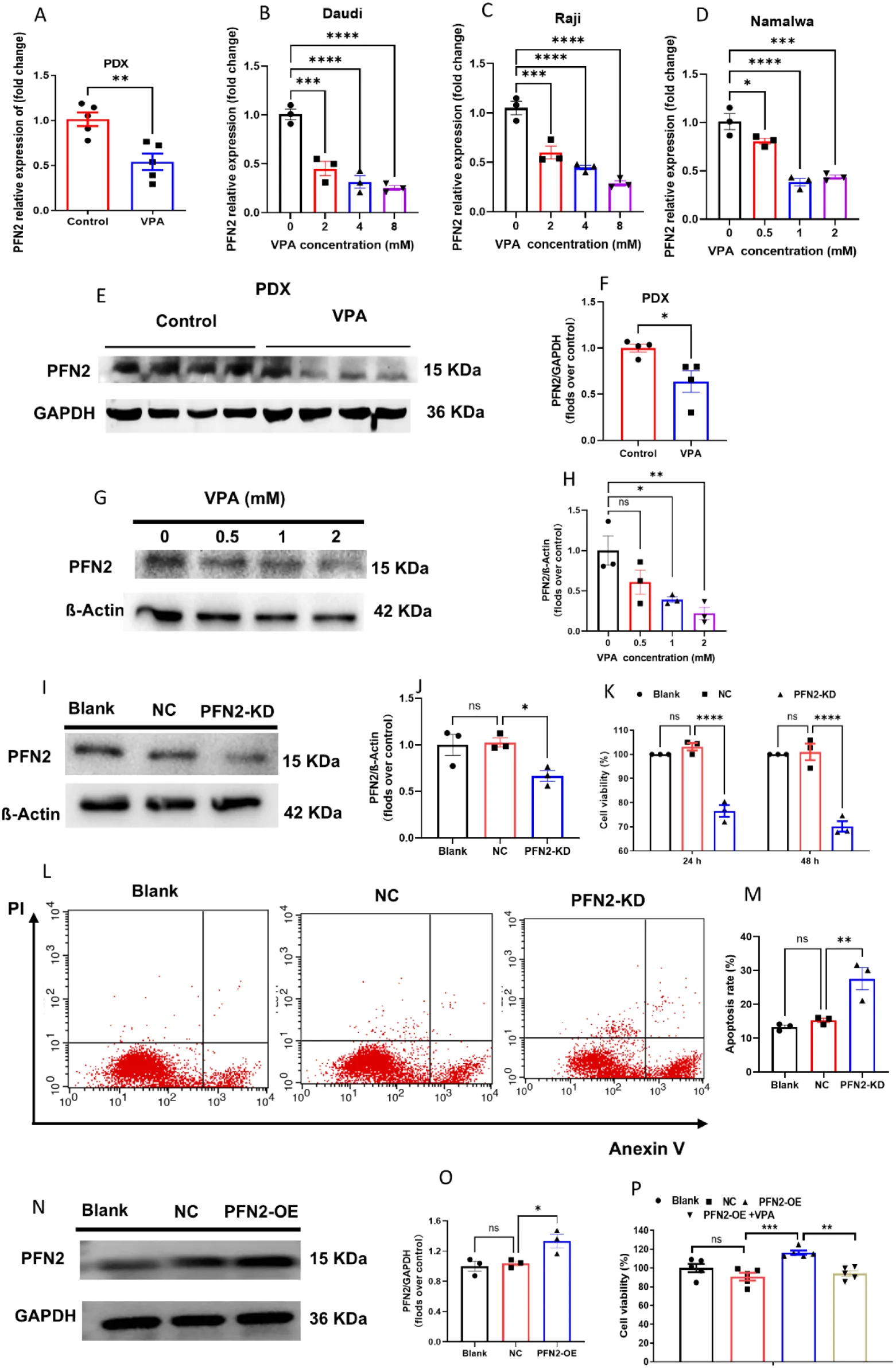
VPA reduced PFN2 gene expression in BL. **(A)** The expression of PFN2 gene was reduced in BL PDX (No.1) treated by VPA (400 mg/kg) by q-PCR test, n = 5 per group, **p < 0.01. **(B–D)** The expression of PFN2 was reduced in Daudi, Raji, and Namalwa cell lines treated by VPA by q-PCR test, n = 3 per group, *p < 0.05, ***p < 0.001, ****p < 0.0001. **(E,F)** The expression of PFN2 protein was reduced in BL PDX (No.1) treated by VPA (400 mg/kg), n = 4 per group, *p < 0.05. **(G,H)** The expression of PFN2 protein was reduced in Namalwa cell lines treated by VPA, n = 3 per group, *p < 0.05, **p < 0.01. **(I,J)** The expression of PFN2 protein was reduced after the PFN2 knockdown. **(K)** The proliferation of Namalwa cell line was decreased after the PFN2 knockdown, NC vector was the no-load plasmid, PFN2-KD inhibited the expression of PFN2, n = 3 independent experiments per group, ns > 0.05, *p < 0.05, ****p < 0.0001. **(L,M)** The apoptosis rate was tested by flow cytometry, n = 3 per group, ns > 0.05, **p < 0.01. **(N,O)** PFN2 protein was over expression in Namalwa cell line, NC vector was the no-load plasmid, PFN2-OE was mead the over expression of PFN2, ns > 0.05, *p < 0.05. **(P)** VPA inhibited PFN2 over expression induced cell proliferation, ns > 0.05, **p < 0.01, ***p < 0.001.

Finally, we transfected PFN2 vector (which inhibited the expression of PFN2, shRNA) and NC vector (no-load plasmid) respectively into Namalwa cell line. The knockdown efficiency was verified by WB (one-way ANOVA, Blank vs. NC *p* = 0.9583, NC vs. PFN2-KD *p* = 0.0319) (Figures 7G–I). We found that the proliferation of BL cell line (Namalwa) was decreased after PFN2 knocked down for 24 h and 48 h (two-way ANOVA, 24 h: Blank vs. NC *p* = 0.4706, NC vs. PFN2-KD *p* < 0.0001; 48 h: Blank vs. NC *p* = 0.9244, NC vs. PFN2-KD *p* < 0.0001) (Figure 8K). In addition, apoptosis of Namalwa cell line was increased after PFN2 downregulation (one-way ANOVA, Blank vs. NC *p* = 0.7085, NC vs. PFN2-KD *p* = 0.0077) (Figures 7L,M). Transfected Namalwa cells with a PFN2 overexpression plasmid (PFN2-OE) or an empty vector control or PFN2-OE added VPA treatment for 48 h to test the CCK8 cell proliferation assay. The experimental results showed that overexpression of PFN2 promoted the proliferation of Namalwa Burkitt lymphoma cells, and VPA (2 mM) could inhibit the cell proliferation caused by PFN2 overexpression (two-way ANOVA, Blank vs. NC *p* = 0.2222, NC vs. PFN2-OE *p* = 0.0003, PFN2-OE vs. PFN2-OE + VPA *p* = 0.0012) (Figures 7N–P). No significant difference in cell viability between the NC + VPA and Control + VPA groups (the concentration of VPA is 2 mM) (Control + VPA 14.053 ± 2.143 vs. NC + VPA 12.183 ± 3.130, *p* = 0.6384) (Supplementary 1).

**FIGURE 8:**
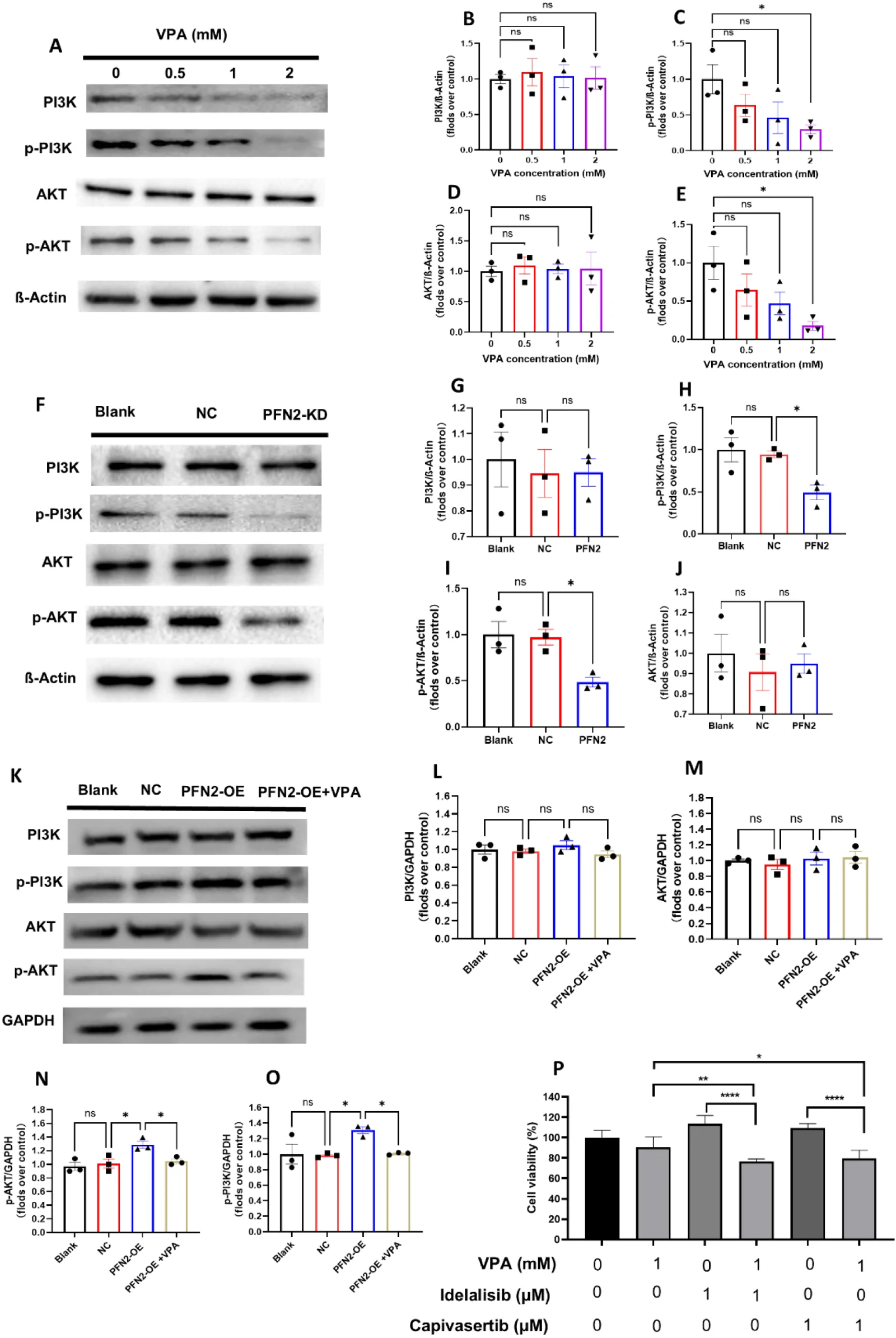
The expression levels of PI3K/AKT signaling pathways in VPA treated Namalwa cell line and PFN2 knocked down or over expressed in Namalwa cell line. **(A)** Western blotting detected the protein expression levels of PI3K, p-PI3K, AKT, p-AKT in VPA treated Namalwa cell line. **(B–E)** The protein expression of PI3K, p-PI3K, AKT, p-AKT was quantified in VPA treated Namalwa cell line, n = 3 independent experiments per group, ns > 0.05, *p < 0.05, **p < 0.01. **(F)** Western blotting detected the protein expression levels of PI3K, p-PI3K, AKT and p-AKT when PFN2 knocked down in Namalwa cell line. **(G–J)** The protein expression of PI3K, p-PI3K, AKT and p-AKT was quantified when PFN2 knocked down in Namalwa cell line, n = 3 independent experiments per group, ns > 0.05, *p < 0.05. **(K–O)** Western blotting detected the protein expression levels of PI3K, p-PI3K, AKT and p-AKT when PFN2 over expressed in Namalwa cell line. **(P)** The combination of VPA with PI3K/AKT inhibitors enhances the inhibitory effect of VPA on the Namalwa cell line, Idelalisib was PI3K inhibitor, Capivasertib was AKT inhibitor.

### 3.7 VPA exposure correlates with PFN2 downregulation and subsequent attenuation of PI3K/AKT signaling cascades

We verified whether VPA inhibited the growth of BL through the PI3K-AKT signaling pathway. Protein expression in Namalwa cell lines treated with VPA (0.5, 1, 2 mM) was detected by WB. The results showed that in the VPA treated group, not only the expression of PFN2 was decreased, but also the expression levels of p-PI3K and p-AKT were decreased, while the expression of PI3K and AKT remained unchanged (Figures 8A–E). Similarly, after PFN knockdown, the expression of p-PI3K and p-AKT was also decreased, and there was no difference in the expression of PI3K and AKT (Figures 8F–J). PFN2 overexpression increased p-PI3K and p-AKT protein levels, and VPA (2 mM) was attenuated the PFN2 overexpression induced increasing in p-PI3K and p-AKT levels (Figures 8K–O).

CCK8 cell proliferation assay was conducted to evaluate the effect of VPA combined with the inhibitors of PI3K (Idelalisib, 1 μM) and AKT (Capivasertib, 1 μM). The results showed that the combination of VPA with idelalisib or capivasertib enhanced the inhibitory effect of VPA on Namalwa Burkitt lymphocytes (VPA 90.859 ± 3.964 vs. VPA + idelalisib 76.422 ± 1.061, *p* = 0.0055; idelalisib 113.813 ± 3.125 vs. VPA + idelalisib 76.422 ± 1.061, *p* < 0.0001; capivasertib 109.208 ± 1.854 vs. VPA + capivasertib 79.381 ± 3.36, *p* < 0.0001; VPA 90.859 ± 3.964 vs. VPA + capivasertib 79.381 ± 3.36, *p* = 0.0345) (Figure 8P).

## 4 Discussion

Although intensive multiagent chemotherapy regimens cure a substantial proportion of BL patients, outcomes remain poor for older adults (≥60 years), patients with relapsed or refractory disease, and those unable to tolerate intensive therapy due to comorbidities or toxicity. These populations represent an unmet clinical need. In this study, we found that valproic acid has a good inhibitory effect on BL. VPA is clinically approved for the treatment of epilepsy, manic episodes associated with bipolar disorder, and migraine prophylaxis (Koch-Wese et al., 1980; Linde et al., 2013; Rana et al., 2005; Pinder et al., 1977). As a an anticonvulsant and mood-stabilizing drug, the safety of VPA has been extensively validated through clinical trials. VPA systemic toxicities (hepatotoxicity, hematologic suppression, teratogenicity) as critical translational considerations for future animal toxicity testing. The mechanism of action involves modulation of gamma-aminobutyric acid (GABA) function and inhibition of voltage-gated sodium channels, contributing to its therapeutic effects (Linde et al., 2013).

We found that VPA can inhibit BL cell proliferation, cell cycle and promote apoptosis, and we constructed CDX and PDX models to confirm its anti-BL effect *in vivo*. We constructed three BL PDX models, which partially recapitulate the tumor characteristics of their corresponding donor patients and provide a preliminary preclinical platform to evaluate VPA activity across a small subset of BL cases. HDST technology is a valuable complement to the PDX model, offering a fast, convenient, and cost-effective of anti-cancer drugs *in vitro*. We observed that VPA exerted significant anti-BL activity in both established cell lines and CDX models. Additionally, VPA exhibited potent anti-BL effects *in vivo*, as evidenced by experiments conducted using a patient-derived xenograft (PDX) model of BL. Our *in vitro* cell, CDX, PDX and *ex vivo* HDST assays collectively demonstrate anti-tumor activity of VPA against EBV-positive BL under preclinical experimental conditions. However, these preclinical observations cannot be directly extrapolated to human clinical outcomes, given inherent limitations of immunodeficient xenograft and *ex vivo* culture systems. The 400 mg/kg VPA dose used in mice is commonly applied in preclinical research (Pragnya et al., 2014), with a calculated human equivalent dose within the clinically relevant range (Drott et al., 2018; Rocca et al., 2009). Notably, the millimolar concentrations used for cell culture differ from clinical achievable VPA concentrations. Three distinct valproic acid (VPA) exposure compartments are defined based on clinical pharmacology literature: total plasma concentration, pharmacologically active unbound fraction, and intratumoral tissue concentration. Standard therapeutic total VPA concentrations for epilepsy range from 0.35 to 0.69 mM (50–100 μg/mL), while dose-escalated regimens for anti-tumor purposes target 0.69–1.04 mM (100–150 μg/mL); total plasma concentrations exceeding 1.04 mM are associated with severe organ toxicities (Su et al., 2011). Approximately 85%–95% of circulating VPA binds to serum albumin, leaving only 5%–15% as biologically active free drug (Kanner, 2003). Hypoalbuminemia commonly observed in advanced lymphoma patients elevates free VPA ratios and toxicity without improving anti-tumor potency. Tumor interstitial VPA concentrations have not yet been reported. *In vitro* half-maximal inhibitory concentrations (IC_50_) across all BL cell lines span 0.48–20.15 mM, values markedly higher than systemic and intratumoral VPA concentrations achievable in human subjects. No intratumoral VPA quantification is conducted in CDX and PDX xenograft models, and allometric conversion of the 400 mg/kg murine dose fails to accurately recapitulate local drug penetration within human lymphoma lesions. Safety profiles established from low-dose neurological administration cannot be extrapolated to high-dose anti-lymphoma schedules. Phase I clinical trials simultaneously measuring total plasma, free unbound, and intratumoral VPA concentrations are necessary to establish a safe therapeutic window for BL treatment. In other cancer research, it was found that VPA regulated MIEF1 through MST2-HIPPO to suppress breast cancer growth (Du et al., 2022), and valproic acid inhibited lipogenesis and induced apoptosis in prostate cancer cells via the C/EBPα/SREBP-1 pathway. These reports are consistent with the results of our study.

VPA can inhibit neuronal electrical signals by inhibiting sodium, potassium and calcium ion channels in the central nervous system (Rahman et al., 2024). Peripherally, it can be involved in the regulation of cell survival, transcriptional regulation, ion homeostasis, signal transduction and cytoskeletal modification (Rahman et al., 2024). We discovered that VPA-induced BL growth inhibition is associated with PFN2 downregulation and PI3K/AKT pathway attenuation. Notably, the observed reduction in PFN2 expression after VPA treatment only establishes a correlative transcriptional change rather than confirming direct pharmacologic targeting of PFN2 by VPA. RNA-seq only detected a mild transcriptional reduction of PFN2 (log_2_FC = −0.3213) after VPA treatment, yet Western blot showed much more obvious suppression of PFN2 protein abundance. This inconsistency can be reasonably explained: VPA may repress PFN2 not only at the transcriptional level but also through post-transcriptional or post-translational mechanisms to drastically cut functional PFN2 protein pools. As a dosage-sensitive scaffold protein governing PI3K/AKT signaling, subtle drops in PFN2 expression are sufficient to weaken downstream oncogenic cascades without requiring dramatic mRNA shifts. Moreover, VPA acts as a broad-spectrum HDAC inhibitor that remodels global chromatin and alters hundreds of transcripts simultaneously (Dowdell et al., 2009), so moderate fold changes of individual genes like PFN2 are expected within the composite anti-tumor transcriptional signature. Follow-up epigenetic profiling such as ChIP-seq will help further clarify how VPA subtly tunes PFN2 transcription via histone modification. Through “THE HUMAN PROTEIN ATLAS” web query, it was found that the expression of PFN2 in BL Namalwa was more than Raji and Daudi. Through cell proliferation experiments. It has been reported that PFNs regulates tumor growth through the AKT pathway (Das et al., 2009). In recent years, there has been increasing evidence highlighting the critical role of PFN2 in various cancers (Tang et al., 2015). PFN2 promotes proliferation and metastasis of head and neck cancer cells by up-regulating the PI3K/Akt/β-catenin signaling pathway (Zhou et al., 2019). In our study, we found that p-PI3K and p-AKT proteins were decreased upon VPA treatment. Reduced p-PI3K and p-AKT levels demonstrate an association with reduced pathway signaling. It remains possible that these changes are secondary to reduced cell viability or apoptosis rather than a direct cause of the anti-tumor effect. Temporal studies examining phosphorylation changes before the onset of apoptosis would be needed to establish causation. The PI3K/AKT signaling pathway is involved in the regulation of various cellular biological processes, such as proliferation, cell cycle, differentiation, metabolism, migration, and angiogenesis, and is often overactivated in many cancers (Porta et al., 2014; Peng et al., 2022; Noorolyai et al., 2019). The most widely studied of the PI3K family is type I PI3K, which is a dimeric protein composed of catalytic subunit p110 and regulatory subunit p85 (Ward and Finan, 2003). In cancer cells, the overactivation of AKT triggers phosphorylation of substrates, which determines whether its activity is blocked or enhanced (Tsai et al., 2022). BL In addition, studies have shown that the PI3K/AKT signaling pathway can inhibit apoptosis by activating BCL2 and inactivating BAX (Henshall et al., 2002; Xin and Deng, 2005).

It was reported that valproic acid can inhibit PI3K/AKT signaling by activating PTEN in Burkitt lymphoma cell (Li et al., 2025). They jointly explored the inhibitory effect of valproic acid on Burkitt lymphoma from different pathways, revealed the multiple targets of valproic acid on Burkitt lymphoma, and enriched the understanding of the anti-BL mechanism of VPA. Moreover, our study confirmed that valproic acid not only has inhibitory effect on Burkitt cell lines, but also has significant inhibitory effect on *in vivo* Burkitt lymphoma CDX and PDX models. Notably, all three BL cell lines (Daudi, Raji, Namalwa) and three patient-derived PDX models applied throughout this study carry detectable EBV expression, representing a uniform EBV-positive experimental system. Given the exclusive use of EBV-positive models here, the anti-tumor effects mediated via the PFN2/PI3K/AKT axis may be partially shaped by EBV-dependent intracellular signaling networks; it remains unclear whether identical PFN2 downregulation and pathway suppression would be triggered by VPA within EBV-negative BL subtypes. This uniform EBV positivity imposes a limitation on the broad generalizability of the present mechanistic conclusions across the full spectrum of BL biology, which encompasses both EBV-positive and EBV-negative disease subgroups. Future work incorporating EBV-negative BL cell lines and patient-derived models will be required to dissect whether EBV status modulates cellular sensitivity to VPA and validate the conserved function of the PFN2/PI3K/AKT regulatory axis independent of viral infection.

VPA-induced downregulation of PFN2 is consistently observed across BL cell lines and PDX tumor tissue, yet the upstream molecular cascade driving this transcriptional suppression remains uncharacterized in the present study. As a known HDAC inhibitory agent, VPA may repress PFN2 transcription via epigenetic remodeling of the PFN gene locus. Other indirect signaling cascades perturbed by VPA may also converge to negatively regulate PFN2 expression at the transcriptional or post-transcriptional level. No orthogonal experiments such as chromatin immunoprecipitation sequencing (ChIP-seq), targeted promoter reporter assays, or epigenetic compound screening are conducted here to distinguish direct HDAC-mediated PFN2 silencing from indirect transcriptional feedback. This unclarified upstream regulatory pathway for PFN2 constitutes a notable limitation of the current work. Follow-up epigenetic profiling and locus-specific functional assays are required to delineate whether HDAC inhibitory activity of VPA directly governs PFN2 transcription or mediates PFN2 suppression through secondary intermediate signaling events. Two distinct mechanistic models may account for PFN2 downregulation. On one hand, VPA-mediated Class I HDAC inhibition may directly remodel histone acetylation at the PFN2 promoter, reducing chromatin accessibility and repressing its transcription in a locus-specific manner. On the other hand, PFN2 suppression may represent an indirect secondary response triggered by widespread cellular stress, cell-cycle arrest or apoptosis induced by VPA treatment, rather than a targeted epigenetic effect on the PFN2 gene itself. Current data cannot discriminate between these two scenarios, which further highlights the necessity of promoter reporter assays and ChIP-seq analysis in future investigations.

However, multiple critical limitations restrict the translational and mechanistic interpretation of the present study. First, only three PDX models derived from EBV-positive pediatric BL patients are established for functional evaluation; this small cohort cannot capture the full molecular, pathological and clinical heterogeneity of BL, such as EBV-negative, adult and immunodeficiency-associated subtypes. Second, transcriptomic RNA-seq analysis is exclusively conducted on tumor tissue harvested from PDX No.1, which limits the generalizability of the identified PFN2-centered transcriptional signature across all patient-derived tumor lines. Third, formal pharmacokinetic profiling of VPA in mouse plasma, tumor interstitial fluid or human patient samples is absent, which prevents quantitative correlation between *in vivo* drug exposure and observed anti-tumor efficacy. Fourth, effective anti-BL millimolar VPA concentrations identified *in vitro* and *ex vivo* HDST assays cannot be readily reproduced via conventional clinical oral dosing due to interspecies differences in drug clearance, plasma protein binding and narrow human therapeutic windows, raising uncertainty regarding whether sufficient intratumoral drug exposure can be safely attained in BL patients. Fifth, all *in vivo* efficacy assessments rely on subcutaneous tumor xenografts grown in immunodeficient NOD-SCID mice; these models fail to recapitulate intact human anti-tumor immune circuits and native lymphoid tissue microenvironment where clinical BL lesions develop, inevitably altering tumor-drug interactions and tumor progression patterns. Sixth, only one PFN2-targeted shRNA construct is primarily utilized for gene knockdown assays, which carries inherent risks of off-target transcriptional interference that may confound observed cellular phenotypes. Collectively, these constraints highlight that the current preclinical findings only provide preliminary correlative evidence for VPA repurposing in BL, and larger multi-subtype PDX cohorts, comprehensive pharmacokinetic testing, immunocompetent humanized tumor models and standard chemotherapy comparator arms are required in follow-up research to resolve the above deficiencies.

## 5 Conclusion

This study confirmed that VPA could inhibit cell proliferation and promote cell apoptosis in EBV-positive BL cell lines. And VPA also inhibited tumor growth in PDX and CDX models. The phenotypic changes observed upon VPA treatment were associated with altered PFN2 expression and PI3K/AKT signalling activity. Additional temporal tracking, full rescue and pathway-dependence studies are required to determine whether PFN2 mediates the antitumor effects of VPA.

## Data Availability

RNA-seq data have been deposited at GEO: https://www.ncbi.nlm.nih.gov/geo/query/acc.cgi?acc=GSE298448. Any additional information required to reanalyze the data reported in this paper is available from Aili He (heaili@xjtu.edu.cn).
