# Supplementary material for: Anti-tumor activity of valproic acid in EBV-positive Burkitt lymphoma via PFN2-associated PI3K/AKT pathway repression
Source: Front Pharmacol. 2026 Sep 14;17:1925552. doi: 10.3389/fphar.2026.1925552 (PMC13617333; doi:10.3389/fphar.2026.1925552)
Supplement: Supplementary file 1 [file DataSheet1.pdf]

VPA treated PDX tissue WB image

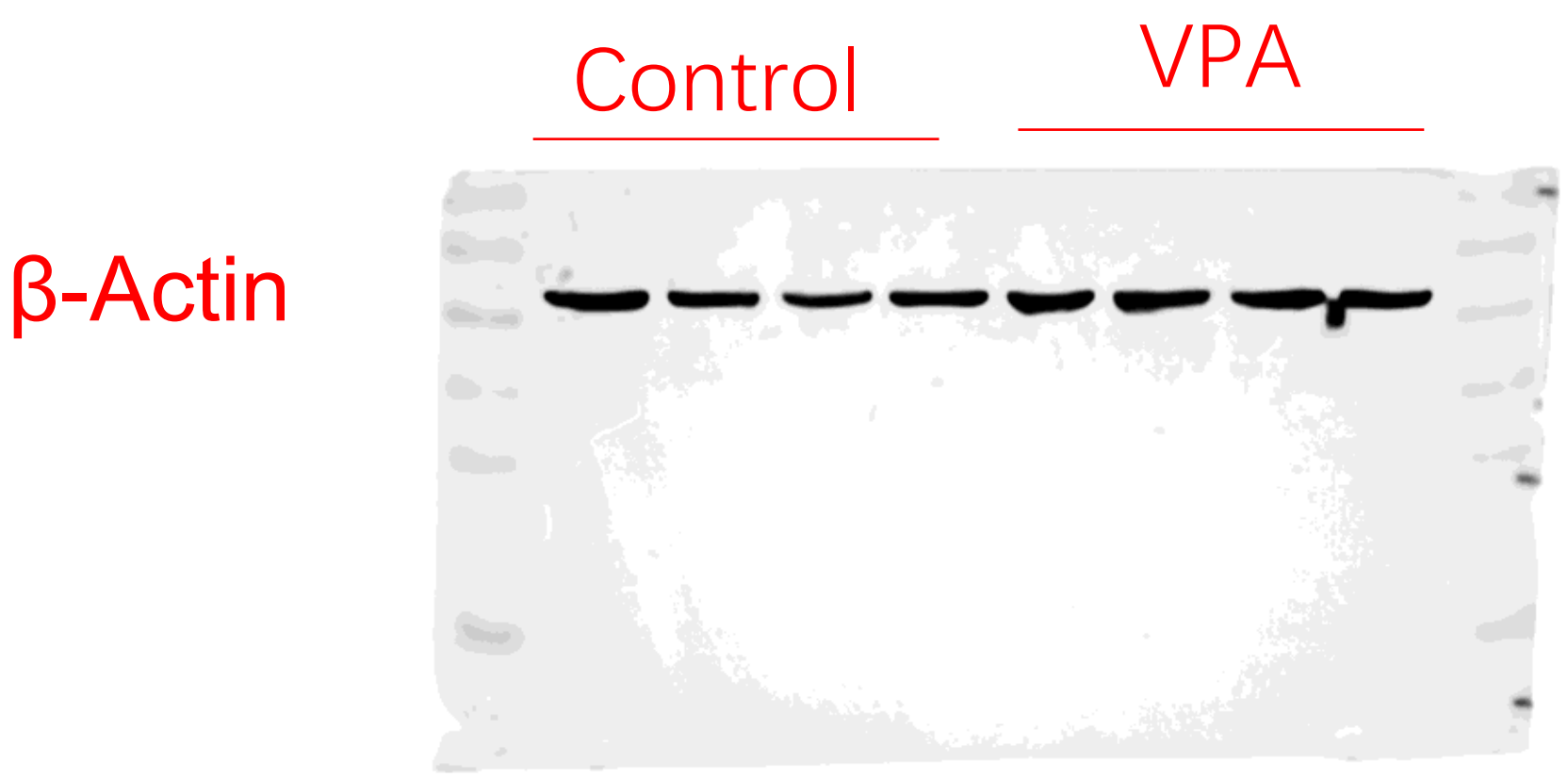

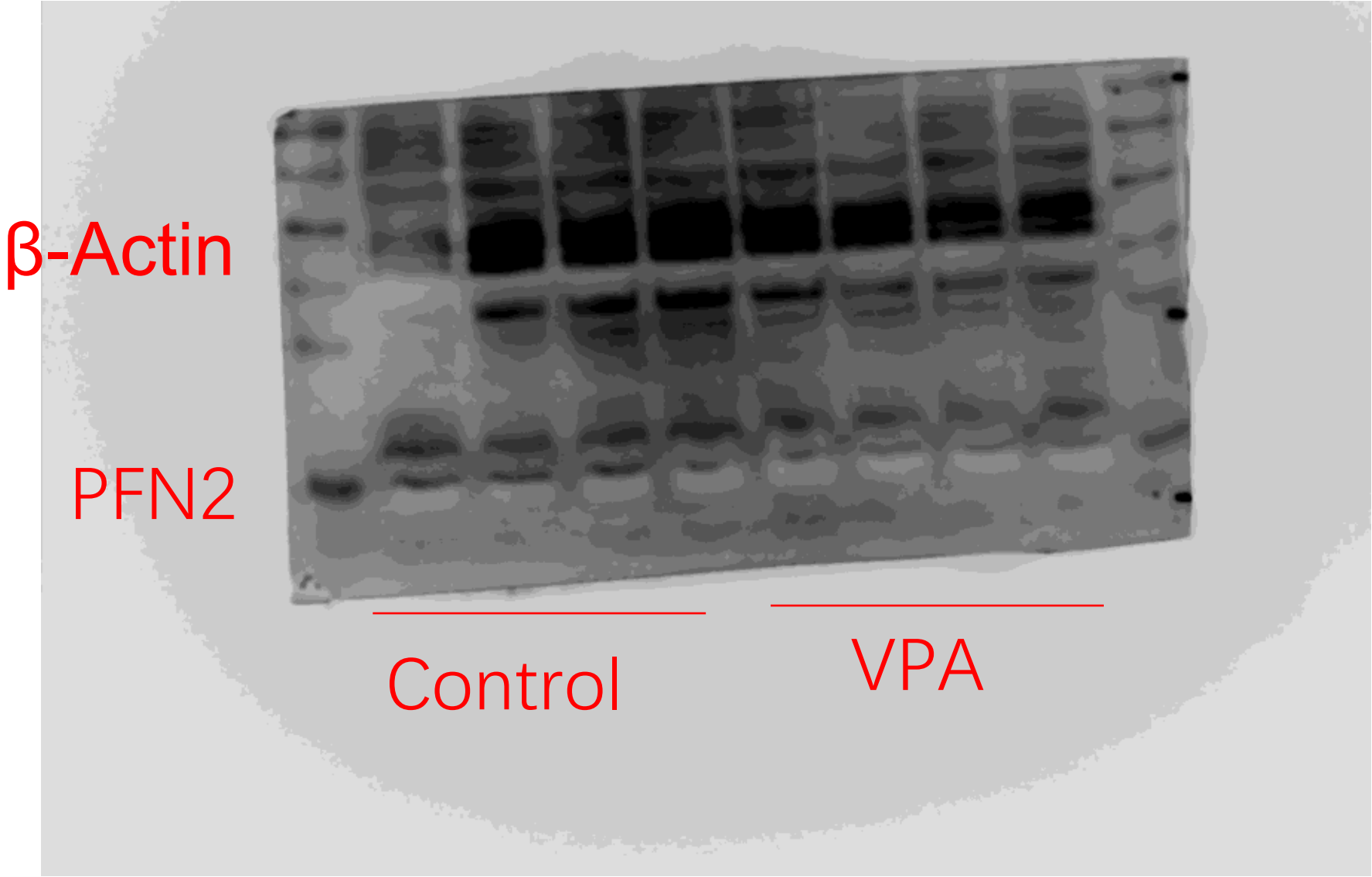

VPA treated cell line WB image

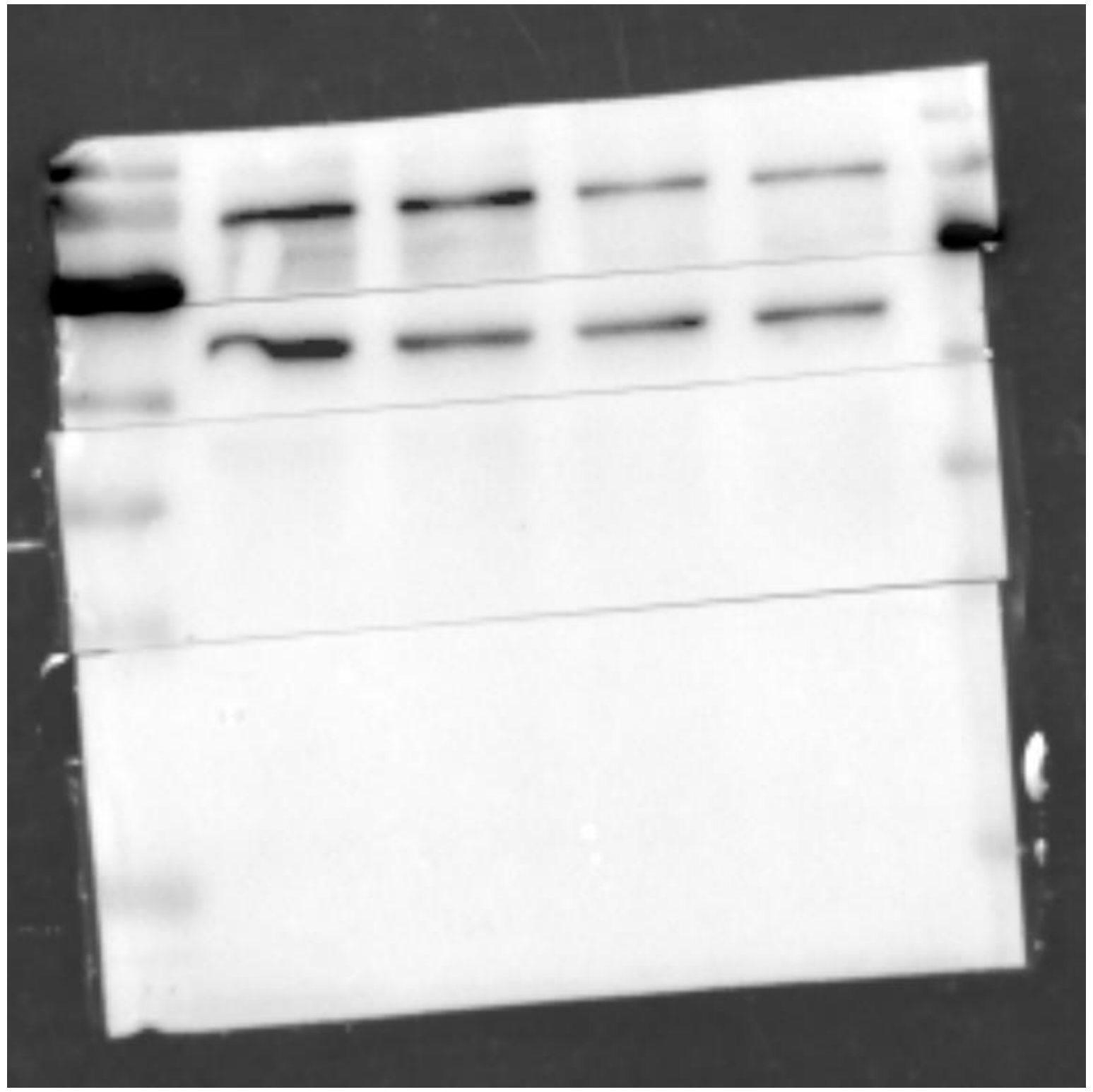

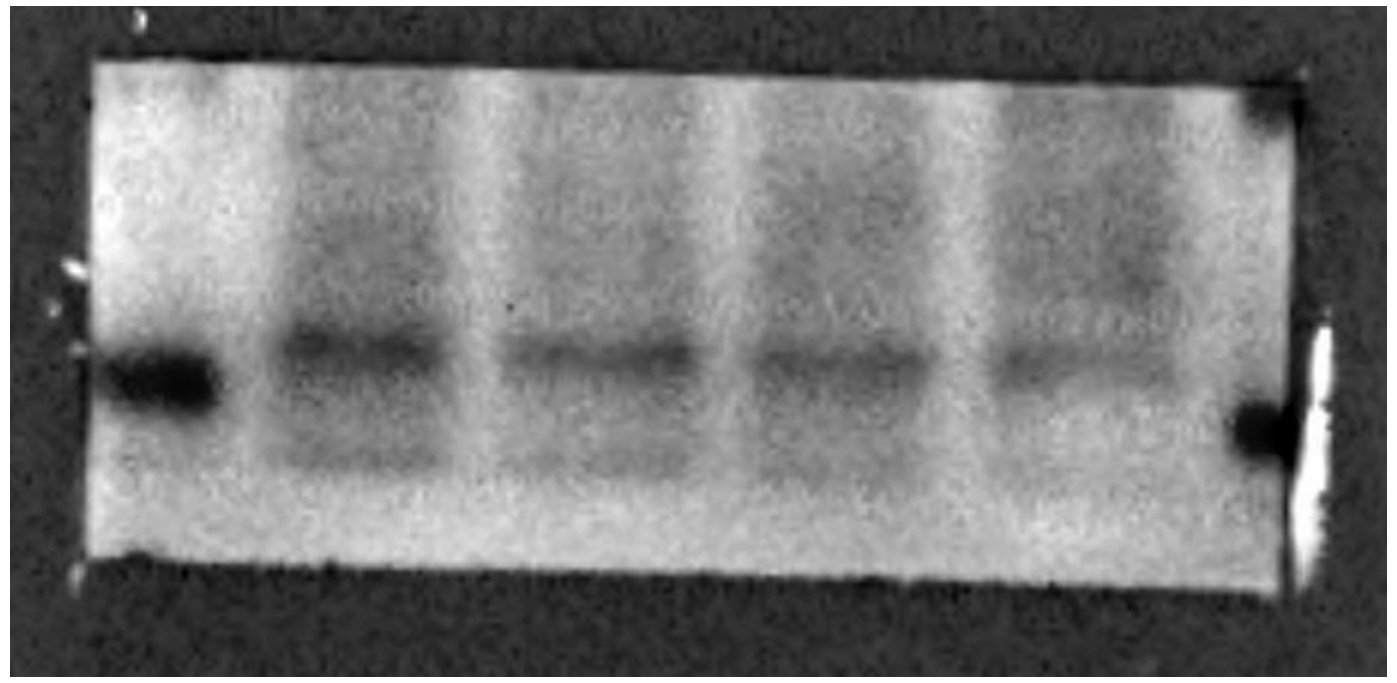

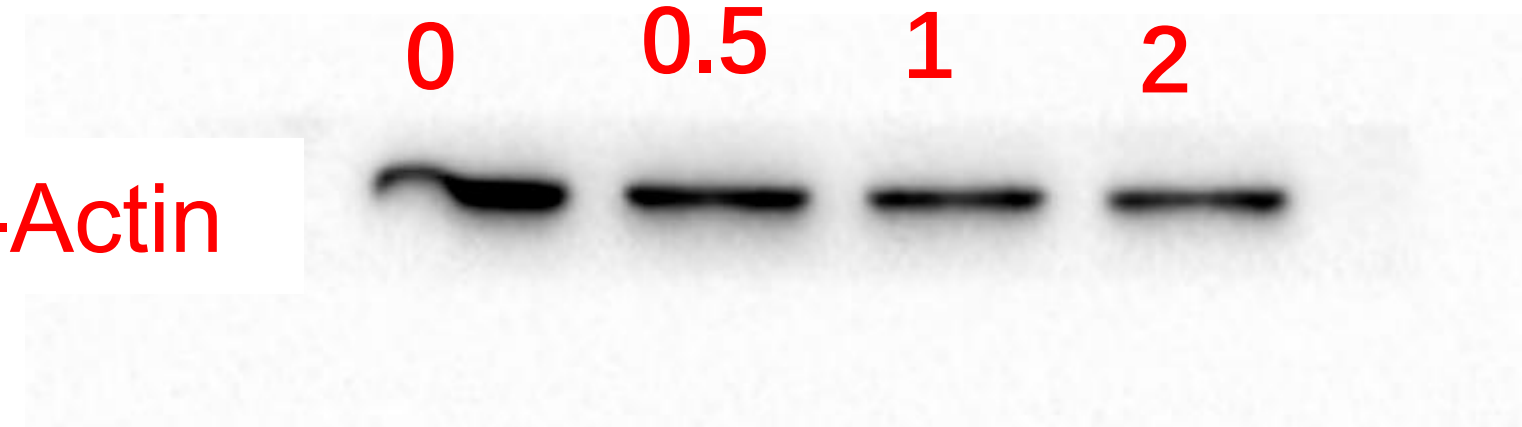

VPA treated cell line WB image

0 0.5 1 2

PFN2

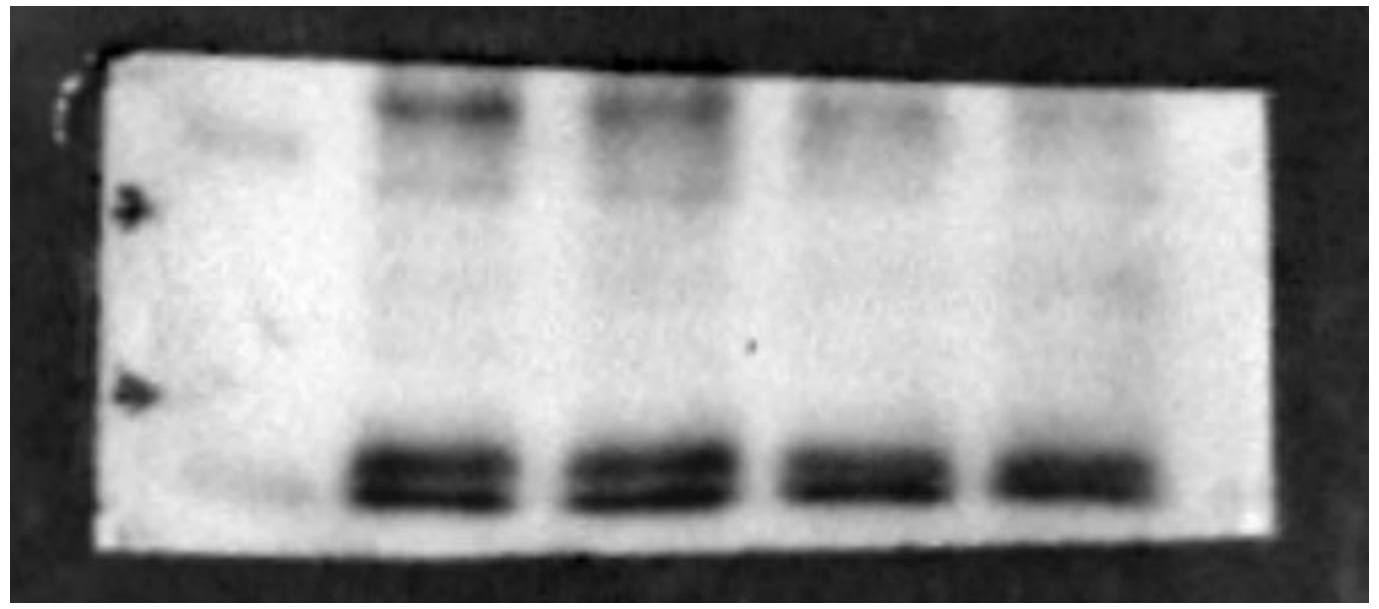

VPA

0 0.5 1 2

p-AKT

β-Actin

PFN2

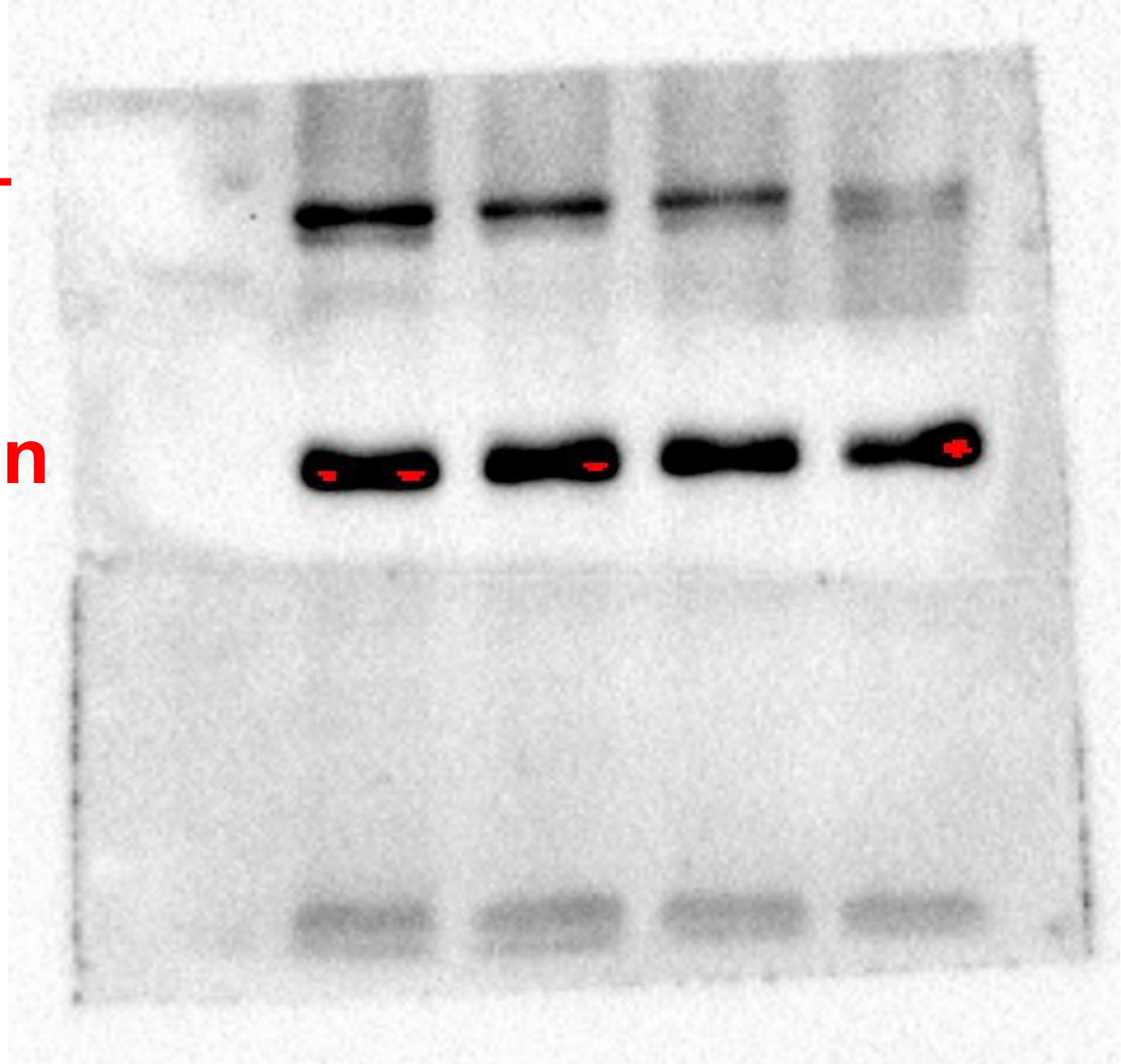

VPA treated cell line WB image

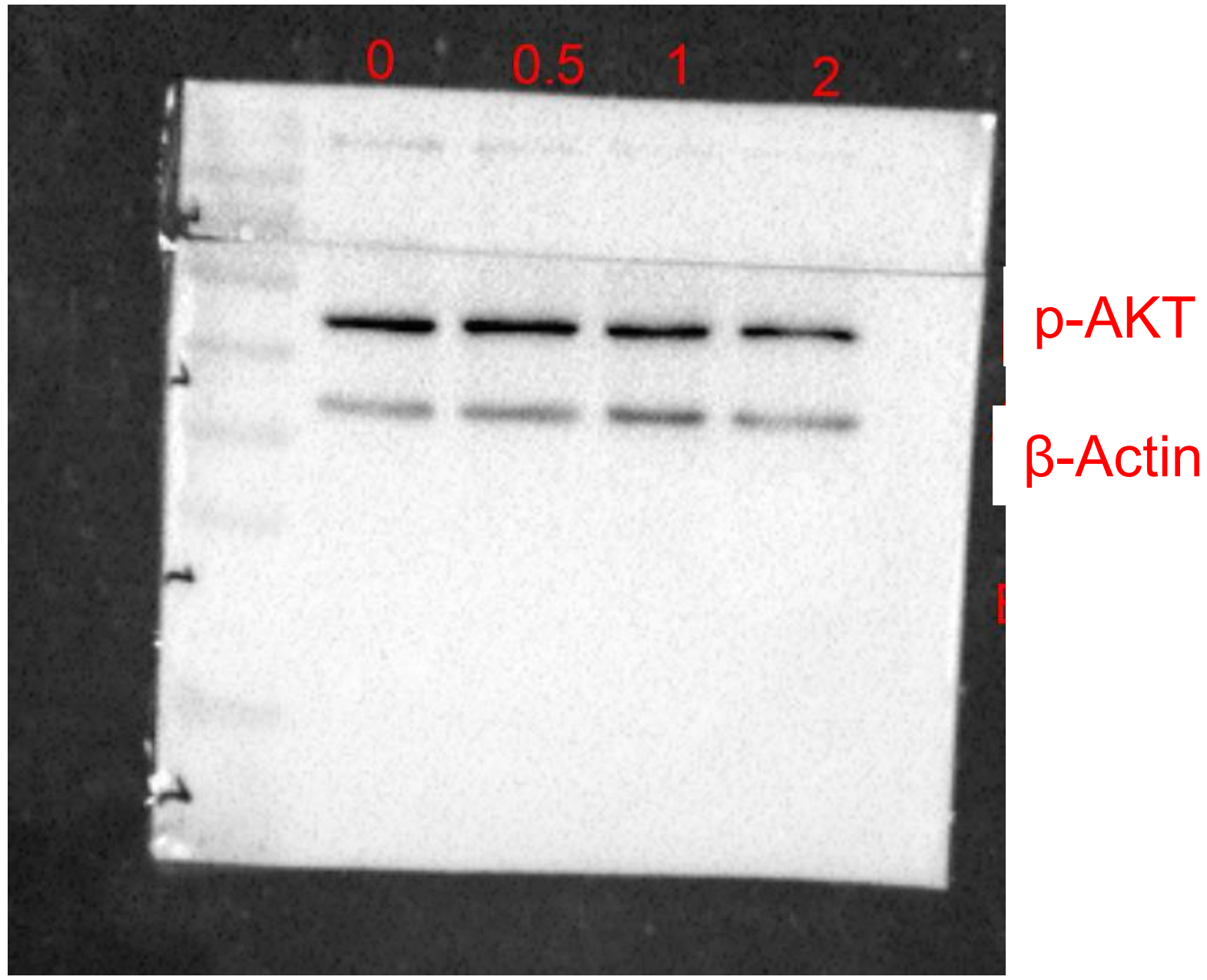

VPA treated cell line WB image

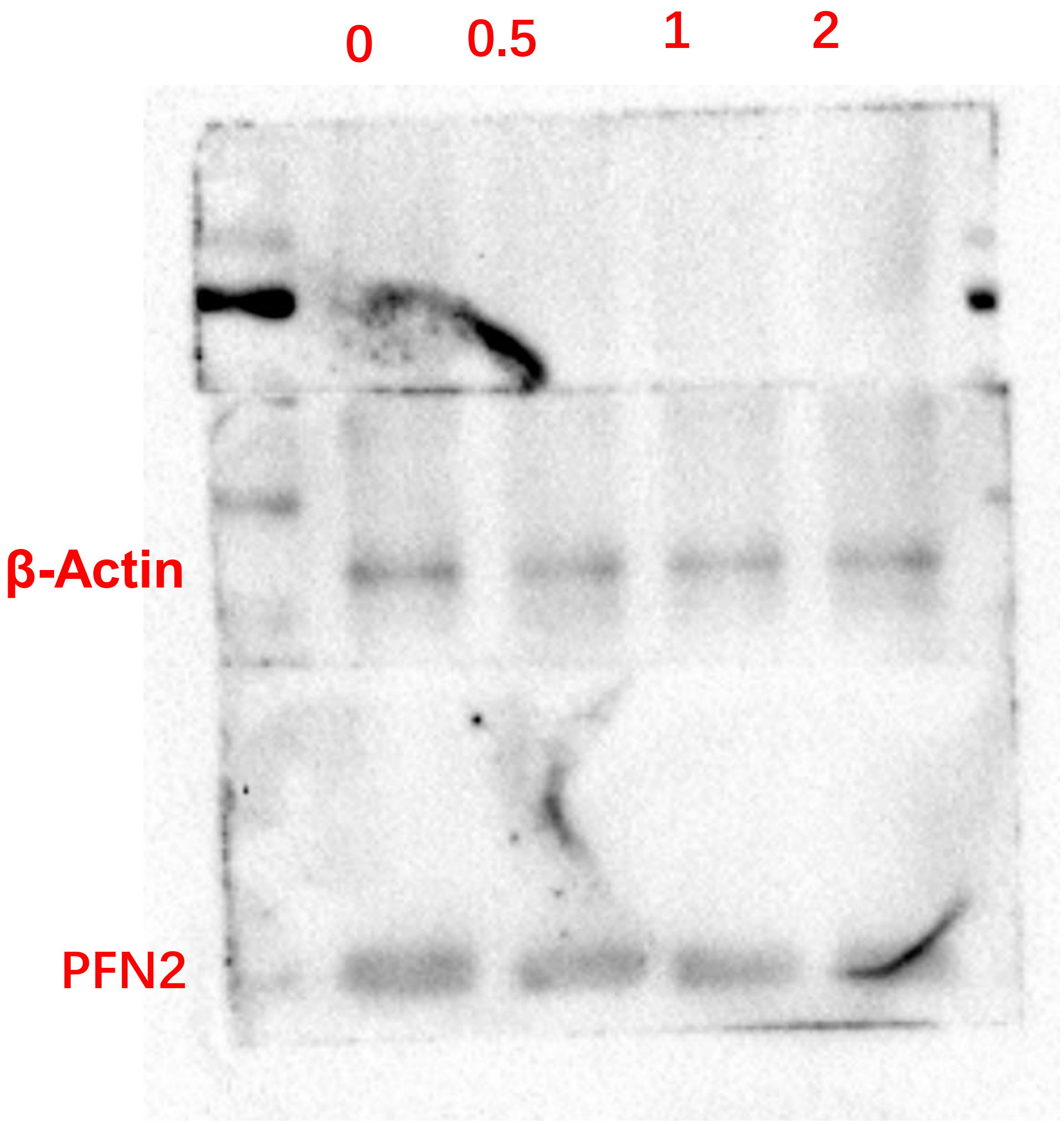

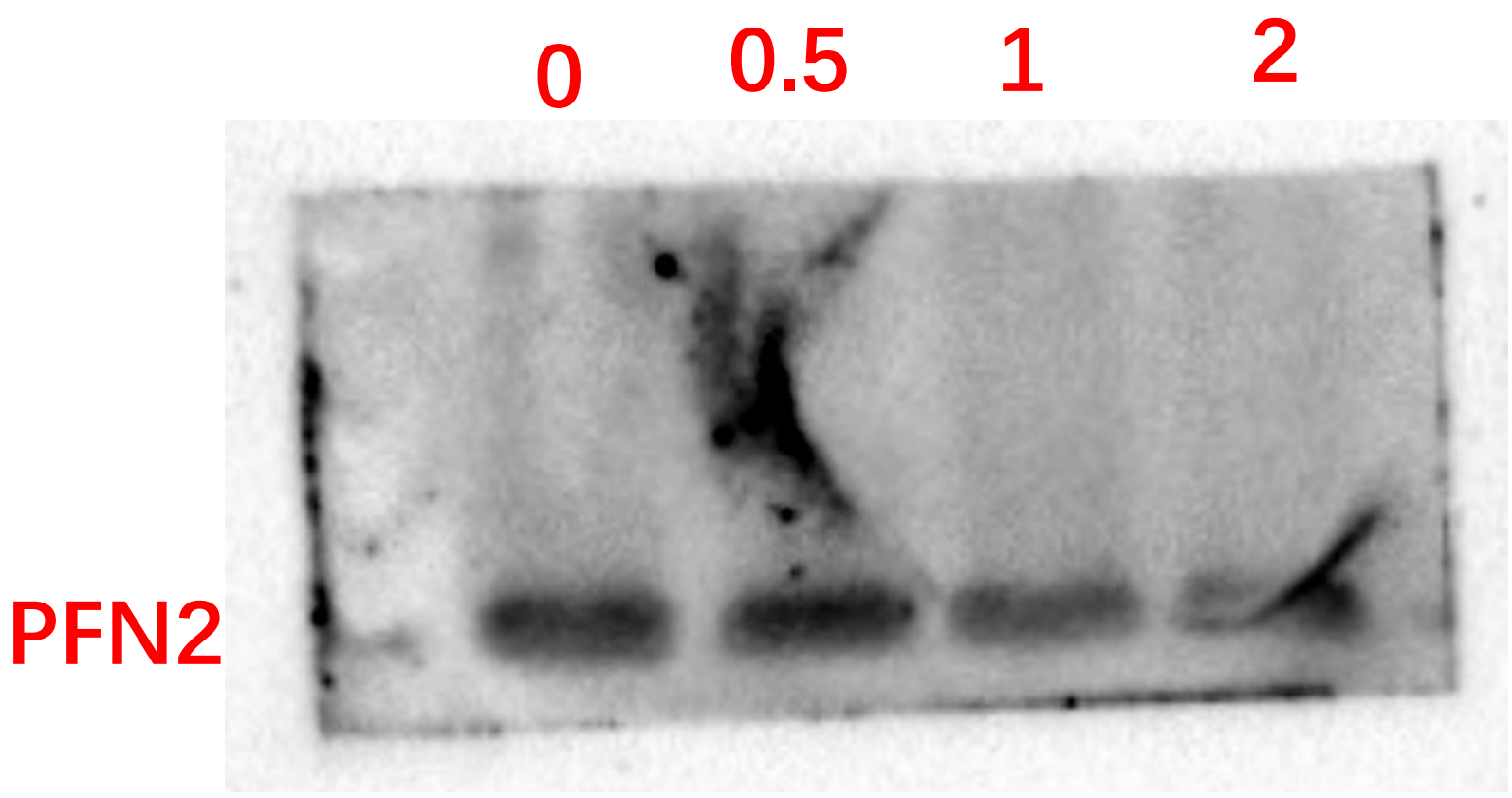

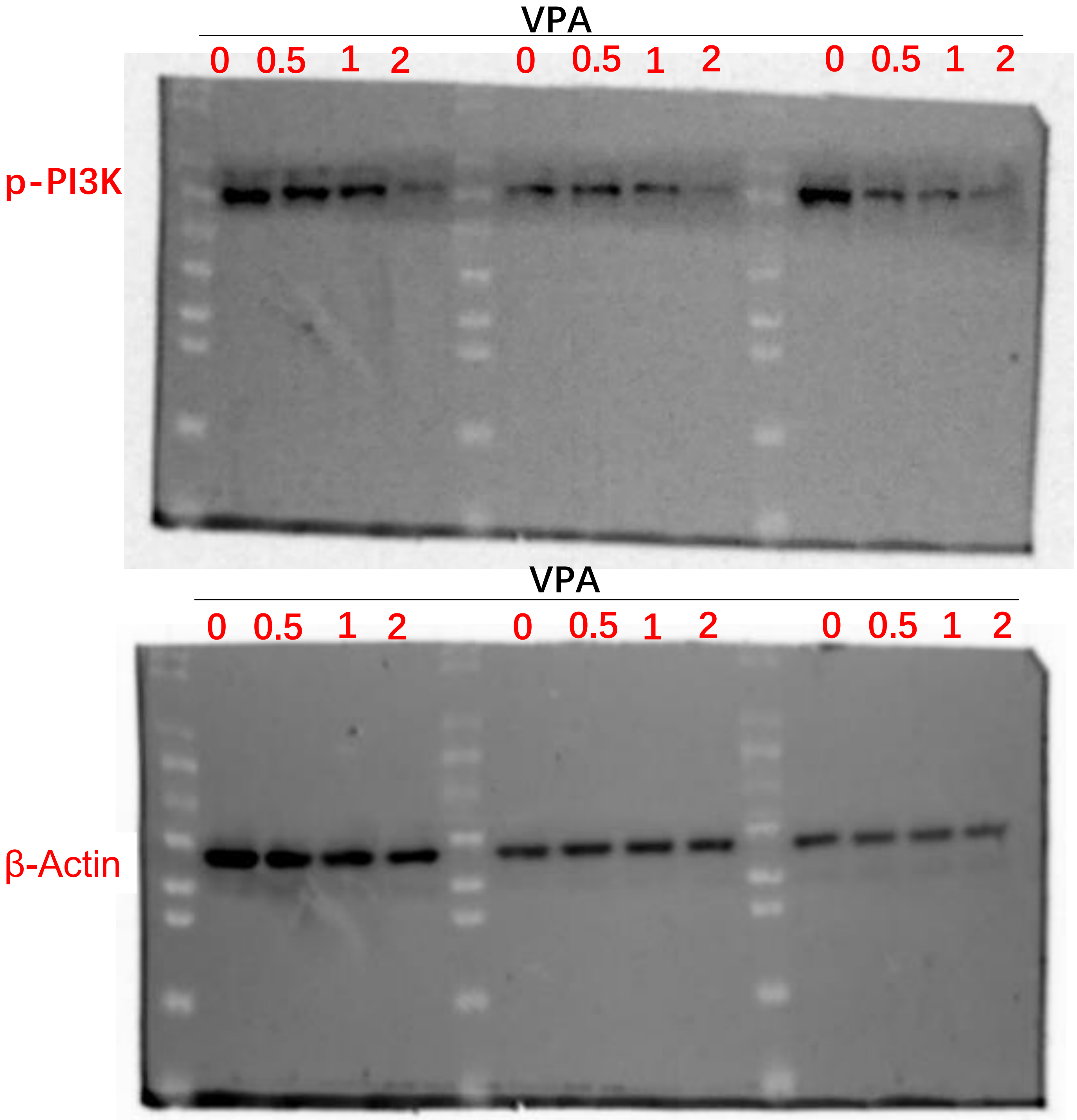

VPA treated cell line WB image

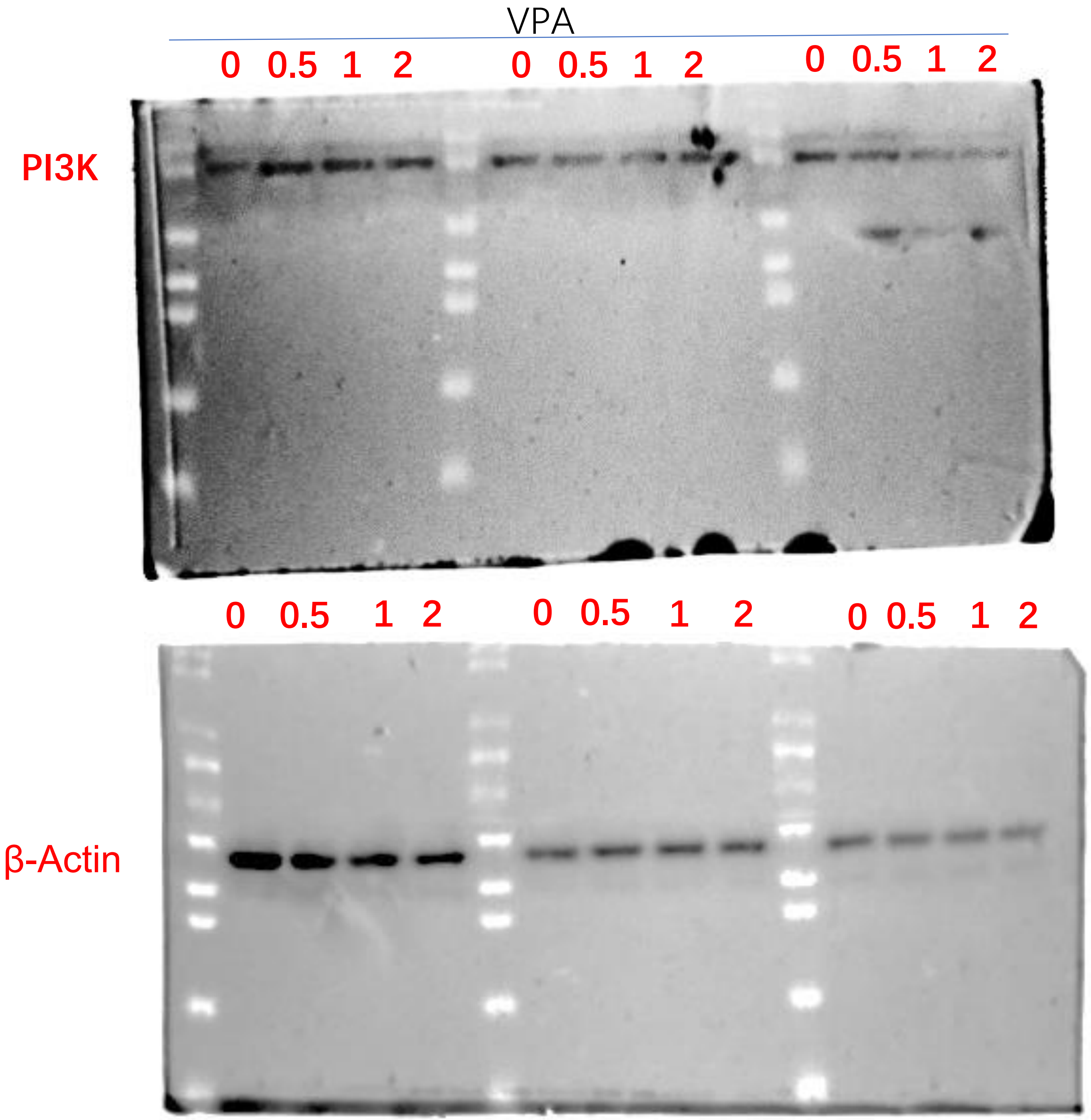

VPA treated cell line WB image

VPA treated cell line WB image

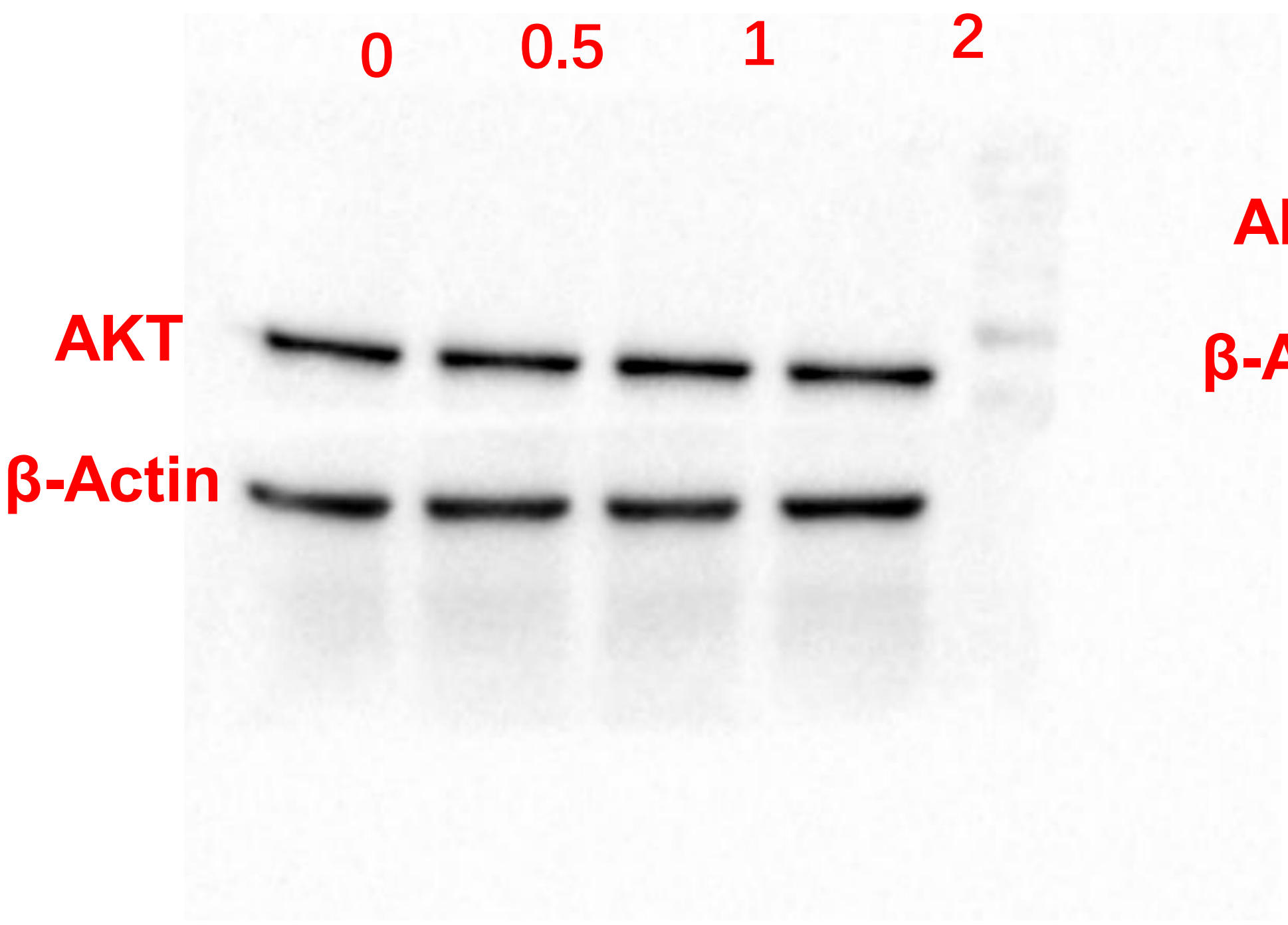

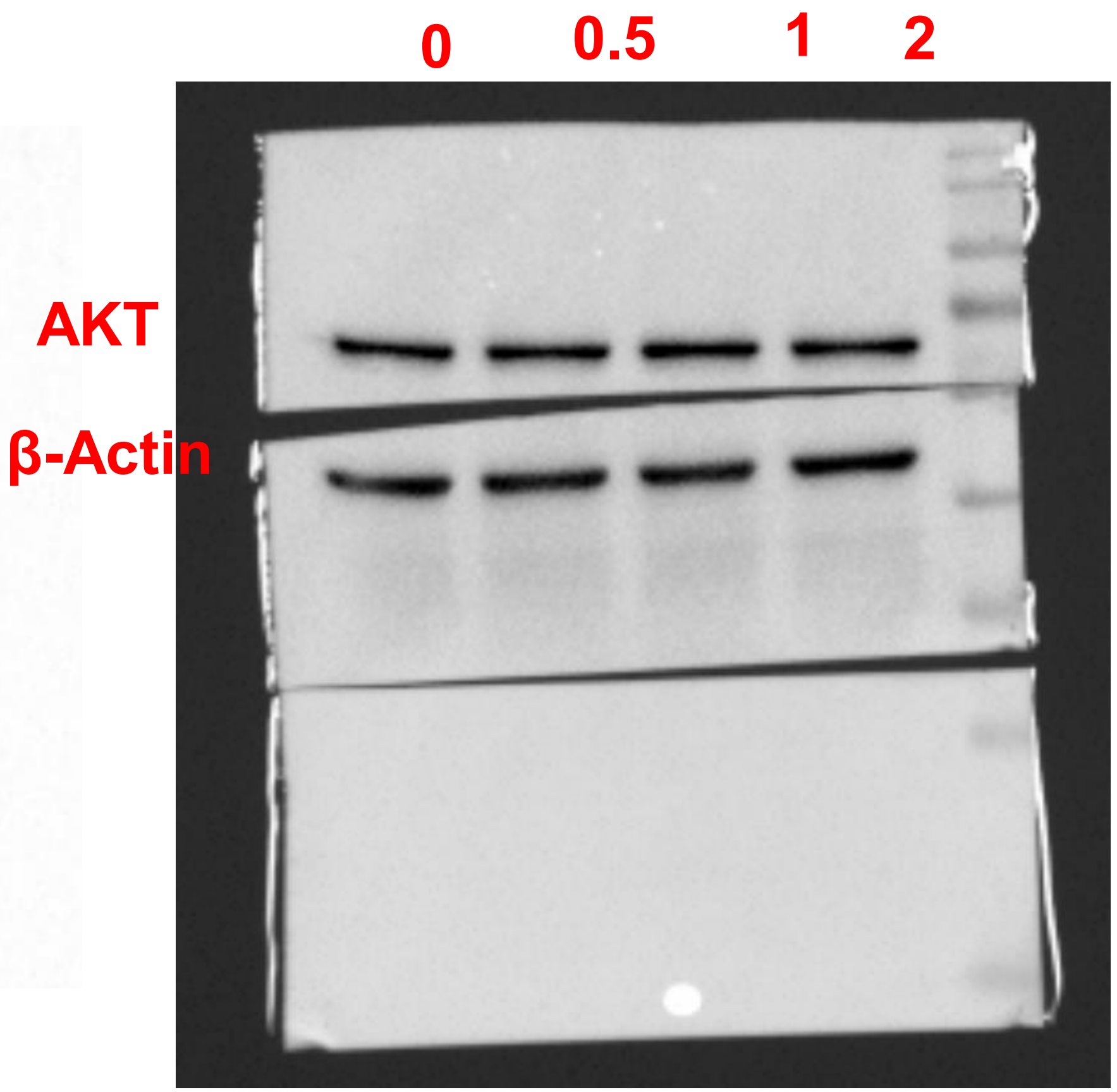

VPA treated cell line WB image

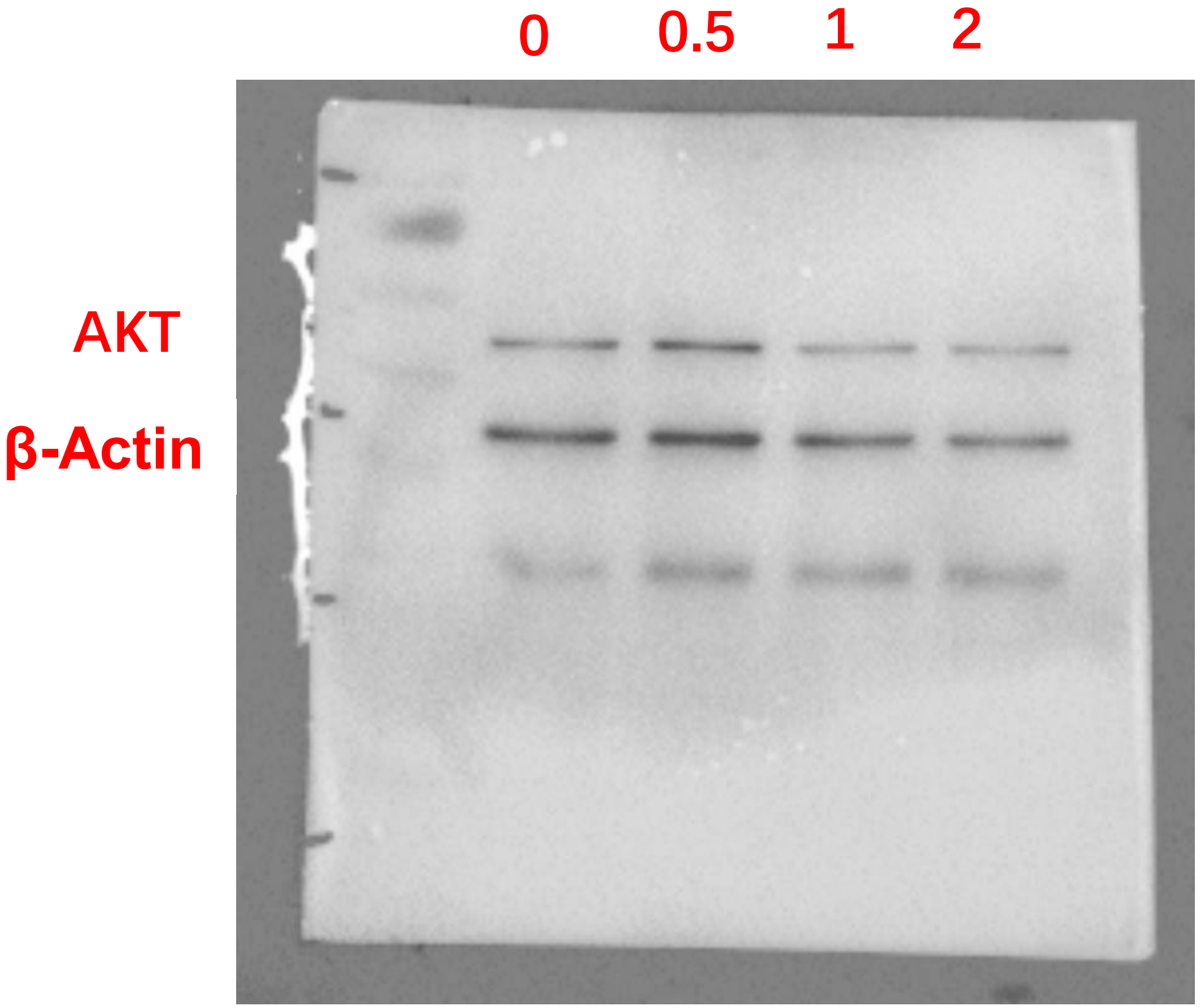

VPA treated cell line WB image

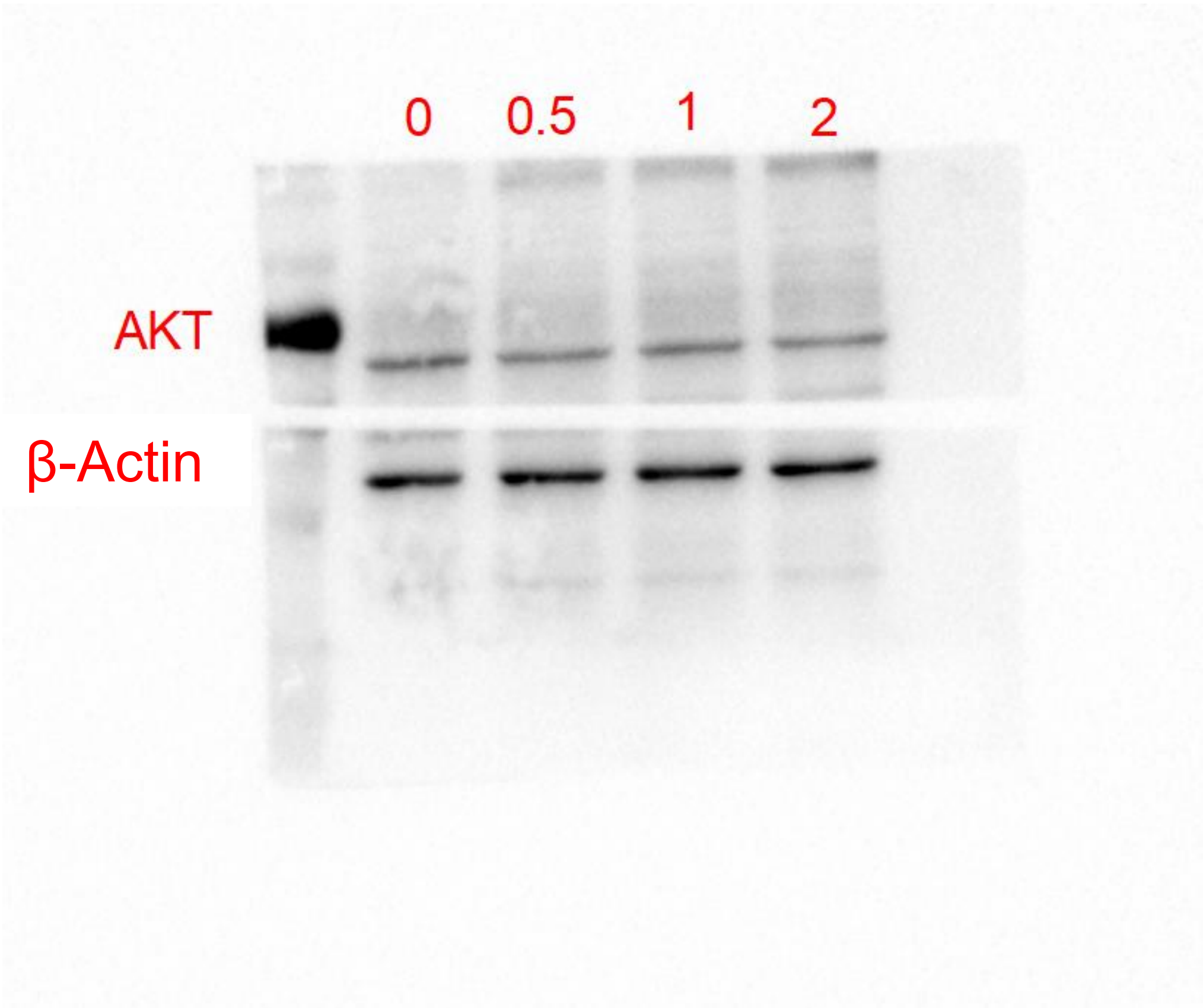

PFN2-KD cell line WB image

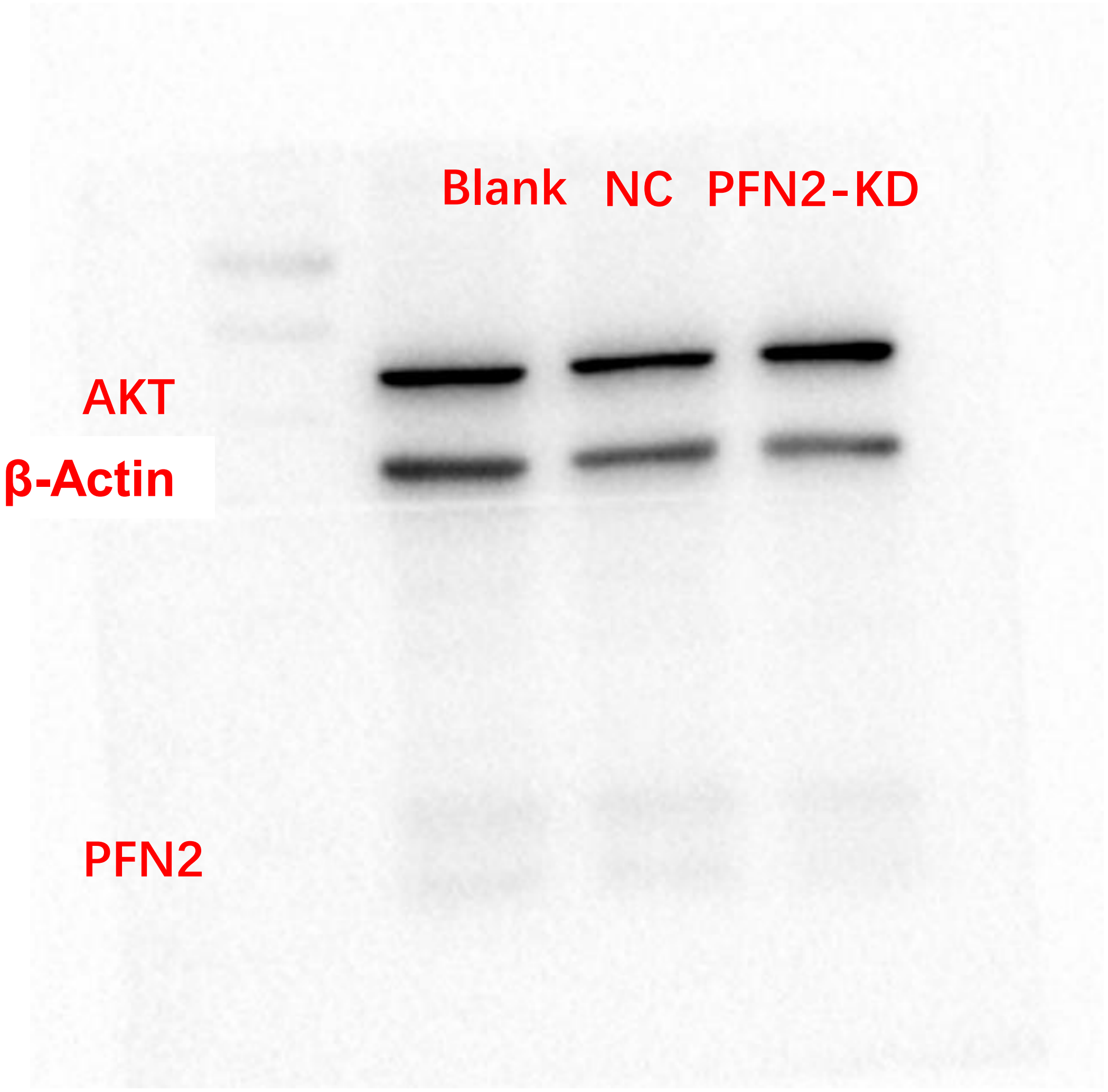

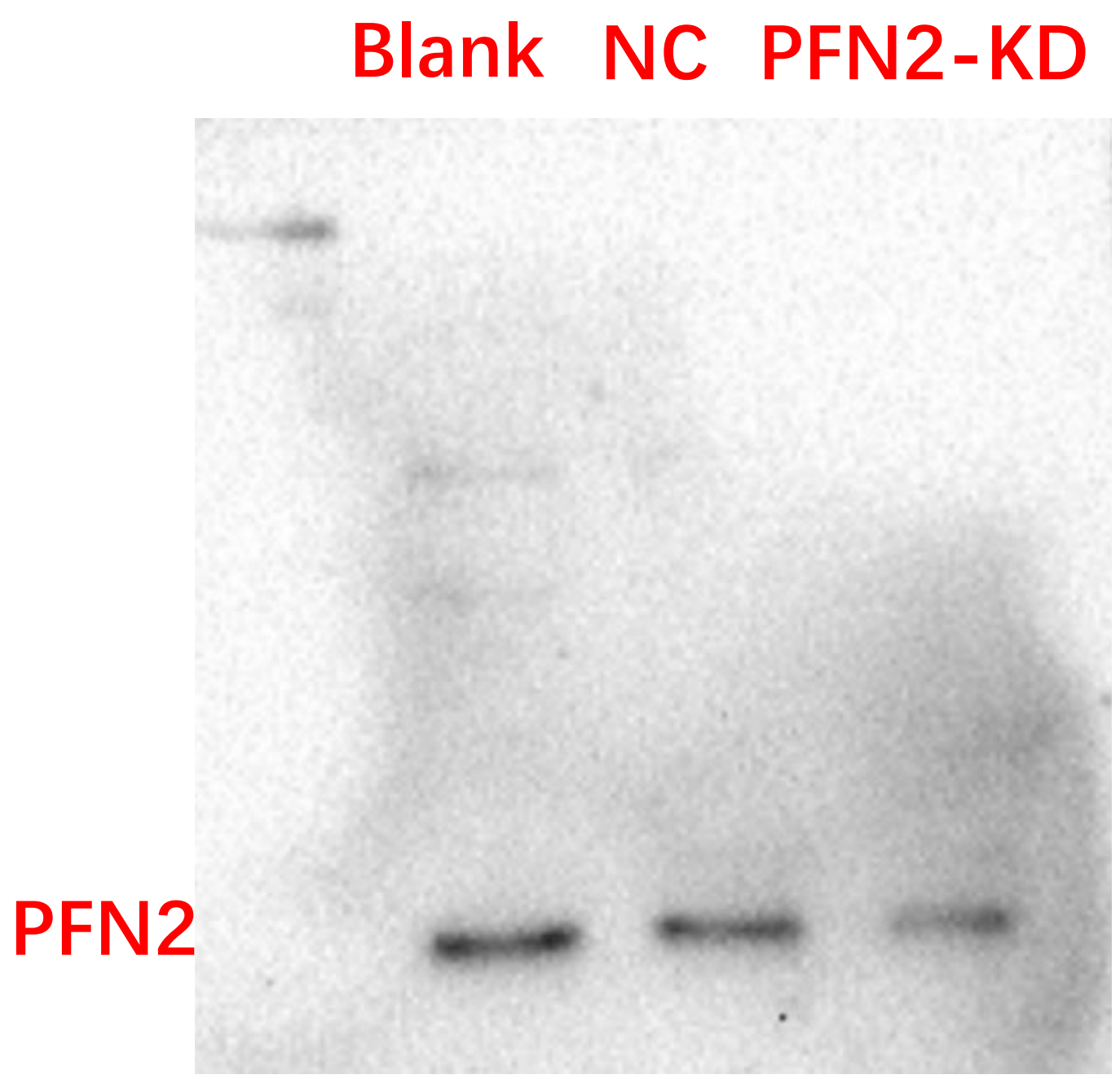

PFN2-KD cell line WB image

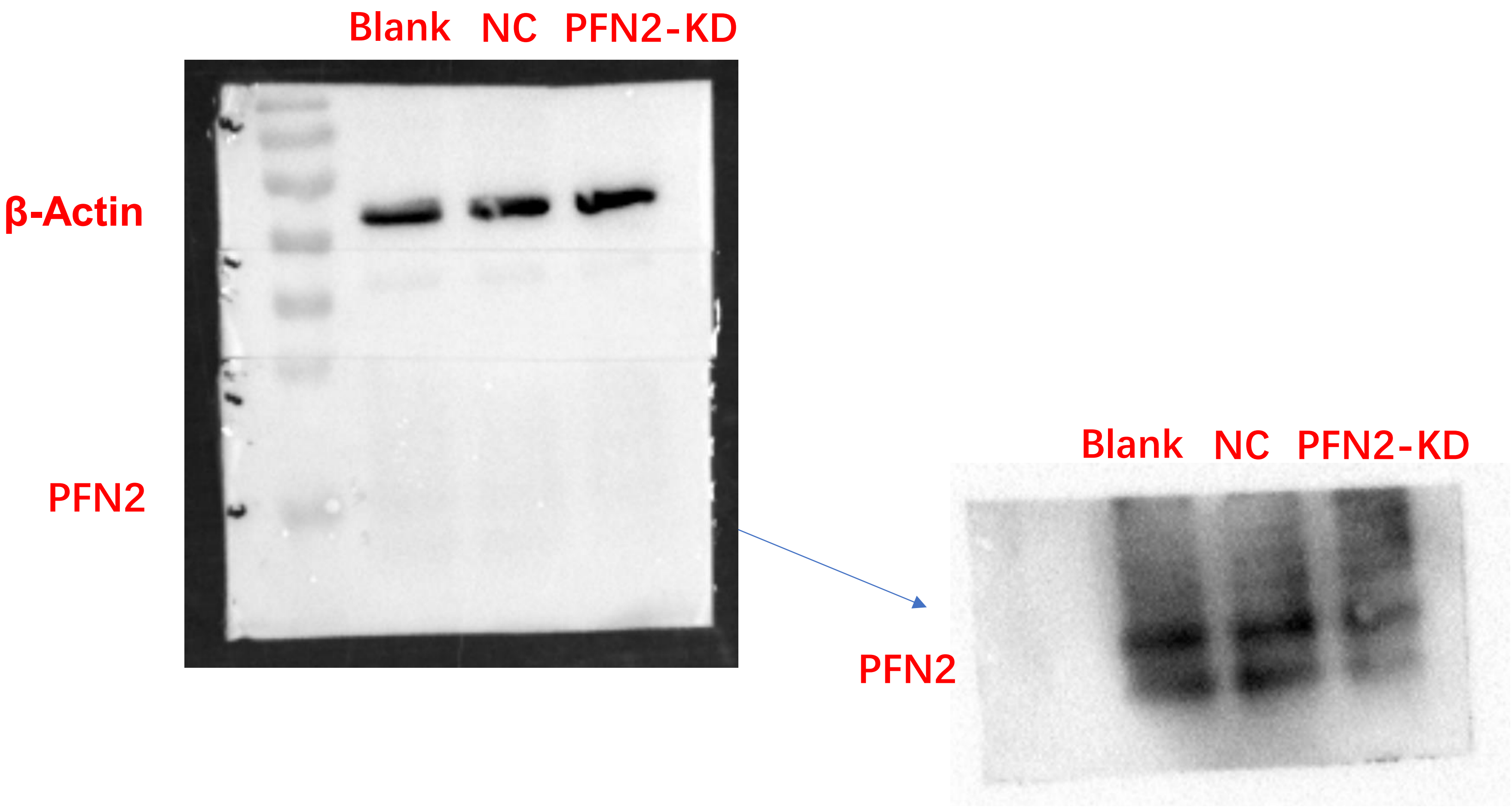

PFN2-KD cell line WB image

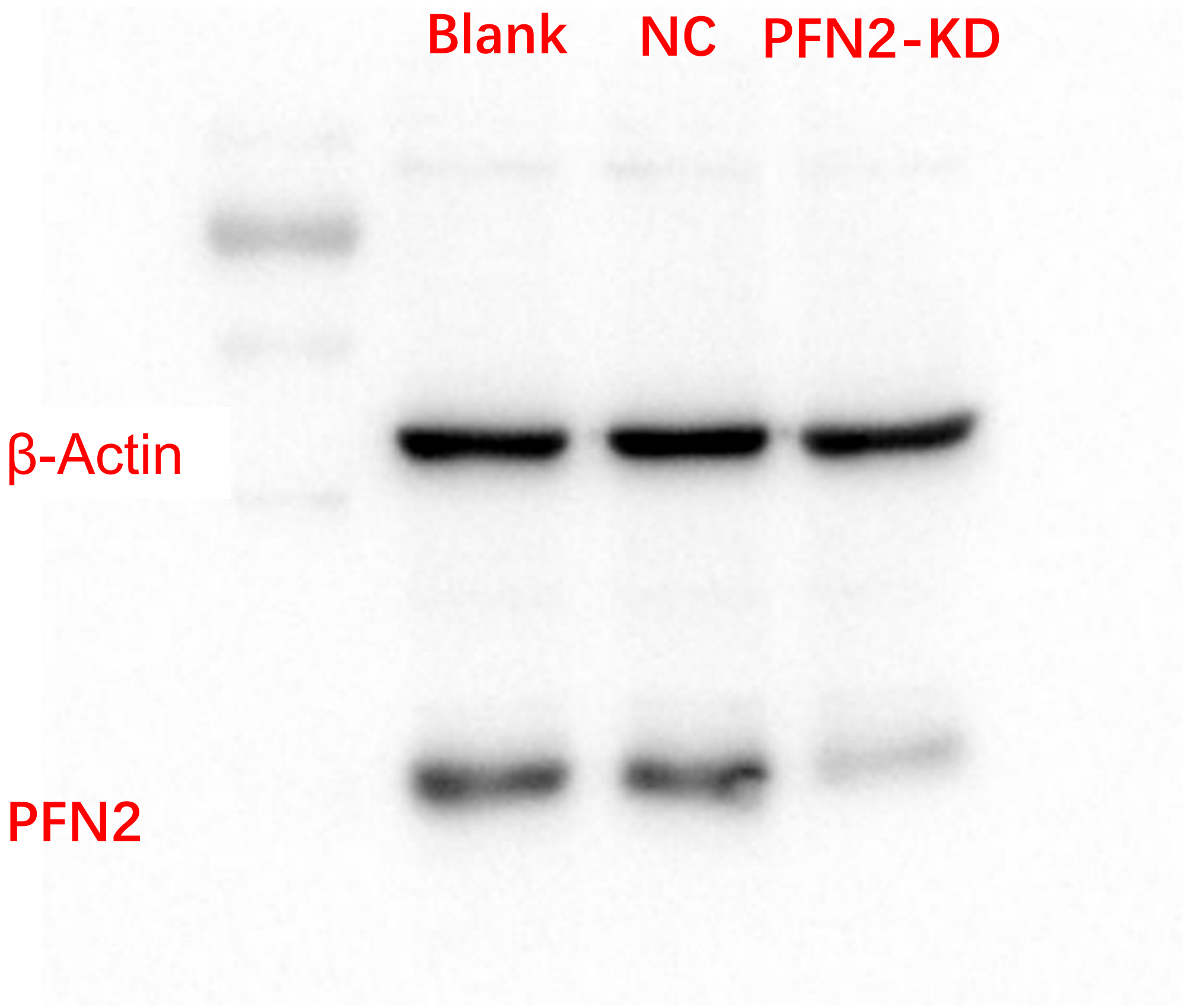

PFN2-KD cell line WB image

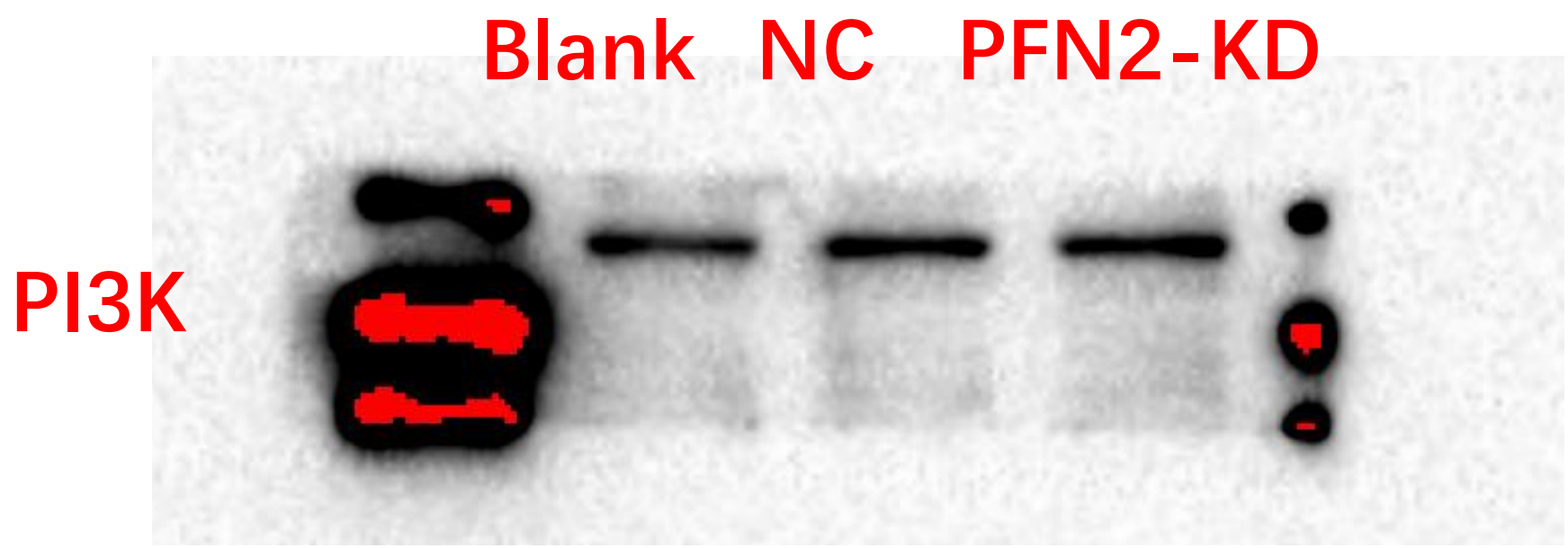

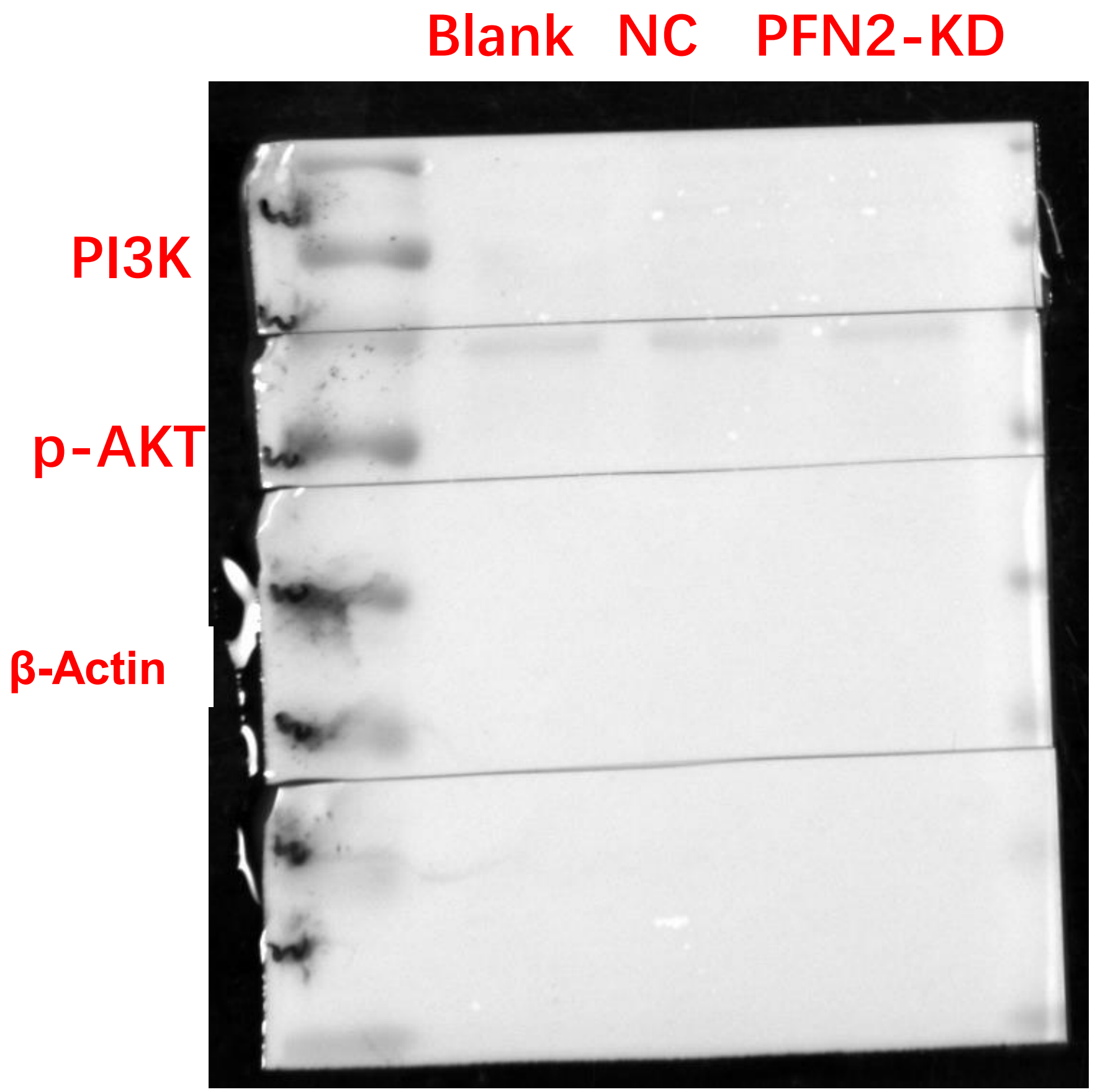

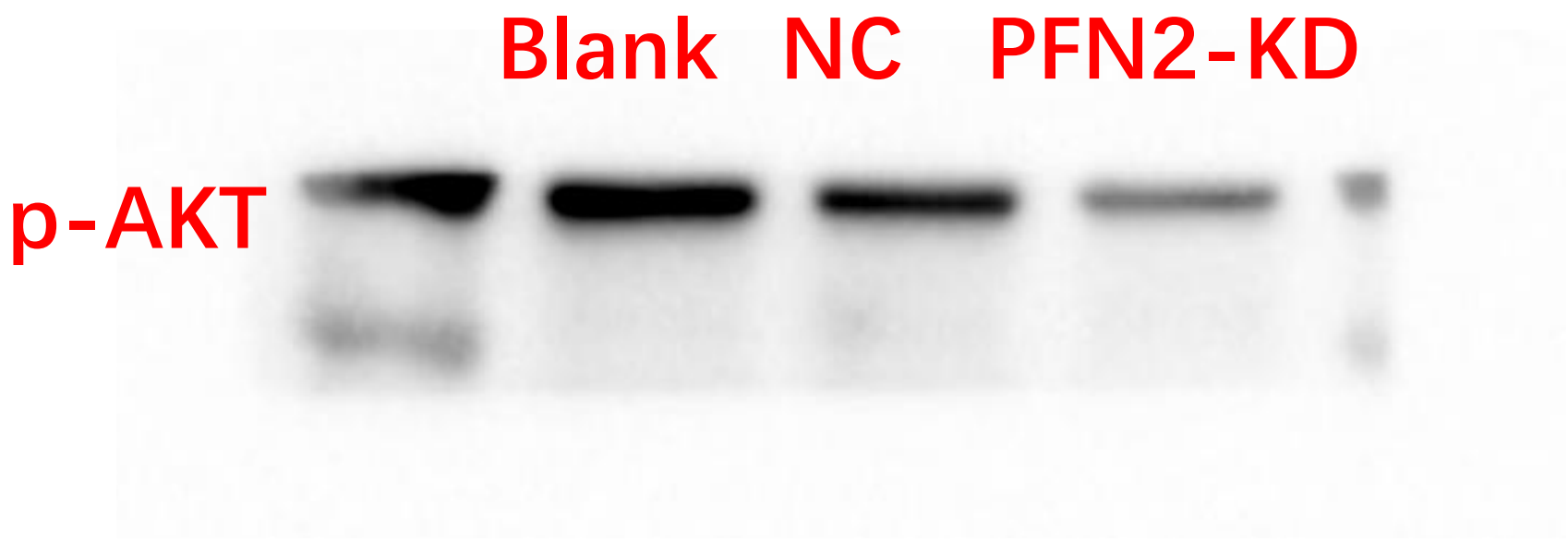

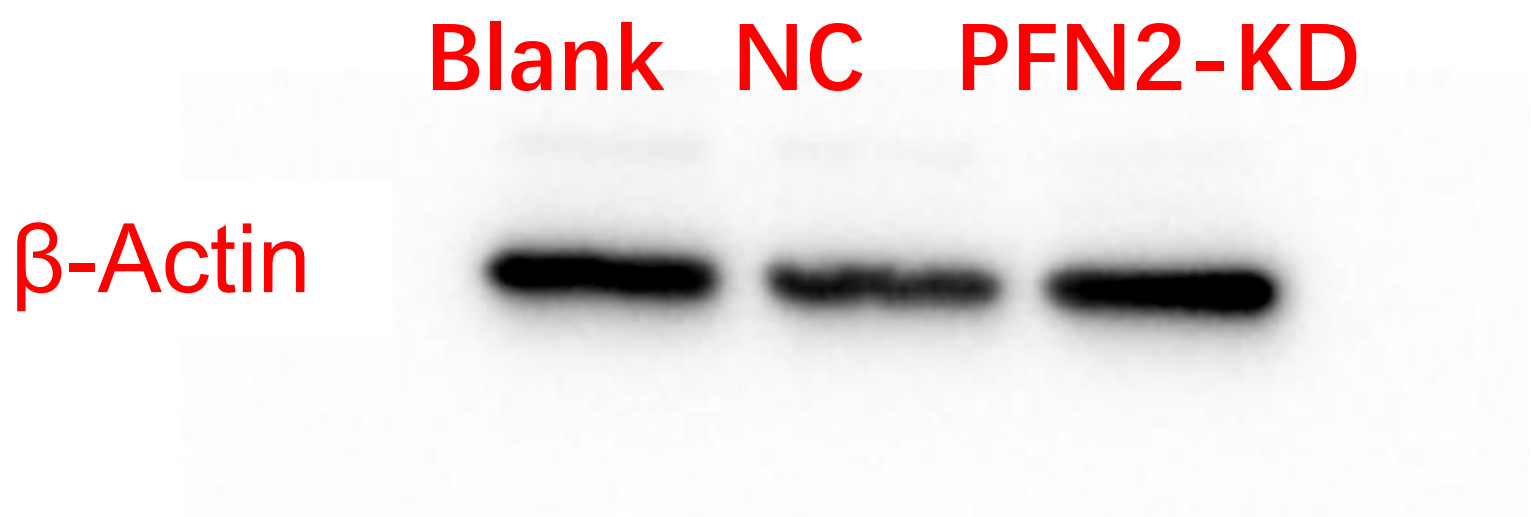

PFN2-KD cell line WB image

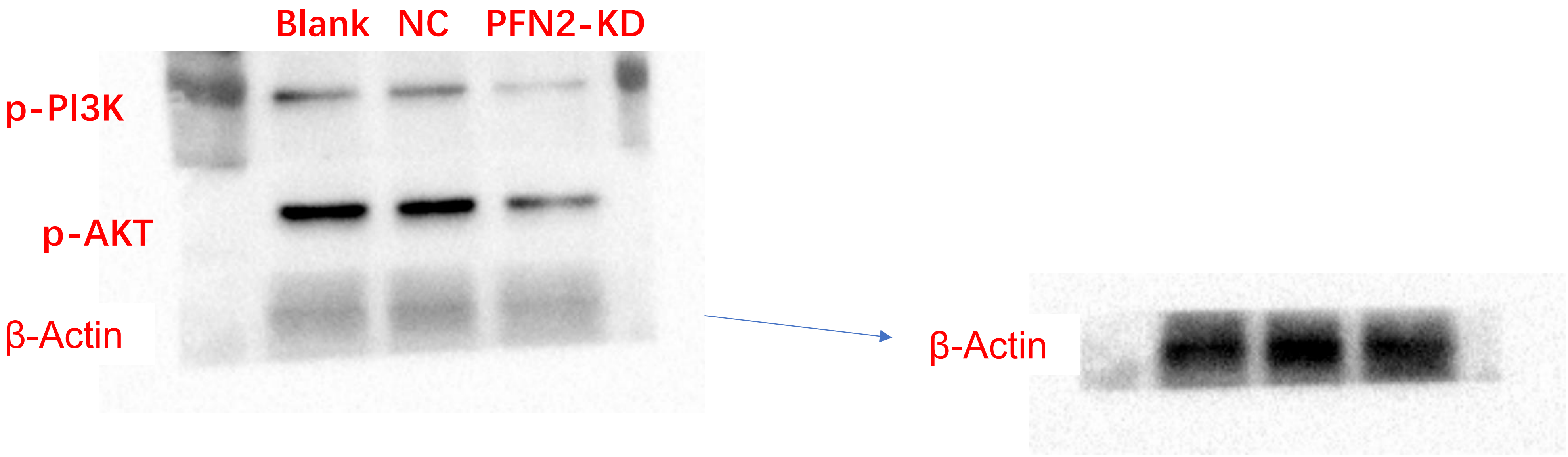

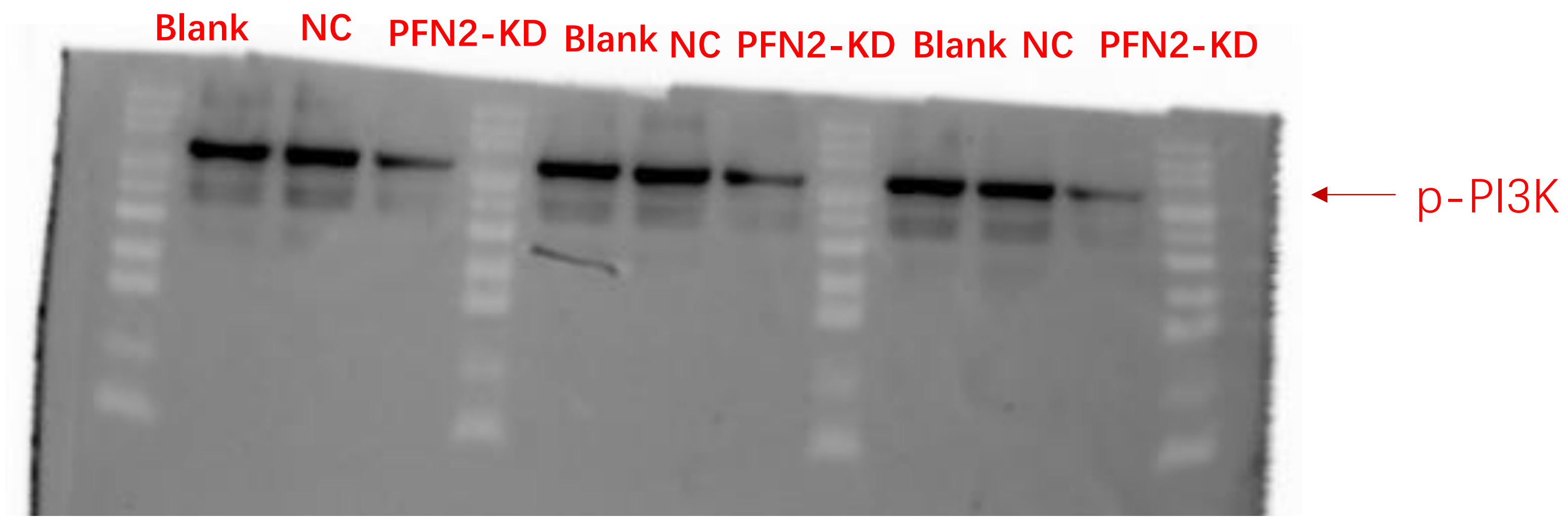
Blank
NC
PFN2-KD
Blank
NC
PFN2-KD
Blank
NC
PFN2-KD
p-PI3K

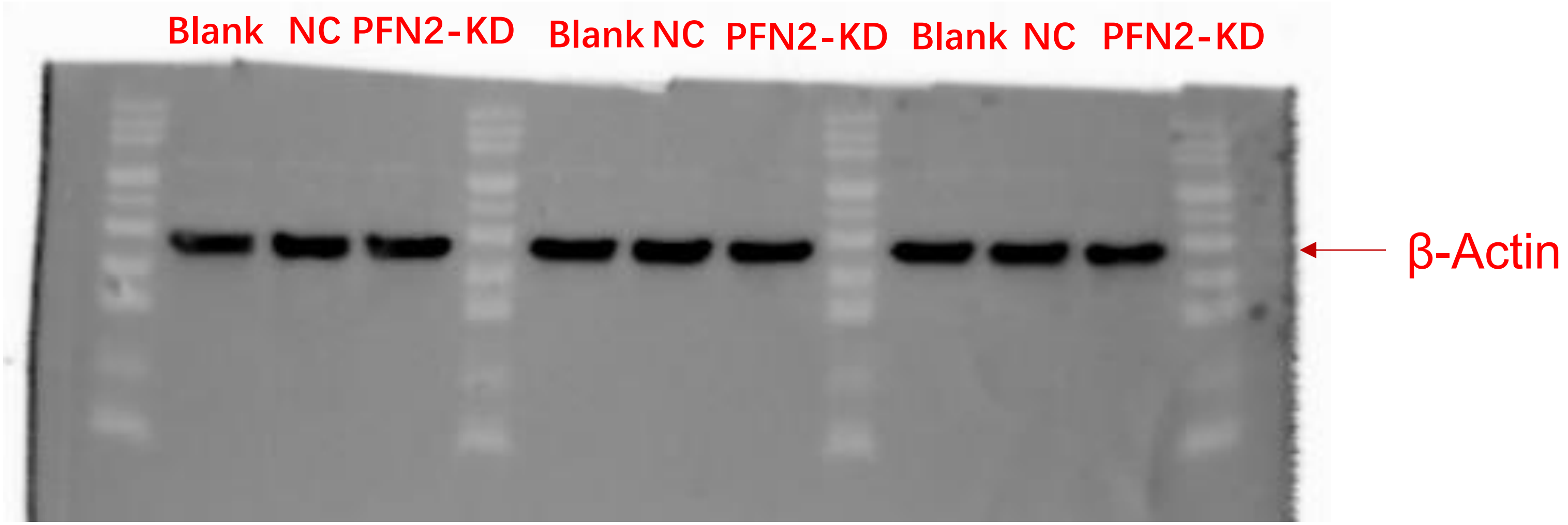
Blank
NC
PFN2-KD
Blank
NC
PFN2-KD
Blank
NC
PFN2-KD
β-Actin

PFN2-KD cell line WB image

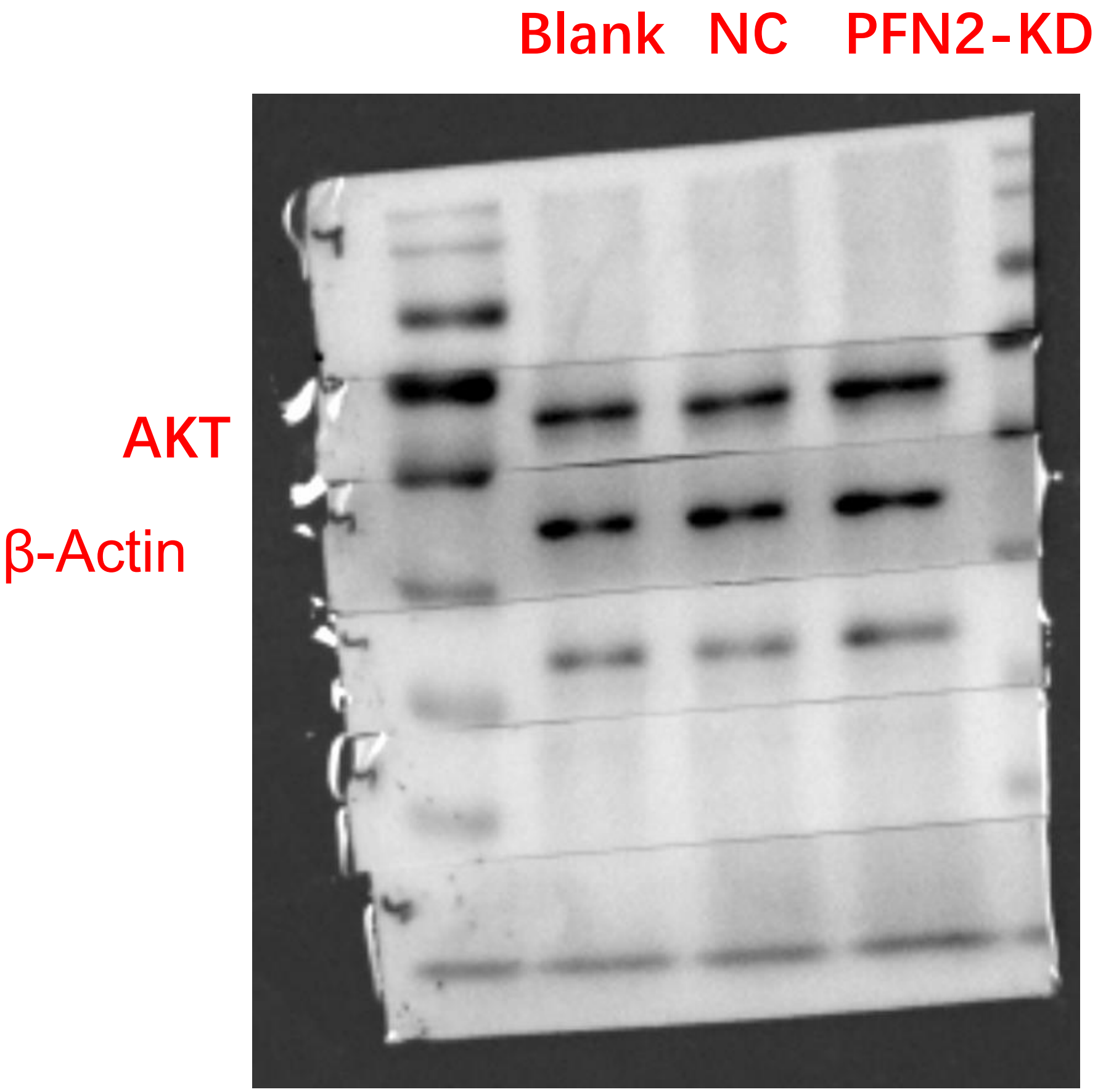

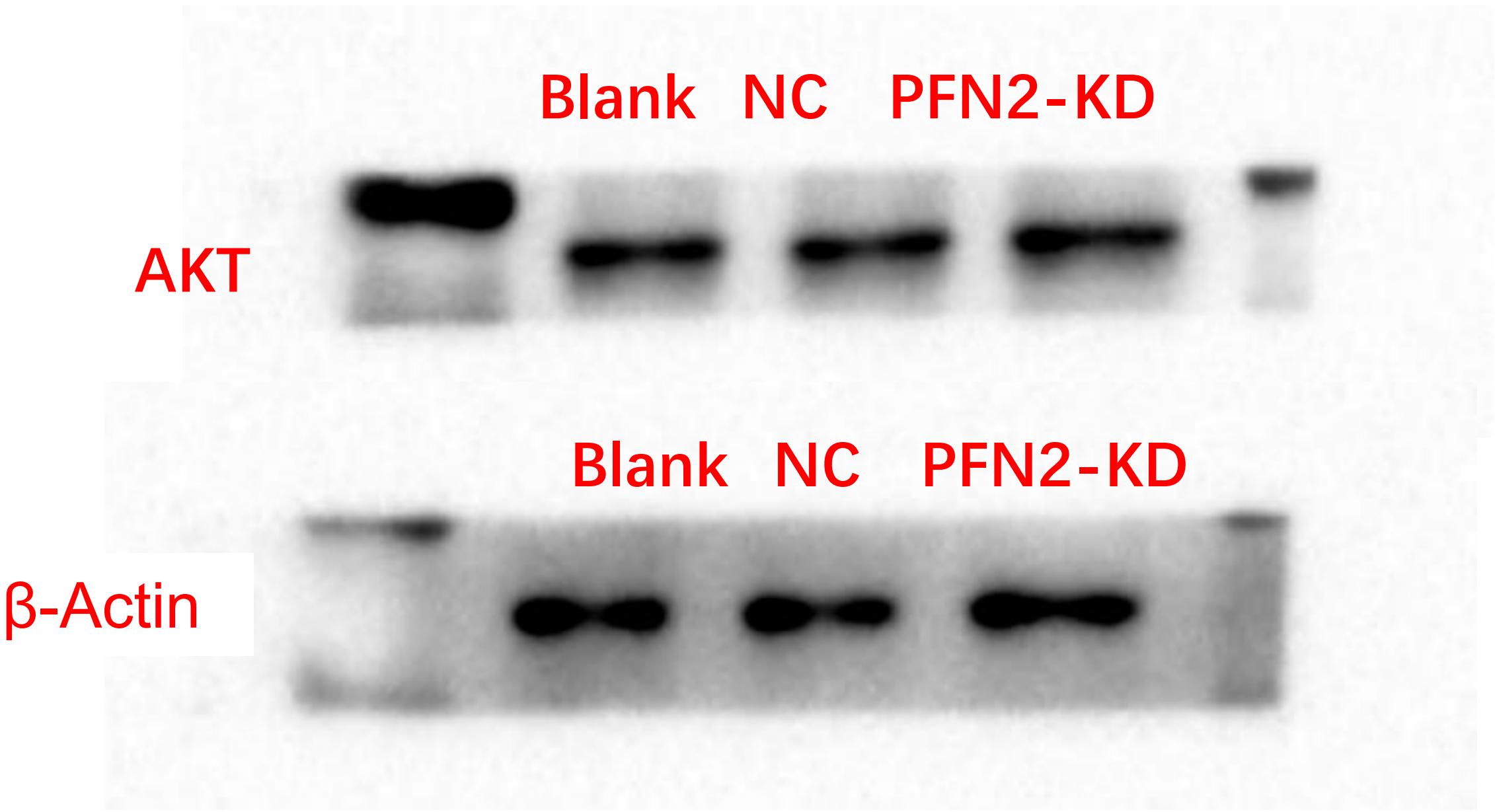

PFN2-KD cell line WB image

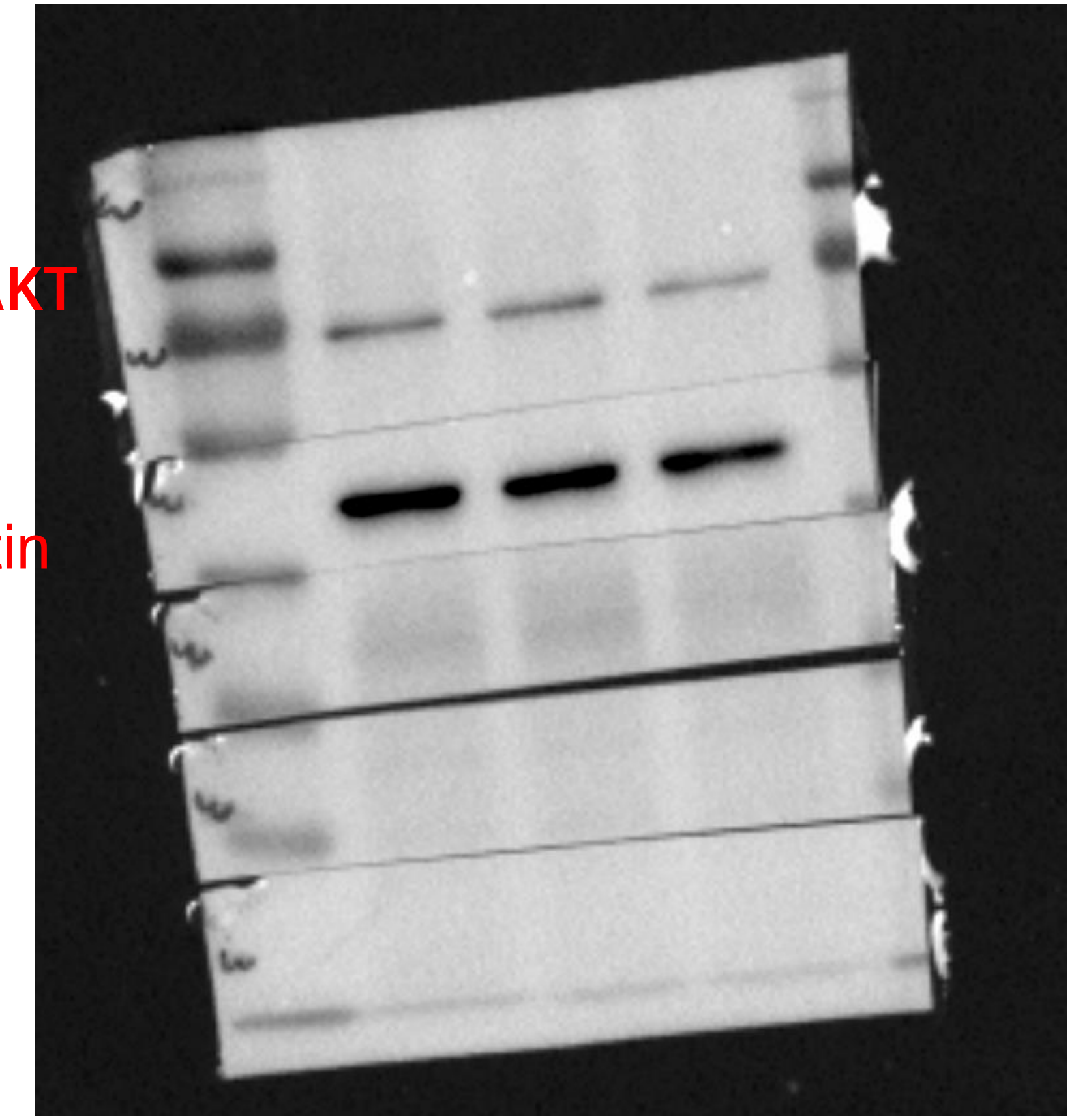

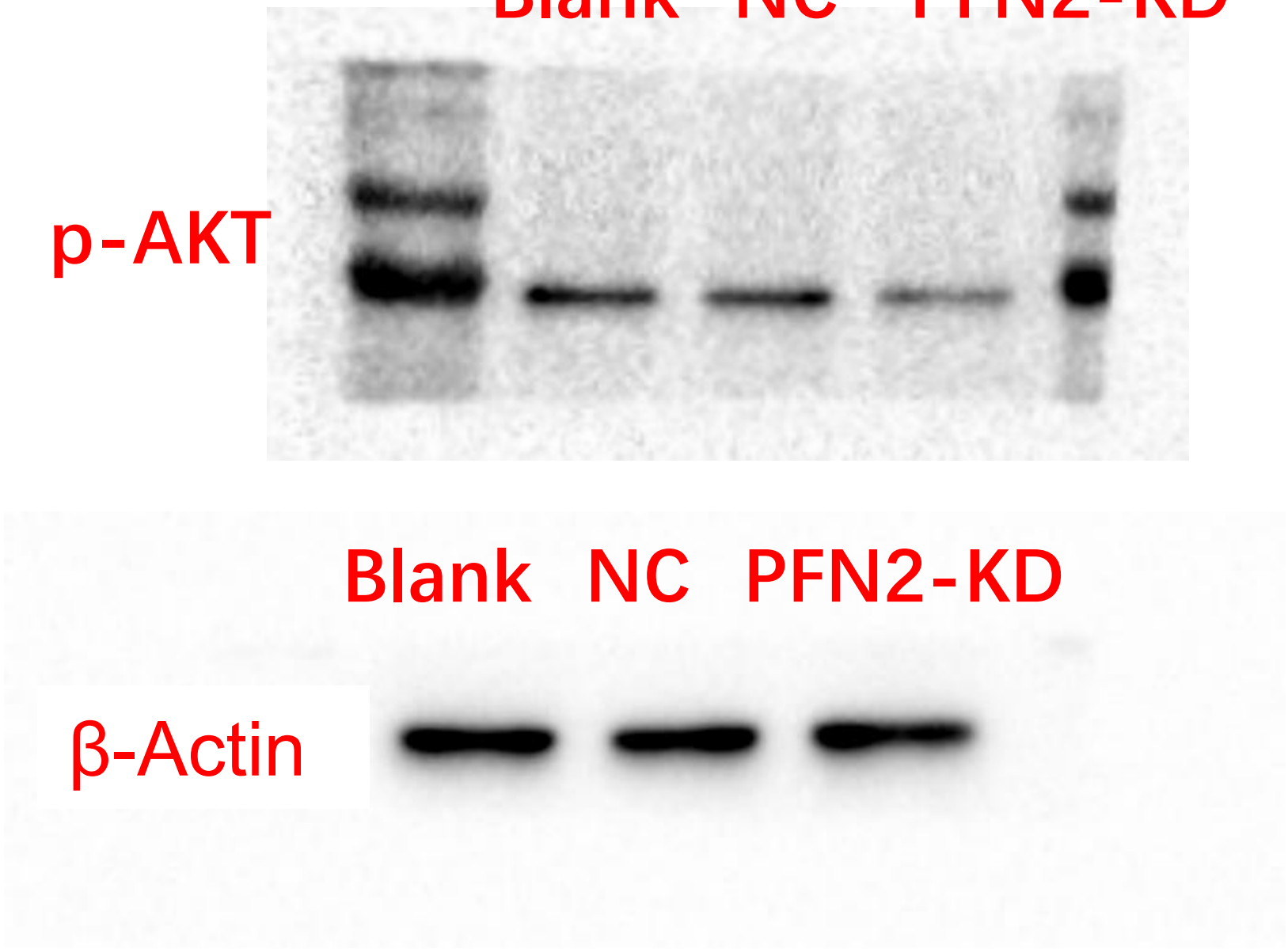

PFN2-KD cell line WB image

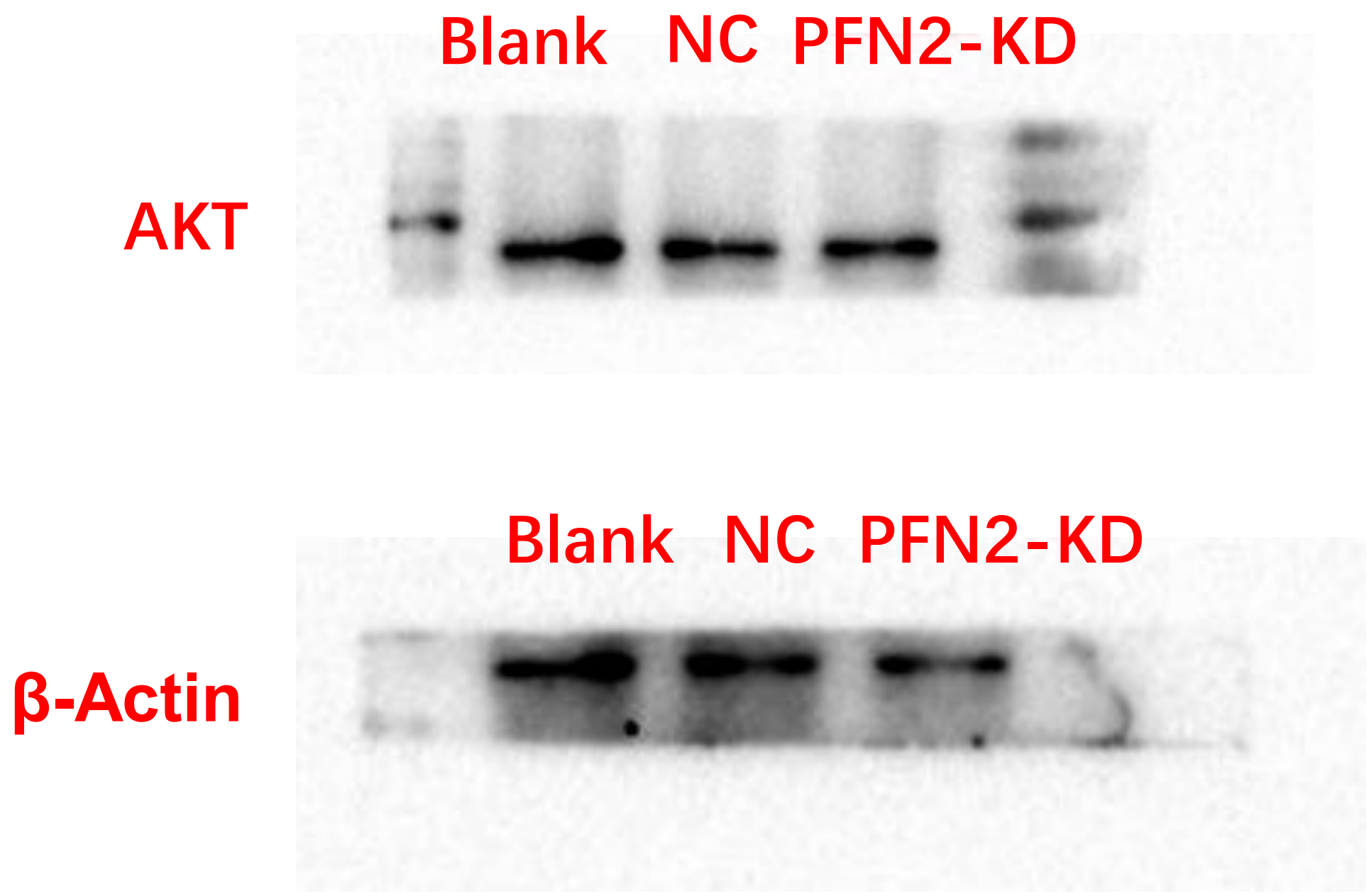

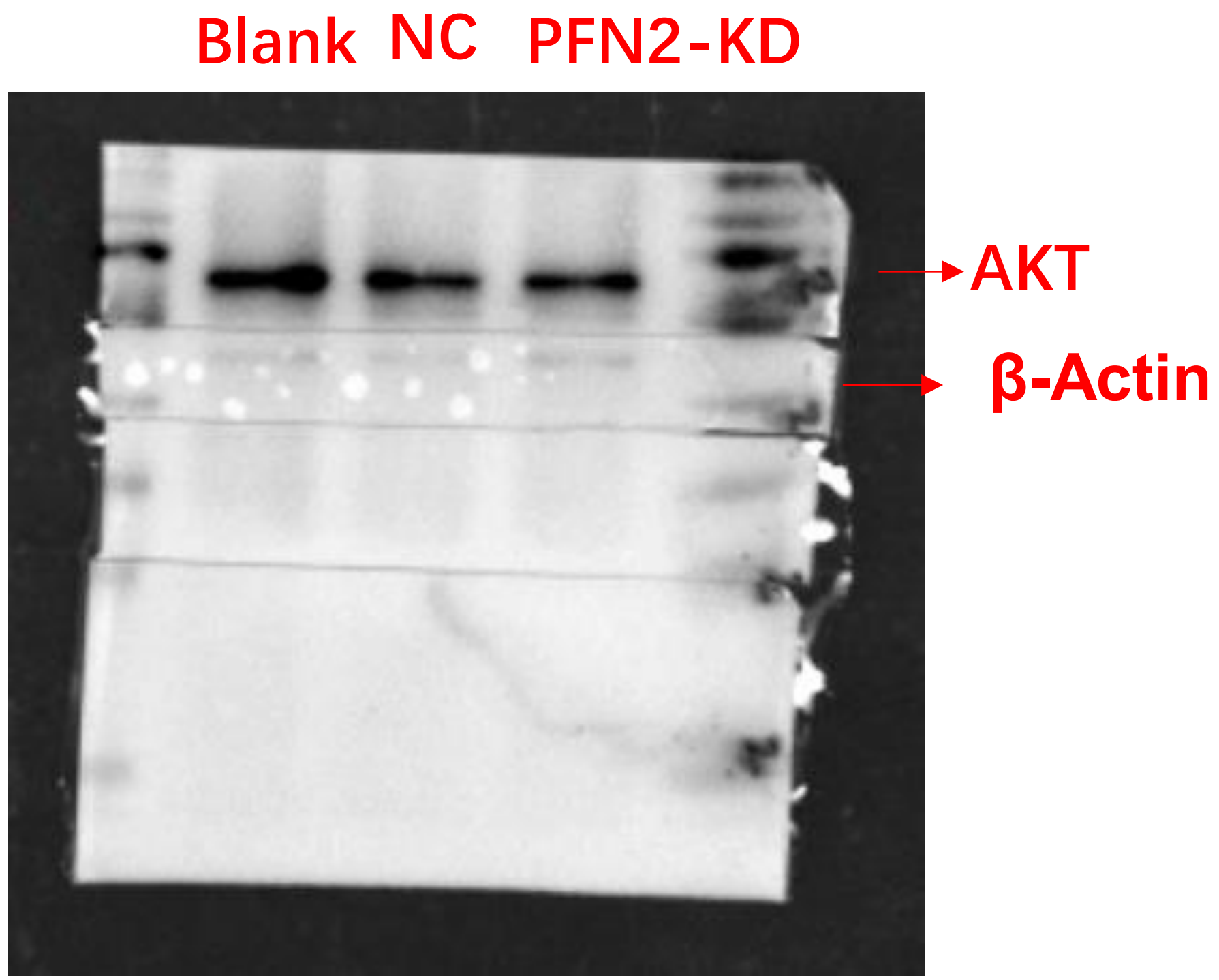

PFN2-KD cell line WB image

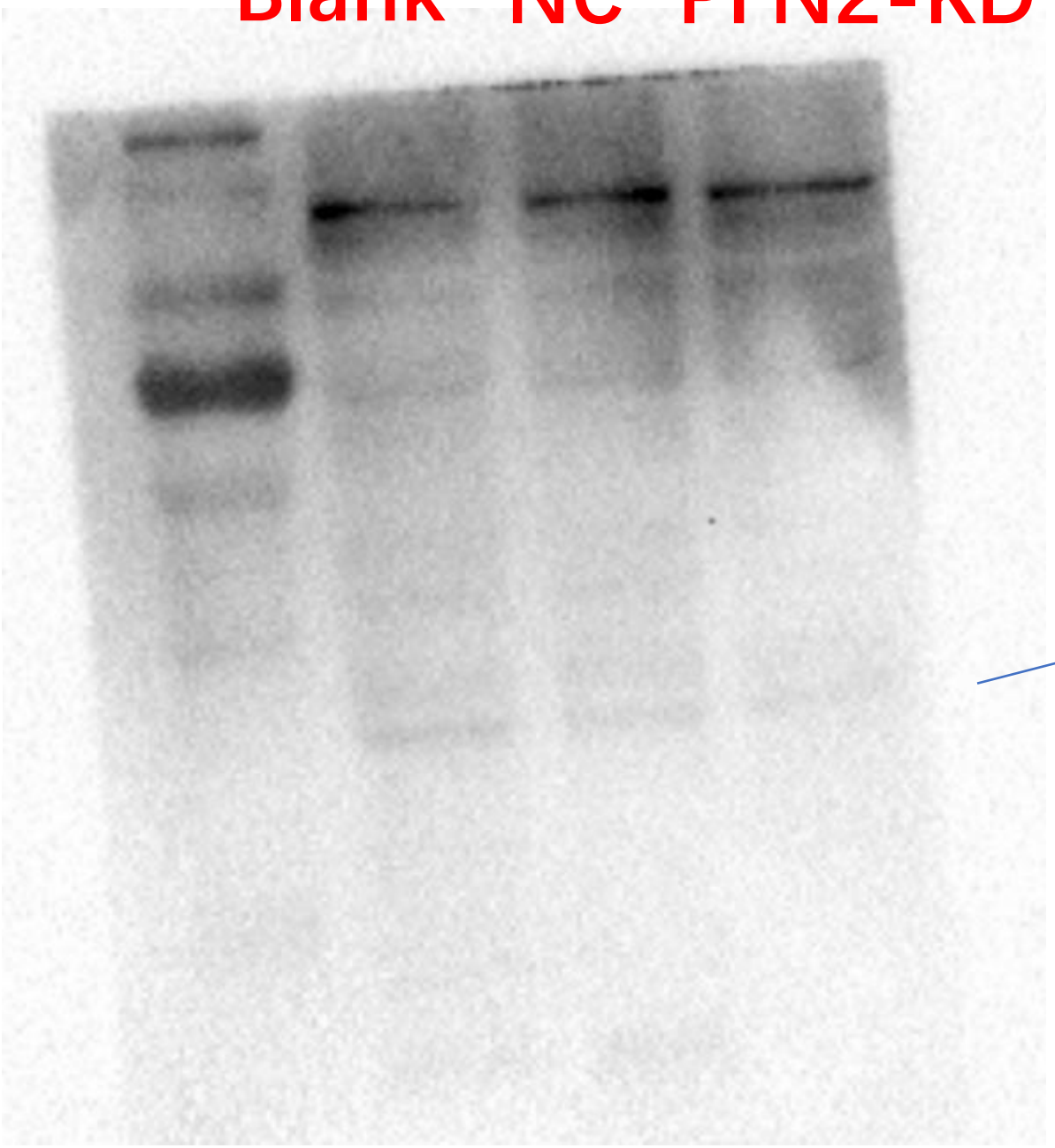

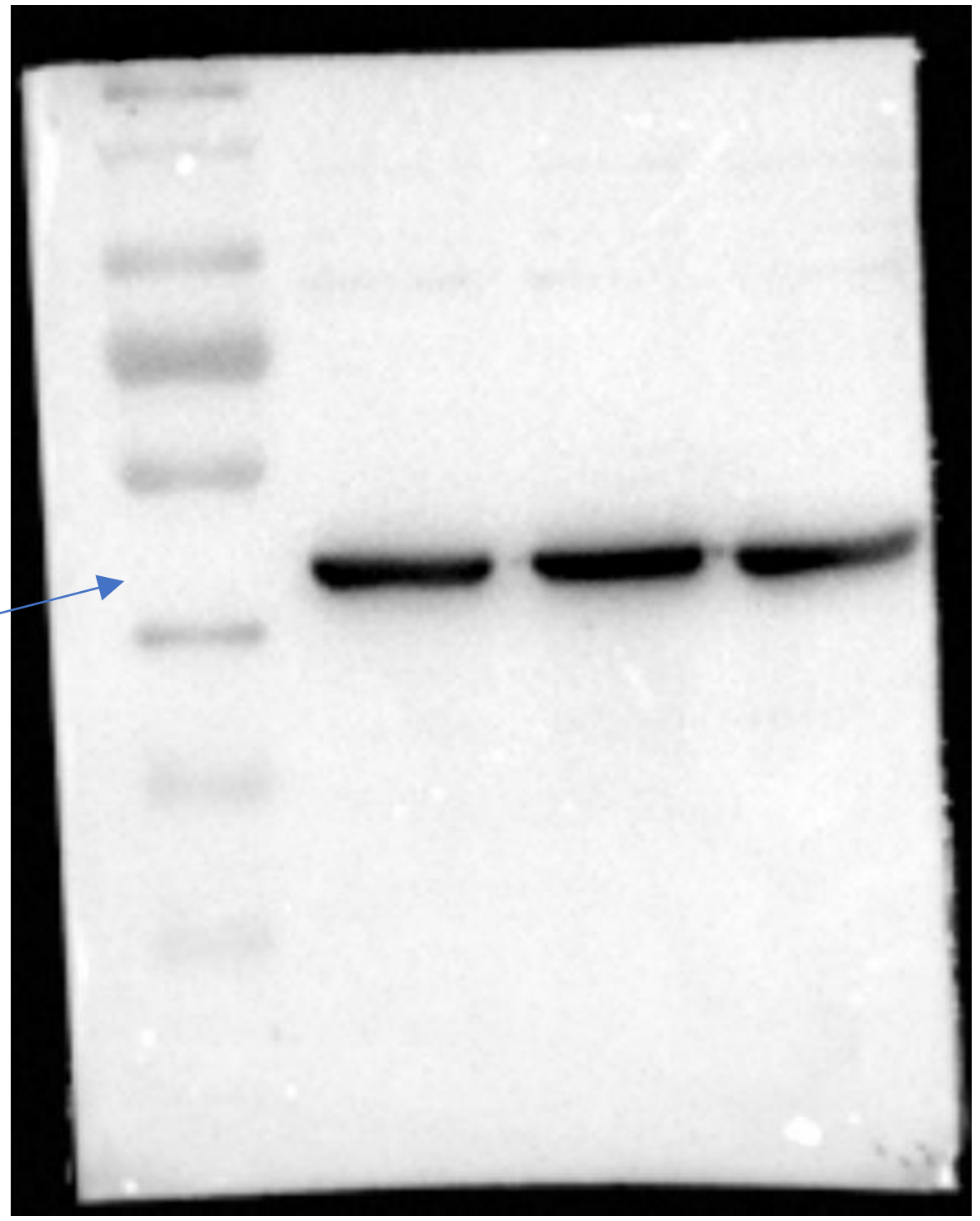

PFN2-KD cell line WB image

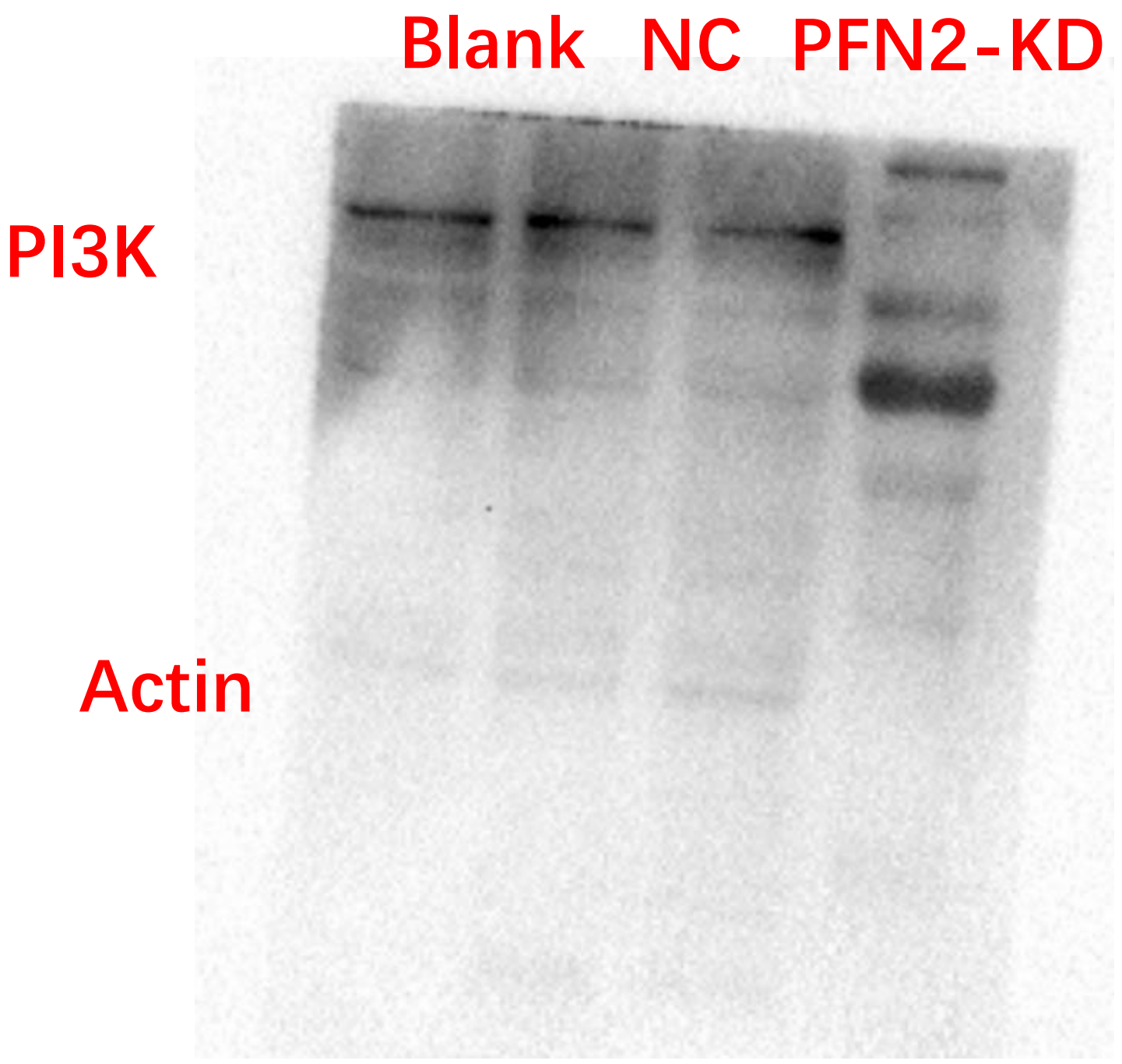

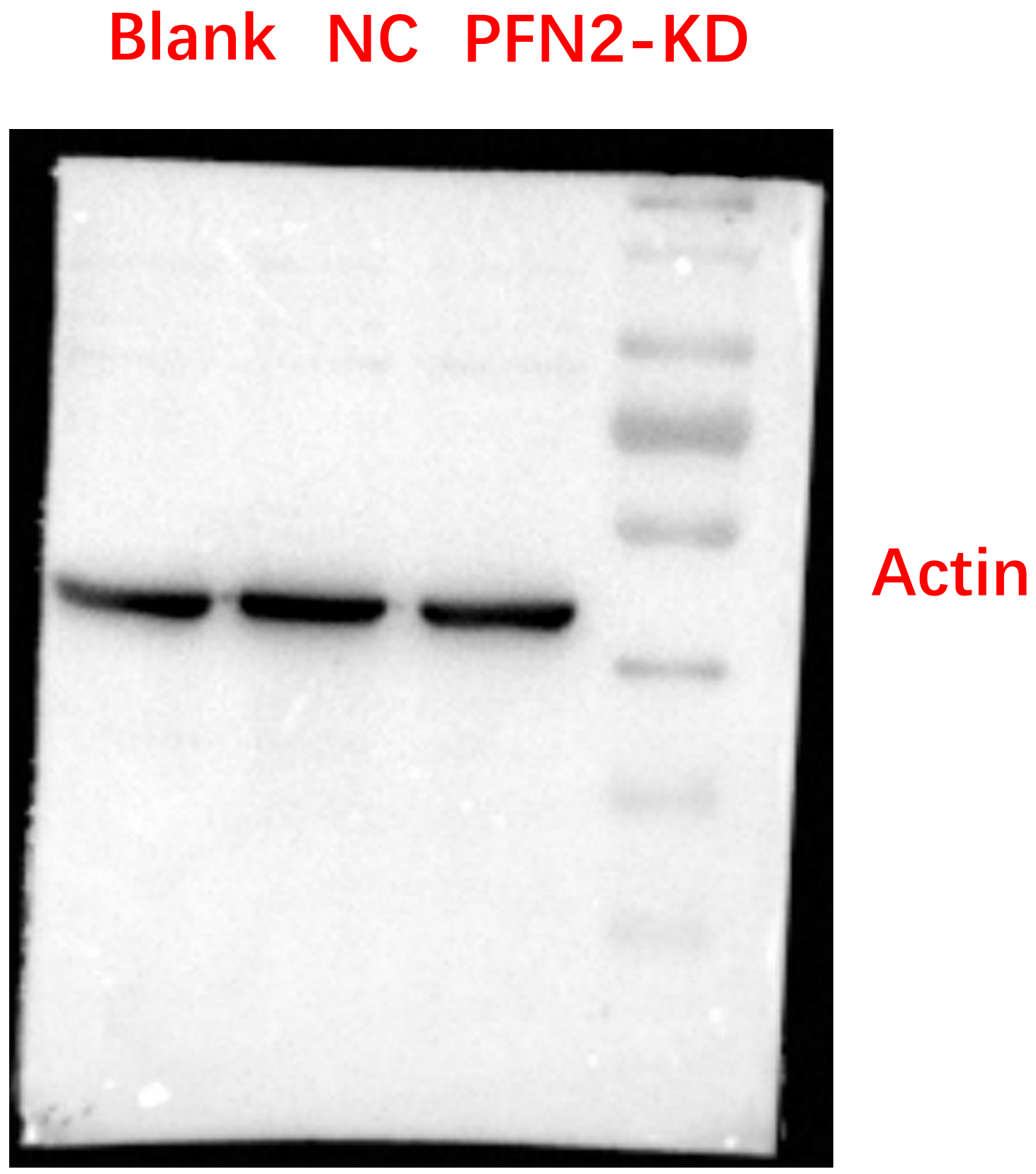

PFN2-KD cell line WB image

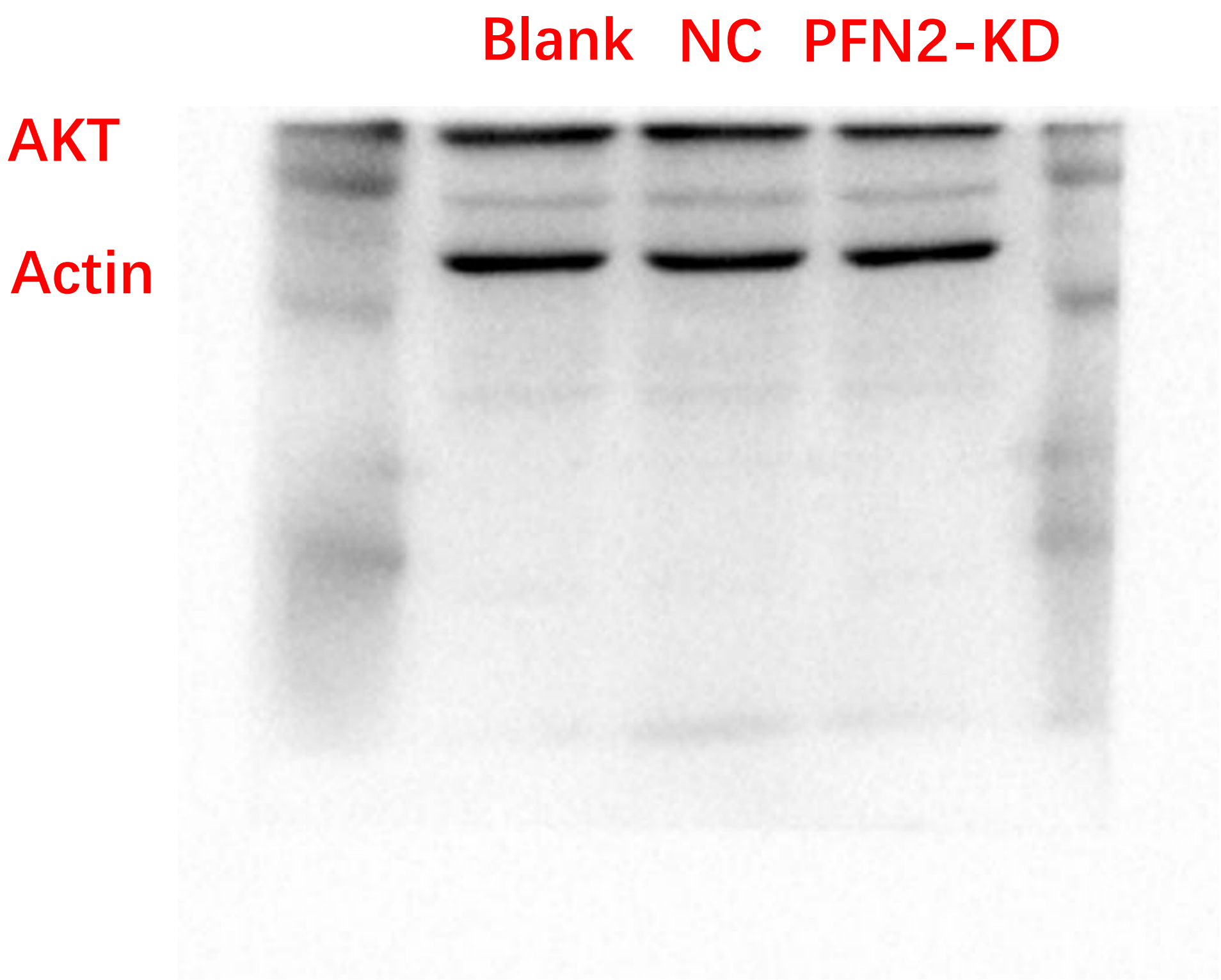

PFN2-KD cell line WB image

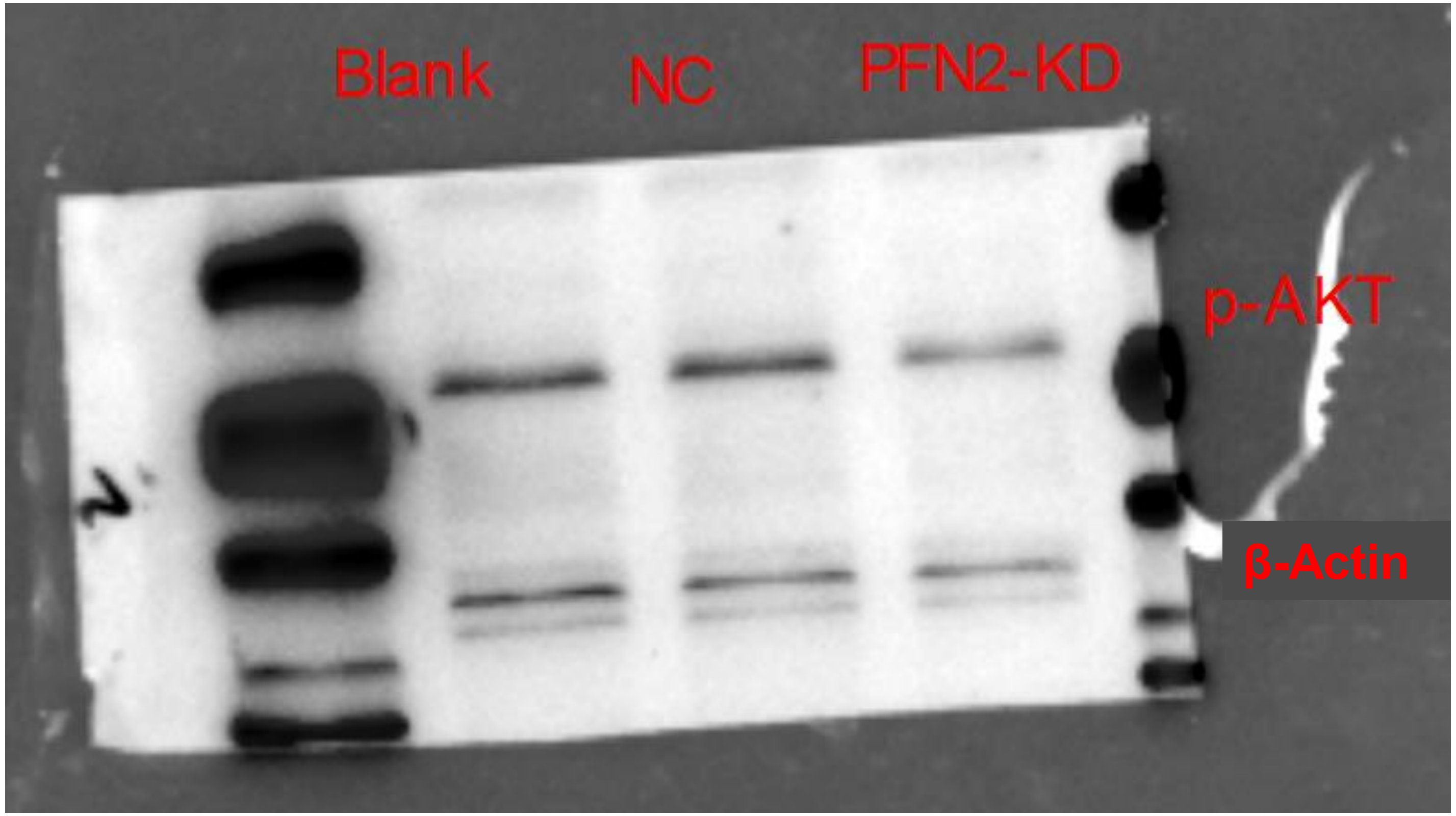

PFN2-KD cell line WB image

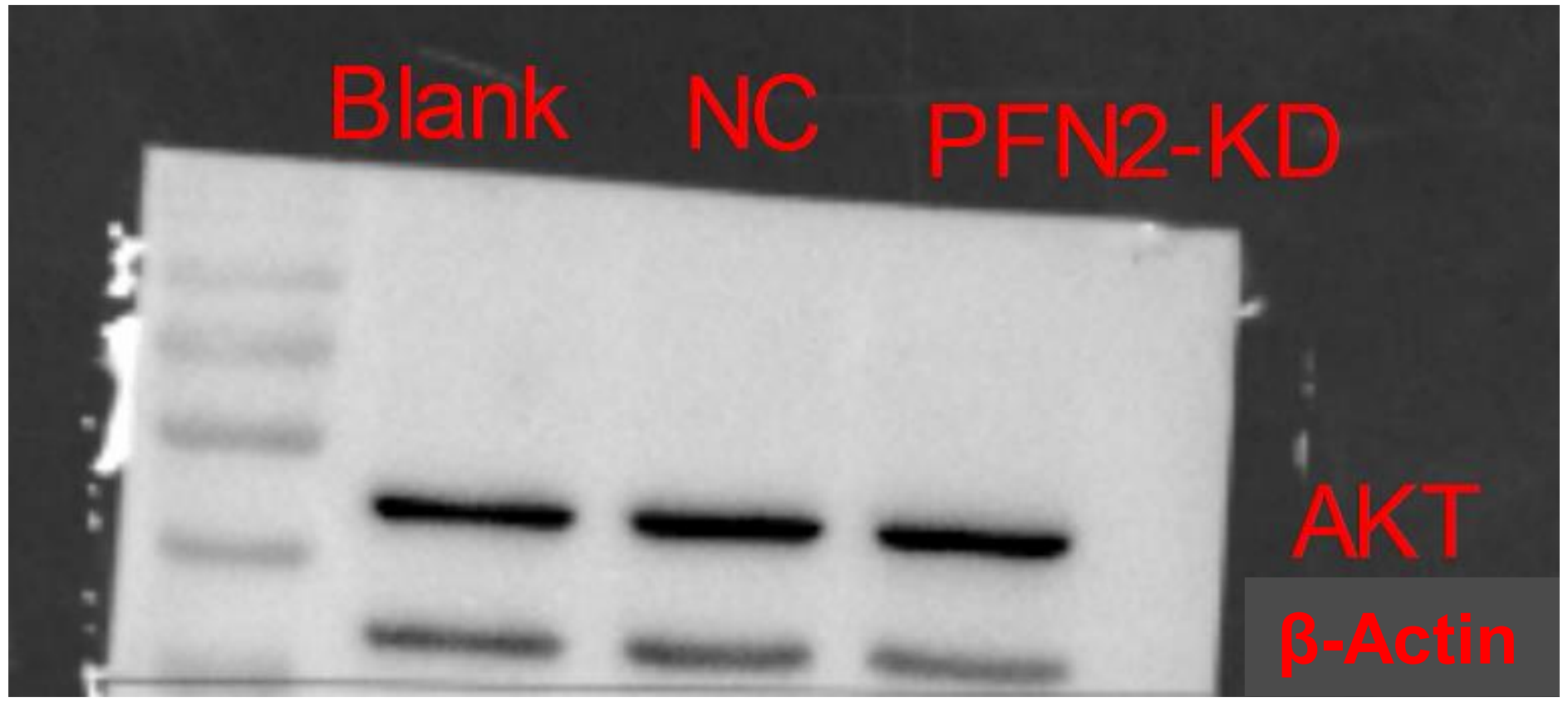

PFN2-KD cell line WB image

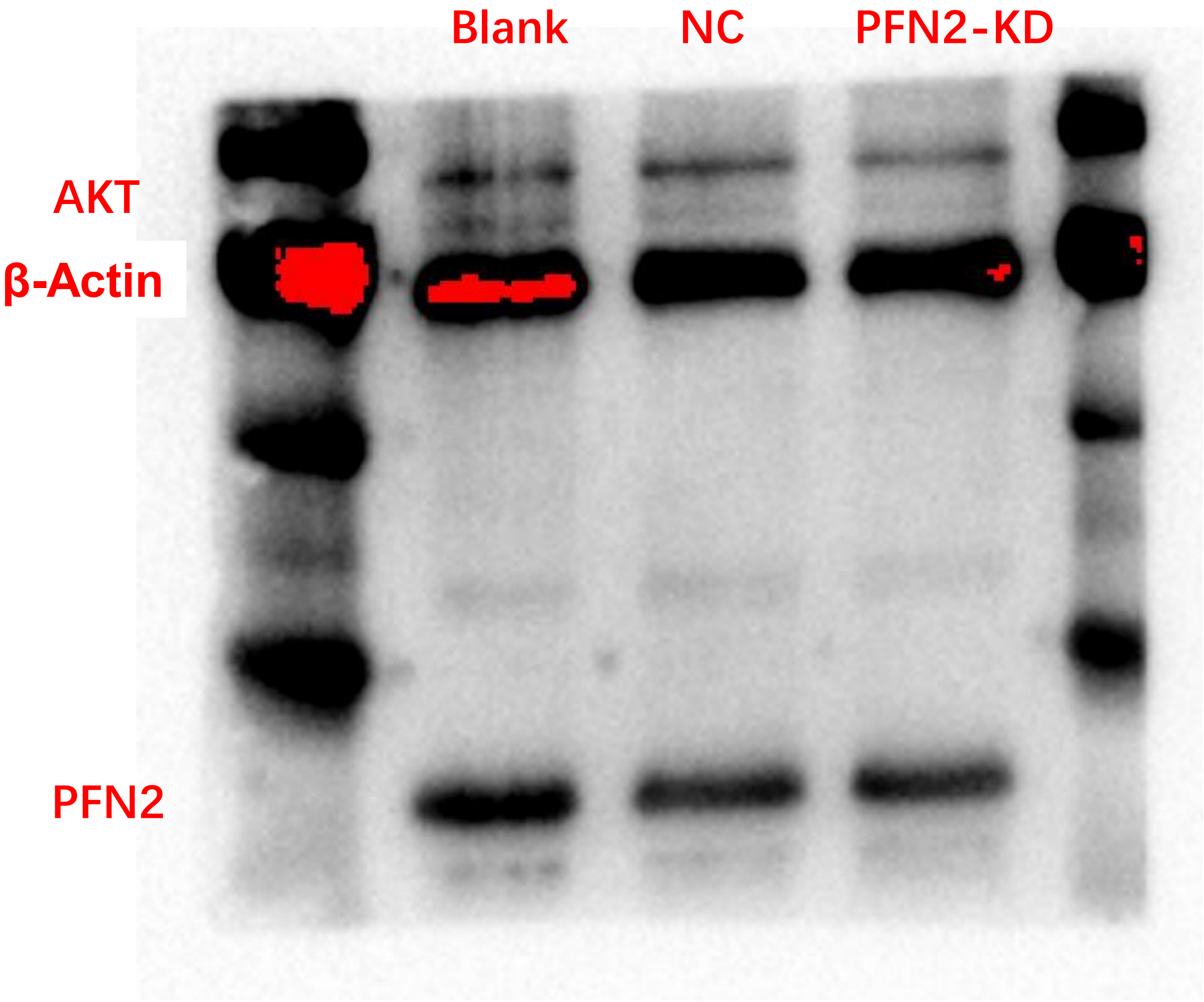

PFN2-Over Express cell line WB image

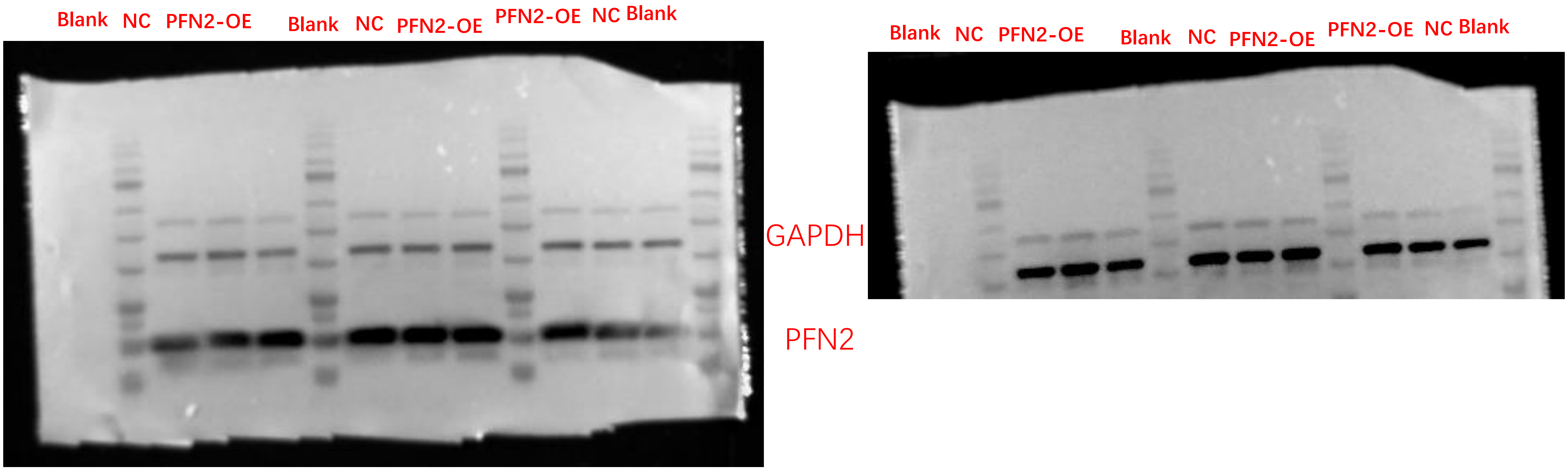

PFN2-Over Express and PFN2-OE+VPA cell line WB image

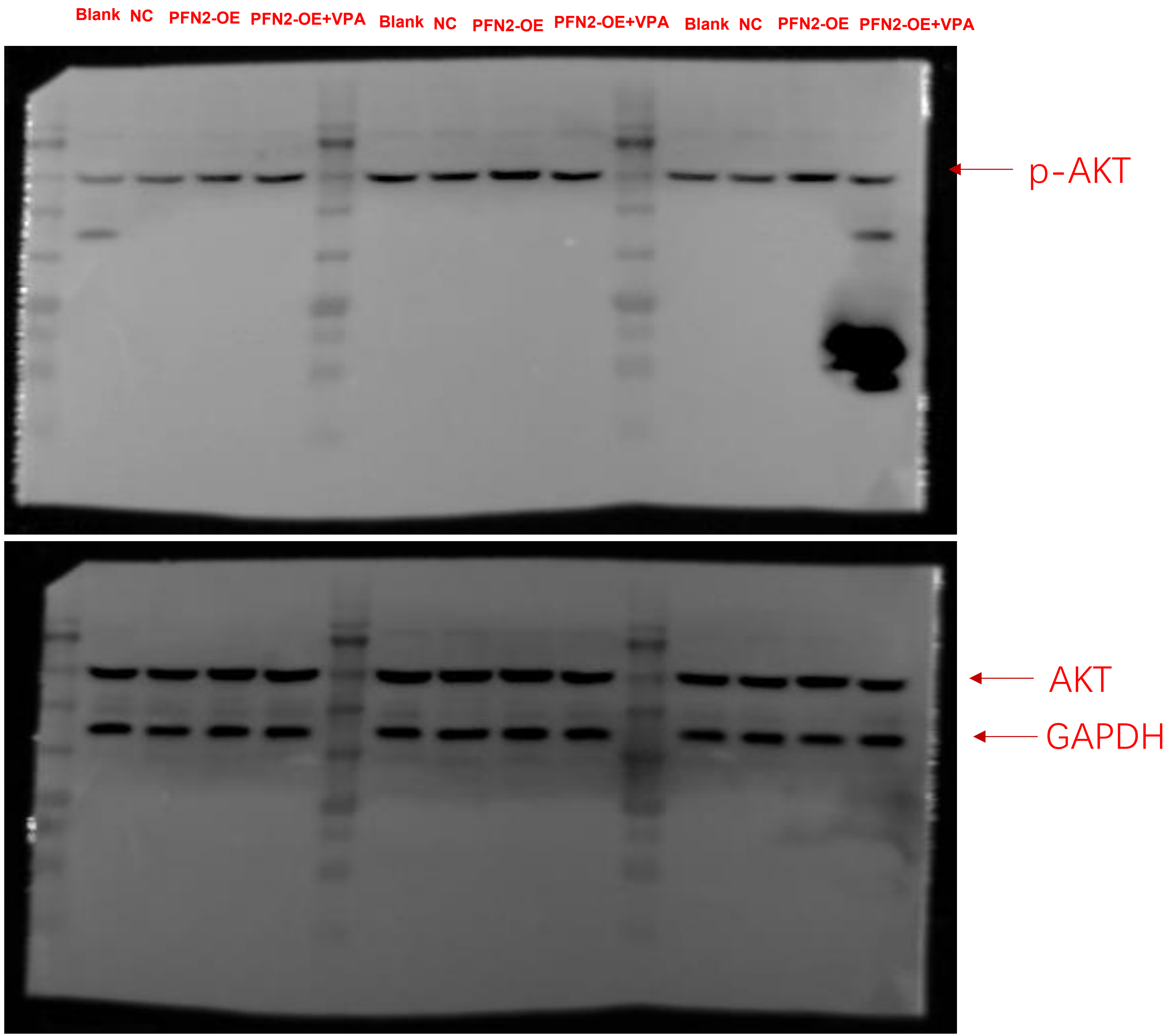

PFN2-Over Express and PFN2-OE+VPA cell line WB image

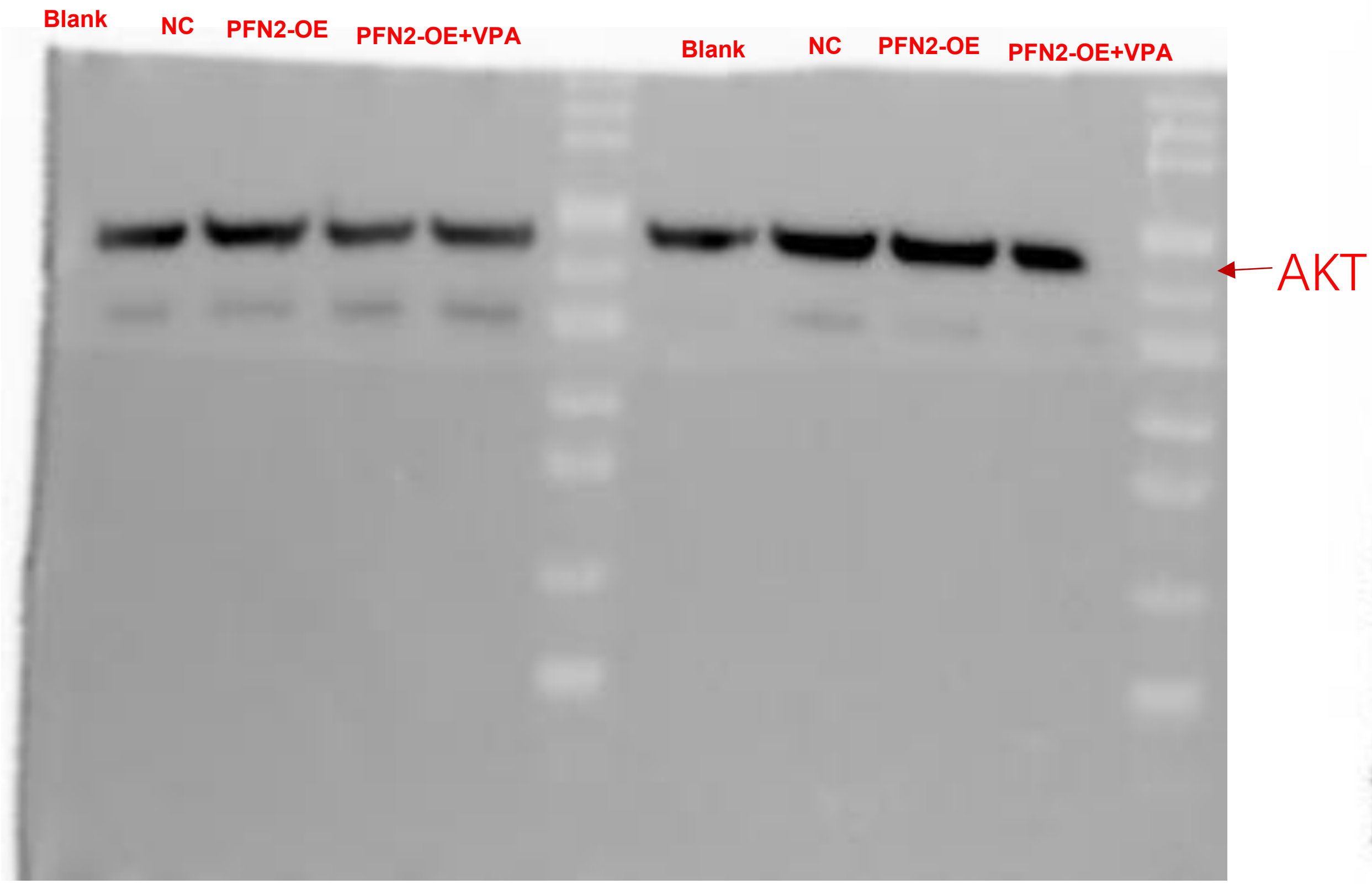

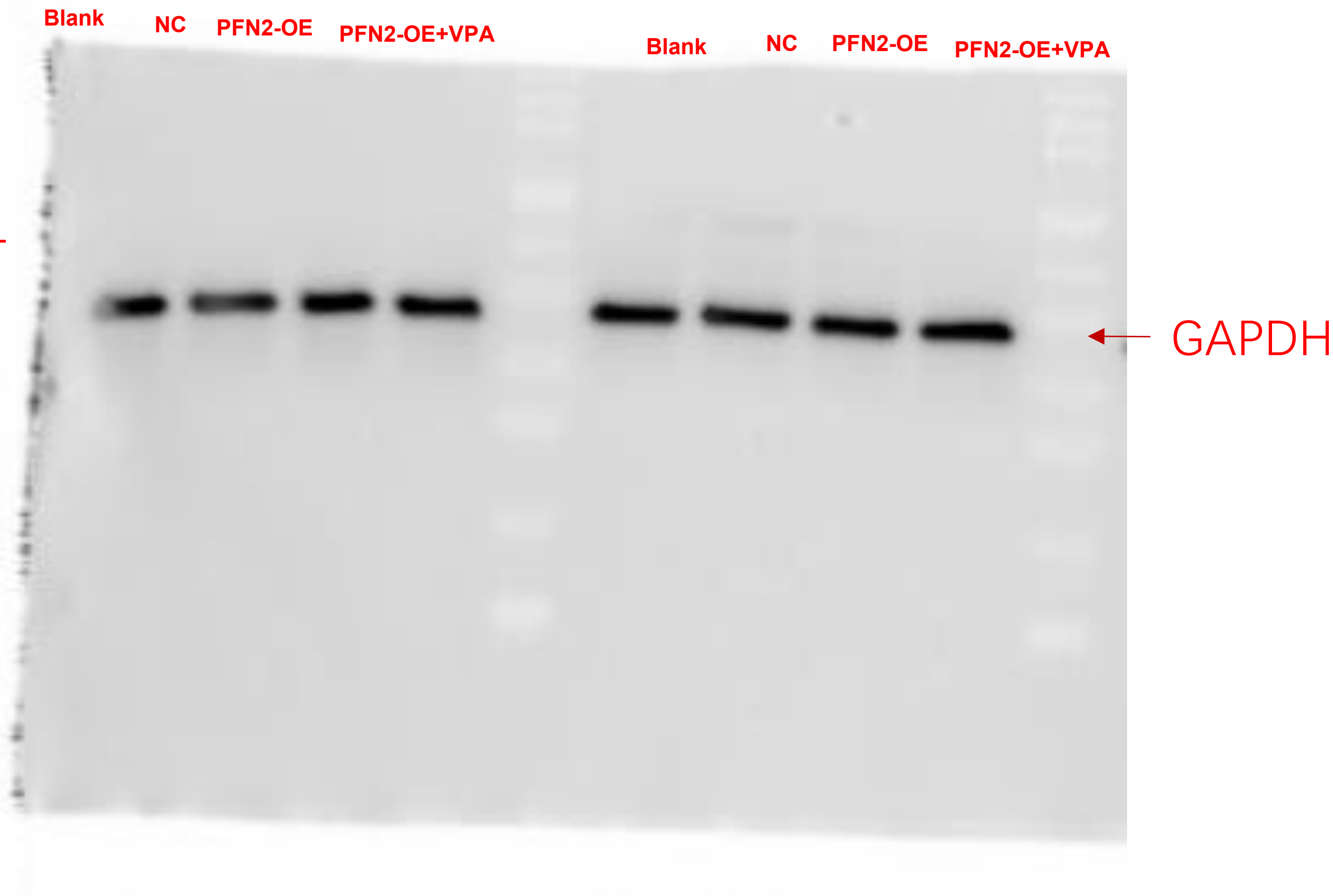

PFN2-Over Express and PFN2-OE+VPA cell line WB image

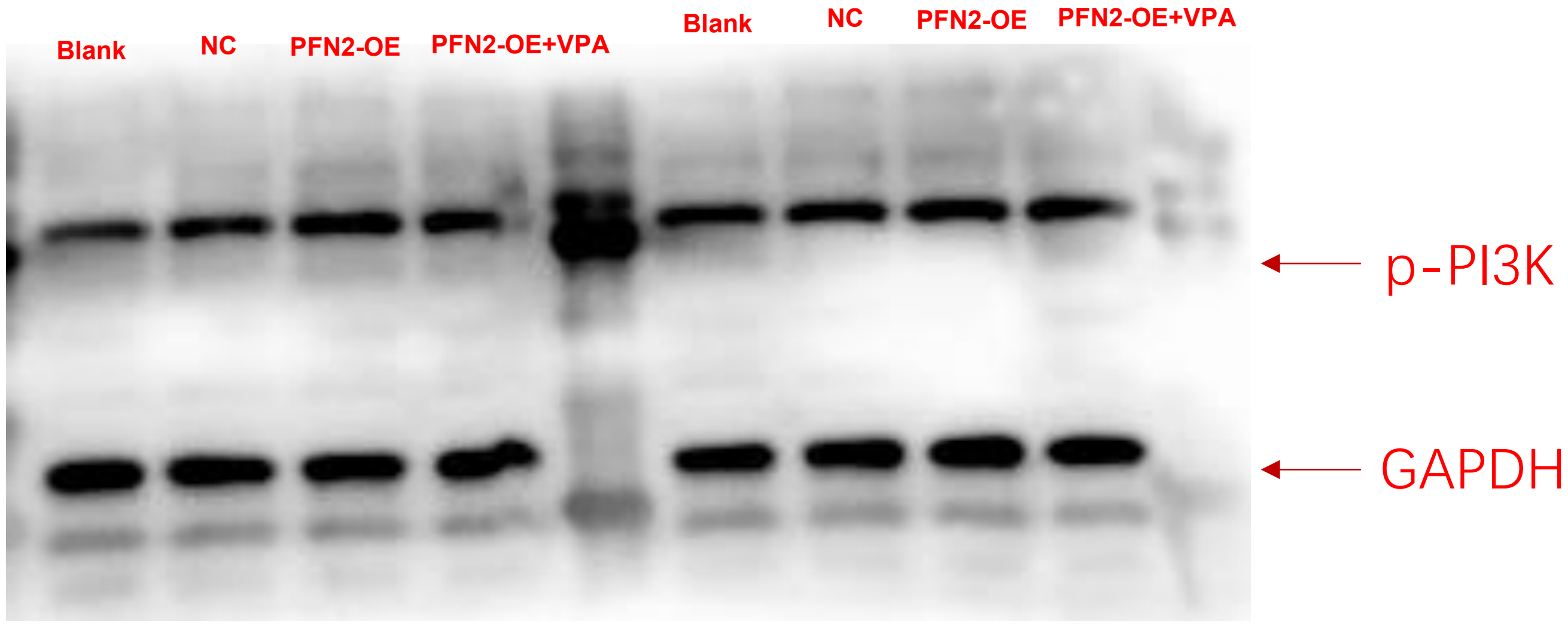

The 29th Gel

PFN2-Over Express and PFN2-OE+VPA cell line WB image

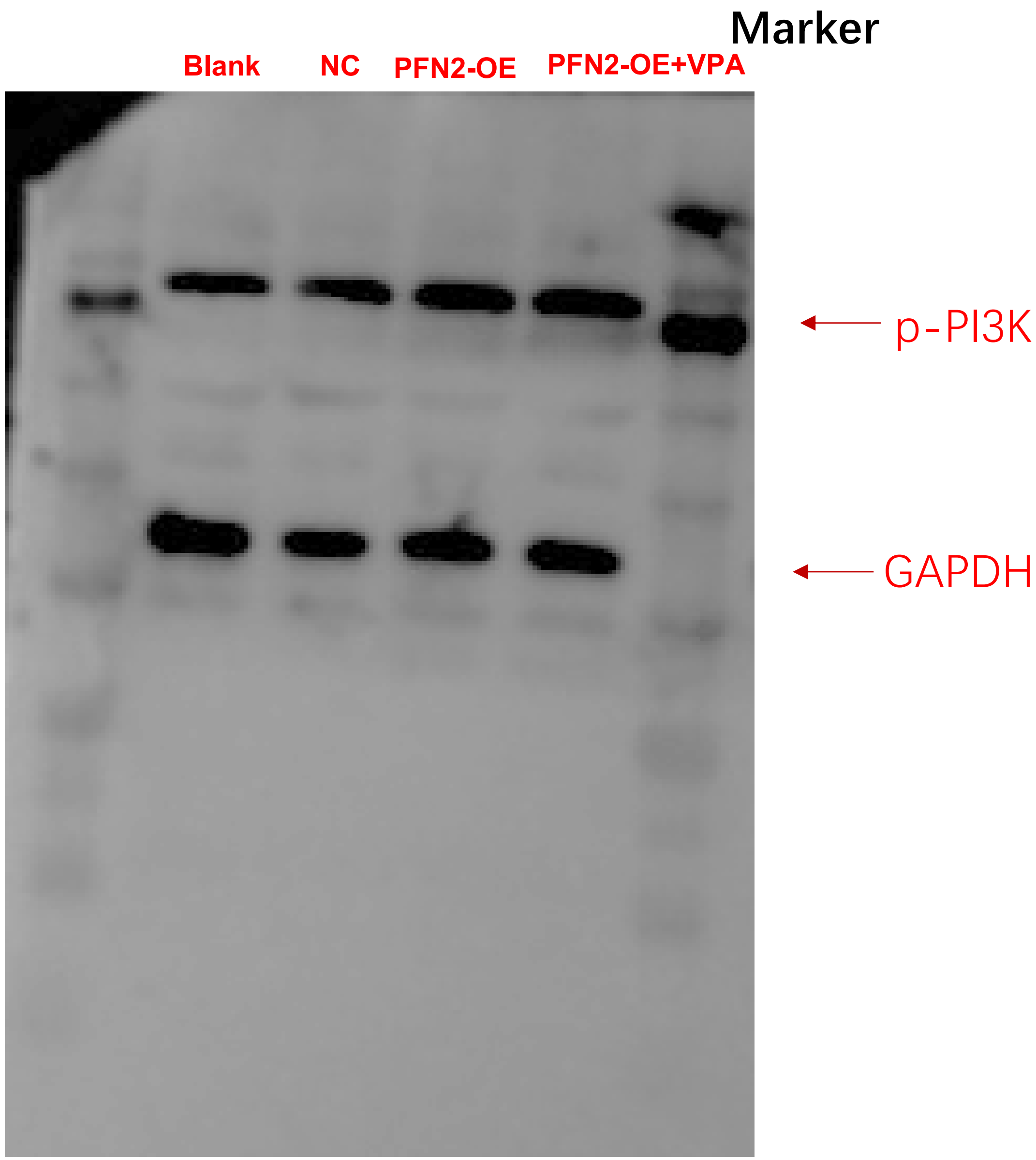

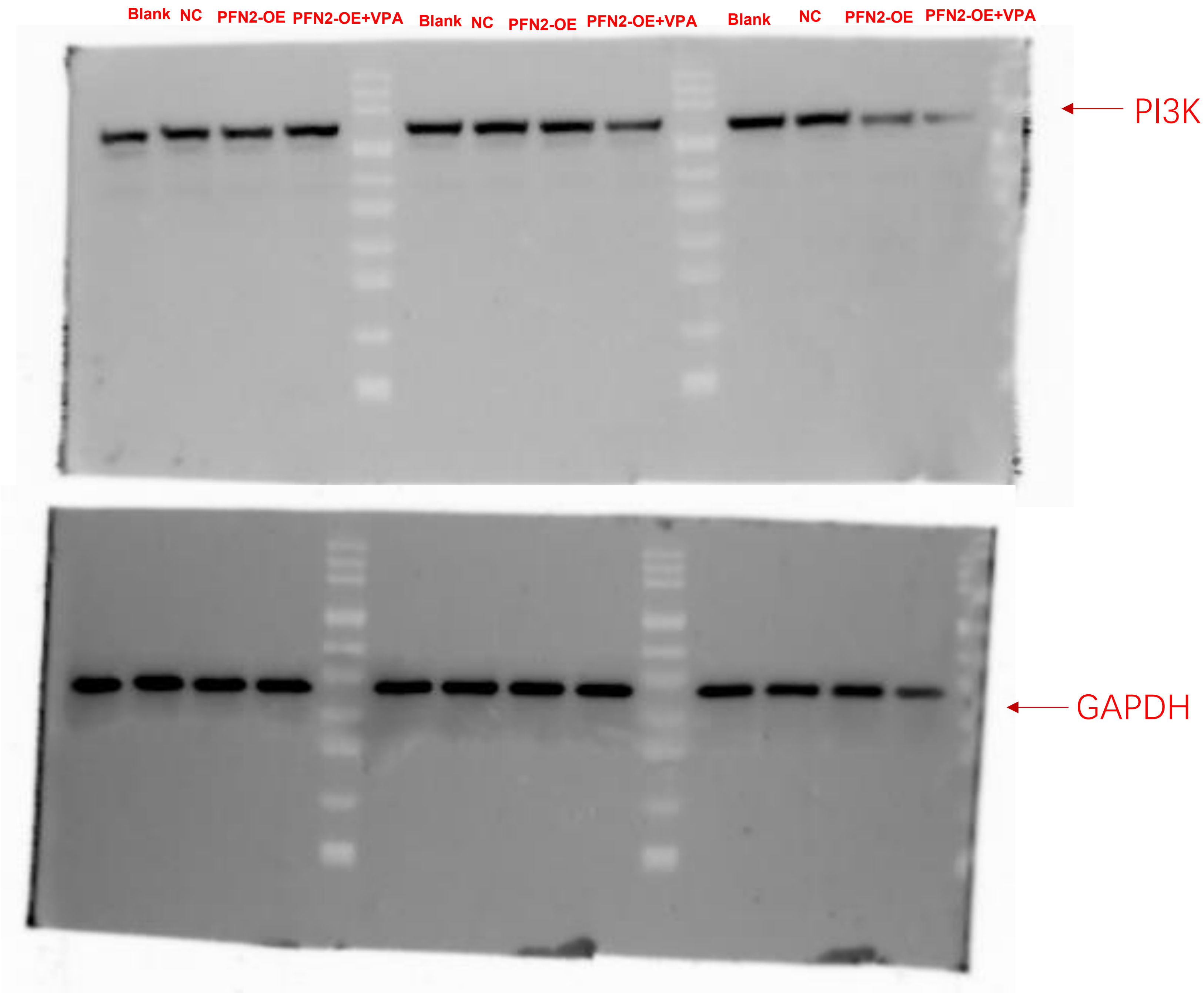
Blank NC PFN2-OE PFN2-OE+VPA Blank NC PFN2-OE PFN2-OE+VPA Blank NC PFN2-OE PFN2-OE+VPA
PI3K
GAPDH
